# Mesopotamian ancient DNA reveals Iron Age integration of heterogeneous Bronze Age genetic ancestries following resettlement

**DOI:** 10.1186/s13059-026-04145-4

**Published:** 2026-06-12

**Authors:** Matthew P. Williams, Rafał Fetner, Yassine Souilmi, Ulrike Bürger, Arkadiusz Sołtysiak, Aidan Richards, Wolfgang Haak, Raymond Tobler, Bastien Llamas, Peter A. Miglus, Alan J. Cooper

**Affiliations:** 1 Australian Centre for Ancient DNA, Faculty of Sciences, Engineering and Technology, Adelaide University, Adelaide, SA 5005 Australia; 2 Biology Department, The Pennsylvania State University, University Park, Pennsylvania 16801 USA; 3 Department of Biological Sciences, Walla Walla University, College Place, Washington, 99324 USA; 4 Department of Bioarchaeology, Faculty of Archaeology, University of Warsaw, Warsaw, 00-927 Poland; 5 The Environment Institute, School of Biological Sciences, Adelaide University, Adelaide, SA 5005 Australia; 6 Institute for Pre- and Protohistory and Near Eastern Archaeology, University of Heidelberg DE, Sandgasse 7, Heidelberg, 69117 Germany; 7 Department of Archaeogenetics, Max Planck Institute for Evolutionary Anthropology, Leipzig, 04103 Germany; 8 Evolution of Cultural Diversity Initiative, Australian National University, Canberra, ACT 2601 Australia; 9 National Centre for Indigenous Genomics, John Curtin School of Medical Research, Australian National University, Canberra, ACT 2601 Australia; 10 Indigenous Genomics, The Kids Research Institute Australia, Adelaide, SA 5000 Australia; 11 Gulbali Institute, Charles Sturt University, Albury, NSW 2640 Australia

## Abstract

**Background:**

The development of complex urban societies in Mesopotamia fundamentally shaped human history, yet the genetic dynamics underlying this process remain poorly understood. Here, we sequence DNA from 17 individuals spanning the Bronze and Iron Ages at Bakr Awa, one of northeastern Iraq's largest ancient settlements located at the border between Mesopotamia and Iran.

**Results:**

Genome-wide analyses reveal substantial genetic heterogeneity during the Bronze Age, characterized by influences of Anatolian, Levantine, and Caucasus/Yamnaya-related ancestries on the local background – complementing archaeological and textual reconstructions of a diverse ethnolinguistic presence at Bakr Awa. This Bronze Age ancestry heterogeneity marks a notable shift from the local Pre-Pottery Neolithic composition – represented by previously published samples from Bestansur, which possess a close affinity to Neolithic central Zagros-related ancestry. The integration of ancient DNA with stable-isotope analysis of 12 individuals reveals multigenerational dynamics and identifies the Zagros Mountains as the most parsimonious recent origin for one of the Bronze Age individuals with Caucasus/Yamnaya-related ancestry. Following the Late Bronze Age site abandonment, reoccupation during the Iron Age involves integrating the preceding Bronze Age divergent ancestries rather than a process of population replacement.

**Conclusions:**

Our findings reveal that heterogeneous ancestries characterize Bronze Age population structure at Bakr Awa, directly mirroring the diverse cultural landscape observed in historical and archaeological sources. Because the subsequent Iron Age transition integrated, rather than replaced, these divergent ancestries, we demonstrate that cultural transitions need not entail large-scale ancestry transformations. Together, these findings capture Mesopotamia's role as a "melting pot" of ancient Near Eastern ethnolinguistic groups.

**Supplementary Information:**

The online version contains supplementary material available at 10.1186/s13059-026-04145-4.

## Background

**Fig. 1:**
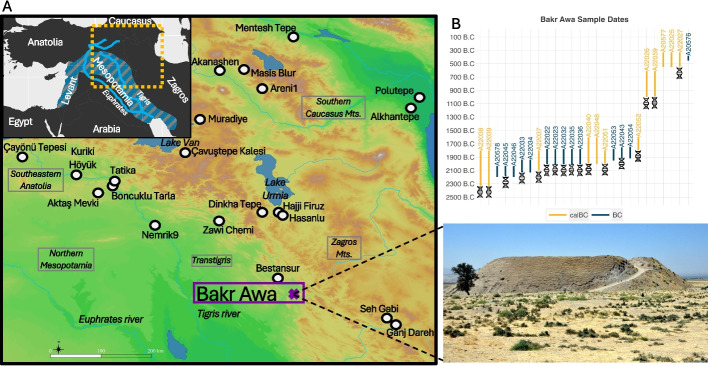
Geographic and chronological context of Bakr Awa. **A** Geographic map of the ancient Near East. Zoomed-in topographic plot showing southeastern Anatolia, northern Mesopotamia, Lesser Caucasus, and northwestern Zagros Mountains correspond to the dashed-box cut-out. Circles mark published comparative sites, and the triangle locates Bakr Awa. Photograph: southeastern slope of Bakr Awa citadel photo courtesy of Ulrike Bürger. **B** Sample date ranges shown as Mesopotamian Middle Chronology (blue, BC) or calibrated radiocarbon dates (yellow, calBCE). Double helix symbols denote successful DNA sequencing

Mesopotamia, encompassing the plains surrounding the middle and lower Euphrates and Tigris rivers (Fig. 1A), was home to some of the earliest sedentary communities in the world that developed subsistence systems based on cereal cultivation and animal husbandry [1, 2]. Ancient DNA from the northern Fertile Crescent reveals that by the Pre-Pottery Neolithic (c. ~ 12–8.5 kiloanni before 1950 CE (ka BP)) human groups occupying permanent settlements throughout northern Mesopotamia had ancestral ties to peoples inhabiting the surrounding Taurus and Zagros Mountains [3, 4]. Over the course of subsequent millennia, Mesopotamia would become a melting pot of ancient human communities arriving through both voluntary and coerced migrations, resulting in a rich tapestry of ethnic, cultural, religious, and linguistic diversity [5–8].

The third to second millennium BCE marks an important period in Mesopotamian history. By this time, urbanized stratified societies had already been established [9–11] and writing began to take the form of historical texts, in addition to administrative and priestly inscriptions [12, 13]. Textual sources document diverse ethnolinguistic groups inhabiting Mesopotamia, including expansions from peripheral regions such as the Caucasian origin of the Hurrian language [14, 15], the spread of Akkadian into Mesopotamia during the Early Bronze Age [16], and the complex migration and enculturation of Amorites into Mesopotamian society from the Upper Euphrates around the late third millennium BCE [17]. Archaeological and historical records reveal the second millennium BCE came to a catastrophic end – colloquially referred to as the Late Bronze Age collapse (c. ~ 1200 BCE) – triggered by devastating climatic events, possible migratory raiding confederates, and internal societal unrest [18, 19]. Cumulatively, this resulted in a significant shift in territorial control across the entire ancient Near East. The Iron Age emerged from this calamitous period, marking the rise of empires that exerted their influence over vast territories and on a scale previously unseen.

Given its rich history of war, trade, migration, and cultural exchange between its surrounding regions, Mesopotamia serves as an ideal region to assess the degree to which these socio-cultural interactions identified from archaeology and textual sources contributed to shifts in genetic composition over time. However, due to the harsh Mesopotamian climate, human remains are not well-preserved, and ancient DNA records are sparse, resulting in a generally limited understanding of the genetic history of the region. Genetic ancestry of Bronze Age northern Mesopotamia and southeastern Anatolia is known from only two Tigris River sites – Şırnak (Tatika, five samples, late fourth-early third millennium BCE) and Nemrik 9 (one sample, mid-late second millennium BCE) (Fig. 1A) – which share similar levels of Caucasus Hunter-gatherer-related (CHG) ancestry but differ in their Anatolian versus Levantine Neolithic-related proportions [20]. Similar ancestry profiles in first millennium BCE individuals from Aktaş Mevki (Mardin) and Kuriki Höyük (Batman) (Fig. 1A) suggest population continuity in northern Mesopotamia and southeastern Anatolia across three millennia [20], though whether larger Mesopotamian population sizes buffered against significant ancestry shifts despite migrations remains unclear.

Situated on a fertile valley connecting the Mesopotamian plains and the Zagros mountains (Transtigris, Fig. 1A), Bakr Awa is the largest archaeological mound in the southern part of the Shahrizor Plain (Additional file 1: Note S1). Its strategic position established it as a conduit for trade and migration between Mesopotamia and Iran, making it well-suited for assessing the genetic impact of inter-regional interactions. From the late fourth to the first millennium BCE, Bakr Awa's archaeological record reveals a complex tapestry of cultural influences, reflecting the dynamic socio-political landscape of ancient Mesopotamia. During its oldest occupation in the Uruk Period (c. 4000–3100 BCE), Bakr Awa appears to have been within the sphere of southern Mesopotamian cultural influence. However, during the Early Dynastic Period (c. 2900–2350 BCE), Bakr Awa shifted to a more local tradition, followed by an adoption of northern Mesopotamian architectural styles in the Akkadian Period (c. 2350–2150 BCE). During the late third millennium BCE, southern Mesopotamian cultural influence reappeared at Bakr Awa, coinciding with the emergence of the Ur III kingdom [21–26]. These cultural shifts illustrate regional socio-political dynamics impacting Bakr Awa for two millennia between 4000 and 2000 BCE.

In the Late Bronze Age, Bakr Awa appears to have functioned as a pivotal administrative center, as evidenced by its archive of Akkadian and Hurrian cuneiform texts, revealing the site's linguistic diversity during the mid-second millennium BCE [24, 27]. A devastating fire brought an end to Bakr Awa’s Bronze Age occupation in the 15th-14th century BCE [24] and whilst the subsequent Iron Age occupation remains poorly understood due to later disturbances, the discovery of reddish Slipped Ware in the first millennium BCE, with similarities to northwestern Iranian Urartian and Median pottery styles of the 8th-6th centuries BCE, provides a crucial insight into the site's continued cultural connections to the north [22, 24, 28, 29]. As such, Bakr Awa's archaeological record reveals extensive cultural shifts and connections across the Near East, making it ideal for studying the genetic impact of interactions between ancient Near Eastern societies. Here, we apply genome-wide and stable-isotope analyses to investigate whether Bakr Awa's cultural diversity is reflected in its inhabitants' genetic ancestry and mobility patterns.

In this study, we document the arrival of multiple non-local ancestries to the Bronze Age Transtigris at Bakr Awa, resulting in ancestry discontinuity from the previously published local Pre-Pottery Neolithic inhabitants. One of the non-local ancestry components shared amongst all Bakr Awa individuals possesses similarities with populations located in Anatolia and northern Mesopotamia, suggesting these regions as potential sources of migrant ancestry. The remaining non-local ancestries driving Bronze Age population structure – with individuals possessing differential affinities to ancestries from the Caucasus and the Levant – is consistent with the archaeological picture of Bakr Awa functioning as an urban centre and a conduit for regional trade and migration. Our interdisciplinary approach revealed complex migration dynamics where one Early to Middle Bronze Age male possesses Yamnaya-related Caucasus ancestry in both Y chromosome and genome-wide signatures, yet stable-isotope data suggest he was a recent migrant to Bakr Awa from the Zagros mountain region. Following site abandonment, Iron Age individuals appear to largely descend from the preceding Bronze Age community, with their ancestry reflecting genetic integration of the preceding structure rather than population replacement – suggesting that sociocultural dynamics driving the Bronze Age population structure had shifted.

## Results

### Bioarchaeological data for the Bakr Awa Bronze and Iron Age

In dedicated clean rooms at the Australian Centre for Ancient DNA, we sampled and powdered 28 human remains from Bakr Awa (25 petrous bones and three teeth) uncovered during excavations by the University of Heidelberg spanning over six seasons since 2010 (Additional file 1: Note S1). We extracted DNA [30] and generated double-stranded partial uracil-DNA glycosylase (UDG) libraries [31, 32] following established protocols (see “Methods”). Of these, 27 libraries that were successfully amplified, we performed in solution hybridization using the Daicel Arbor Biosciences Prime Plus kit (Arbor-PP) [33, 34], which targets the Agilent Legacy ~ 1.24 million (1240k) genome-wide single-nucleotide polymorphisms (SNP) panel (Legacy 1240k) [35–38]. However, in-solution hybridization capture is subject to documented technical bias, characterized by an artificial attraction between individuals captured with the same technology, which can compromise genetic affinity analyses when using cross-technology datasets [33, 34, 39]. To mitigate these limitations, we followed published guidelines for the co-analysis of the Arbor-PP data with Legacy 1240k and shotgun data [33], which we describe in more detail below. Where necessary, deviations from these published guidelines are explicitly noted at their point of implementation. Sequencing on an Illumina NovaSeq yielded between 6,357 and 908,706 SNPs overlapping the 1240k panel per individual (Additional file 2: Data S1).

We excluded 10 individuals from downstream analyses based on four ancient DNA authenticity criteria: damage patterns in the final nucleotide below typical ranges for authentic ancient DNA [31]; elevated heterozygosity on the male X-chromosome detected through ANGSD [40, 41]; anomalous chrX-chrY ratios of mapped reads (https://github.com/TCLamnidis/Sex.DetERRmine) (Additional file 1: Fig. S1); and elevated mitochondrial heterozygosity identified through MitoSuite [42] (Additional file 2: Data S1). For population-based analyses, we applied additional filters: excluding low-coverage individuals (fewer than 30k SNPs overlapping the Legacy 1240k panel), and retaining only the highest-coverage individual among first- and second-degree relatives (five first-degree and four second-degree relationships identified through BREADR v.1.0.1 [43] (Additional file 1: Fig. S2). We merged the final dataset with previously published ancient and modern data (see “Methods”). Using first permanent molars to represent early-life compositions, we conducted strontium isotope analyses on 12 individuals at the Adam Mickiewicz University Isotope Laboratory in Poznań, Poland, while oxygen isotope measurements were performed on eleven of these samples at the Stable Isotope Laboratory of the Centre for Arctic Gas Hydrate, Environment, and Climate at The Arctic University of Norway in Tromsø (see “Methods”; Additional file 1: Note S2; Additional file 2: Data S2).

### Divergent genetic ancestries drive Transtigris Bronze Age shifts from the Pre-Pottery Neolithic

**Fig. 2:**
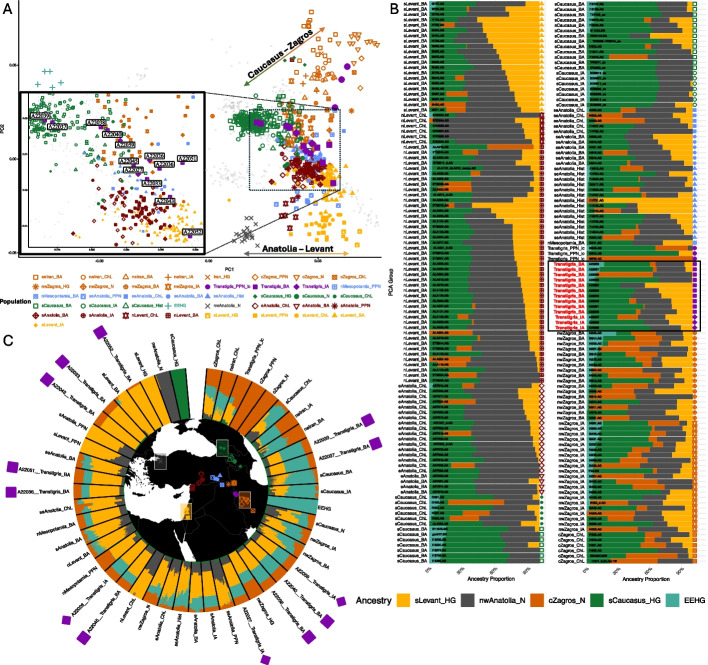
Genetic structure and diversity of Bakr Awa individuals in the context of ancient West Eurasian variation. **A** Principal components analysis (PCA) projecting ancient individuals onto the genetic variation of West Eurasia. The newly reported Bronze Age (BA) and Iron Age (IA) Transtigris samples (purple squares and diamonds, respectively) are highlighted in the zoomed inset. They position intermediate to established ancestry clines such as the Caucasus-Zagros (PC1-PC2 top) and Anatolia-Levant (PC1-PC2 bottom). **B** PANE ancestry estimation across a selection of regional populations using five ancestral reference populations capturing Near Eastern PCA variation. Bakr Awa individuals are highlighted in red bold text and encapsulated in a black box. All population shape-color combinations correspond to panel **A** legend. **C** Fastmixture analysis (K = 5) displayed as a sector plot, with bars organized by dominant ancestry component. Interior map shows geographic midpoints of analyzed population groups with the five fastmixture ancestral sources overlaid by a polygon (EEHG excluded for clarity). The newly reported Bronze Age (BA) and Iron Age (IA) Transtigris samples are indicated with purple squares and diamonds, respectively, after their label

We visualized the genetic structure of the Bakr Awa individuals within the broader context of 747 ancient Eurasians through principal component analysis (PCA). Using *smartpca* [44], we projected ancient individuals onto PCs defined by 1,152 modern Eurasians [45] (Additional file 2: Data S3) based on SNPs intersecting the Human Origins dataset [46] (see “Methods”; Fig. 2A). Importantly, PCA analyses have been demonstrated to be robust to the technical bias of in-solution hybridization capture assays, at least up to the first three PCs [33, 34]. We observe that the resulting genetic structure of the ancient Near East closely mirrors its geography, with a gradient stretching from Anatolia in the west to Iran in the east. Notably, the Bakr Awa individuals, together with previously published northern Mesopotamian and southeastern Anatolian samples, form a distinct cline orthogonal to this main gradient. This secondary cline captures genetic variation between the southern Caucasus in the north and the Levant in the southwestern regions of the Near East.

The easternmost edge of the Mesopotamian PCA cline is captured by the previously published Pre-Pottery Neolithic Bestansur individuals (Transtigris_PPN_lc—labeled accordingly to denote the lower SNP coverage) [4] who cluster with individuals from the Iranian Pre-Pottery Neolithic and Neolithic central Zagros mountain region and Mesolithic Alborz mountains [47]. Despite their geographic proximity (< 33 km), Bakr Awa individuals cluster separately from Pre-Pottery Neolithic Bestansur in PCA, suggesting a genetic shift at Mesopotamia's eastern periphery by the Bronze Age. Outgroup *f*_3_-statistics of form *f*_3_(Mbuti; Transtigris, *Test*) support this pattern, revealing an Anatolian/Levantine/Caucasus-related ancestry gradient (*Test*) that distinguishes Bakr Awa from the ancestral Bestansur/central Zagros-related ancestry core (Additional file 1: Fig. S3; Additional file 2: Data S4). We note that comparing differences in shared drift between Bestansur PPN (Legacy 1240k) and Bakr Awa (Arbor-PP) to *Test* populations (Legacy 1240k) by placing both in the P2 position of the *f*_3_-statistic violates published co-technology guidelines. However, because the observed patterns run inverse to the expected bias, Bakr Awa’s affinity to Anatolian/Levantine/Caucasus ancestry suggests the biological signal is overriding any potential technical artifact. Nonetheless, it remains that the small sample set and low SNP coverage for Bestansur PPN preclude us from making further inferences regarding the nature and timing of the putative ancestry shift.

To quantify ancestry variation underlying the Bakr Awa PCA cline, we performed PANE analysis [48], fixing five distal populations occupying PCA-space boundaries as sources and co-analyzing Bakr Awa individuals alongside Chalcolithic through Iron Age populations from surrounding regions (see “Methods”; Fig. 2B; Additional file 2: Data S5). Eastern European Hunter-Gatherer (EEHG) – a Mesolithic hunter-gatherer meta-population from present-day western Russia who serve as an ancestral proxy for later Pontic-Caspian Steppe-related ancestry [38, 49, 50] (see Additional file 2: Data S6 for included individuals) – and southern Levantine Hunter-Gatherer (sLevant_HG)-related ancestries emerged as primary drivers of genetic structure among Bakr Awa individuals. The highest EEHG proportions occur in Early to Middle Bronze Age (EMBA) individuals A22037 and A22039, who cluster with southern Caucasus populations in PCA, while A22038 (EMBA) and A22040 (Middle Bronze Age, archaeologically ambiguous dating) show slightly lower EEHG ancestry and cluster with individuals from the northwestern Zagros mountains region. The Middle Bronze Age individual A22052, clustering with southern Levantine populations in PCA, possesses one of the largest estimates of sLevant_HG-related ancestry, even among southern Levantine Bronze Age individuals. These ancestry patterns are corroborated by unsupervised fastmixture analysis [51], which clusters A22037/A22039 with individuals from the southern Caucasus, A22038/A22040 with individuals from the northwestern Zagros mountains, and A22052 with individuals from the southern Levant (see “Methods”; Fig. 2C; Additional file 1: Fig. S4).

We sought to identify more temporally recent ancestral contributions driving Bakr Awa variation through qpAdm modeling [38, 46]. Because the qpAdm/Wave algorithm relies on an *f*_4_-statistic matrix to model population relationships, it is theoretically vulnerable to artificial technical affinity between populations in the left (P1, P2) and right (P3, P4) sets. This affinity could cause false inferences of shared drift, leading to erroneous rejections of valid source models. To mitigate this technical bias, we did not co-analyze mixtures of Arbor-PP and Legacy 1240k individuals in our source set or right group set. Rather, we fixed five sources selected for their PCA positioning adjacent to Bakr Awa variation: central Zagros Chalcolithic (cZagros_ChL), southern Caucasus Late Neolithic-Chalcolithic (sCaucasus_LNChL), eastern Anatolia Chalcolithic (eAnatolia_ChL), southern Levantine Pre-Pottery Neolithic (sLevant_PPN), and a metapopulation of Eurasian Steppe Yamnaya-related individuals (YamnayaCluster) previously identified as contributing to Bronze Age southern Caucasus ancestry [20] (see “Methods”; Additional file 1: Fig. S5; Additional file 2: Data S7).

Rather than the elevated sCaucasus_LNChL proportions expected from Neolithic-era migration, Caucasus-PCA outliers A22037 and A22039 possess Caucasus_LNChL ancestry comparable to other Bakr Awa individuals while exhibiting elevated YamnayaCluster ancestry, suggesting their Caucasus-related ancestry postdates the Yamnaya expansion that impacted the southern Caucasus [20, 52, 53]. The Levantine-PCA outlier A22052 displays higher sLevant_PPN-related ancestry as expected from its PCA position, while Middle Bronze Age individual A22043, positioned slightly more distantly but still towards the Levantine PCA cluster, possesses similar sLevant_PPN-related ancestry proportions. More unexpectedly, A22040 and A22038 lacked predominant central Zagros or sCaucasus_LNChL-related ancestry despite clustering with individuals from the northwestern Zagros in PCA, suggesting the existence of an unsampled population(s) in the Zagros mountain region possessing an ancestry intermediate between the Late Neolithic-Chalcolithic central Zagros and southern Caucasus-related ancestry.

The near-absence of long runs of homozygosity (ROH) in our sample – only A22043 exhibits a short ROH (< 10 cM) on chromosome seven (Additional file 1: Fig. S6) – is consistent with the emerging picture that Bakr Awa individuals descend from diverse ancestral origins, as ROH probability decreases with both increasing population size and parent–offspring dispersal, given that ancestry disperses geographically back in time [54] (Additional file 1: Fig. S6).

### Spatiotemporal variation in genetic connectivity between Bakr Awa Bronze Age and its neighbors

To achieve finer ancestry spatiotemporal resolution beyond the broad patterns established above, we conducted pairwise qpWave analysis to identify ancient individuals who form a genetic clade with each Bakr Awa individual (*p*-value ≥ 0.05 after Benjamini–Hochberg correction) relative to a diverse set of outgroups (see “Methods”; Additional file 1: Fig. S7; Additional file 2: Data S8). Across the Near East and through time, we observe several qpWave connections linking Bakr Awa and other ancient individuals as a genetic clade. We thus sought to test whether each Bakr Awa individual’s set of qpWave connections possessed a significant enrichment in geographical regions and time periods. To do so, we implemented the following grouping framework, incorporating both spatial and temporal dimensions. Individuals were assigned to six macro-regions: (1) Caucasus, (2) Zagros, (3) southeastern Anatolia and northern Mesopotamia, (4) northern Levant, southern and eastern Anatolia, (5) southern Levant, and (6) central, southwestern, and northern Anatolia. These macro-regions were further stratified into three temporal periods (TPs:1–3) defined roughly relative to the Bakr Awa Bronze Age: (3.4–1.2] ka BP (younger, TP1), [4.2–3.4] ka BP (contemporaneous, TP2), and (7.7–4.2) ka BP (older, TP3).

**Fig. 3:**
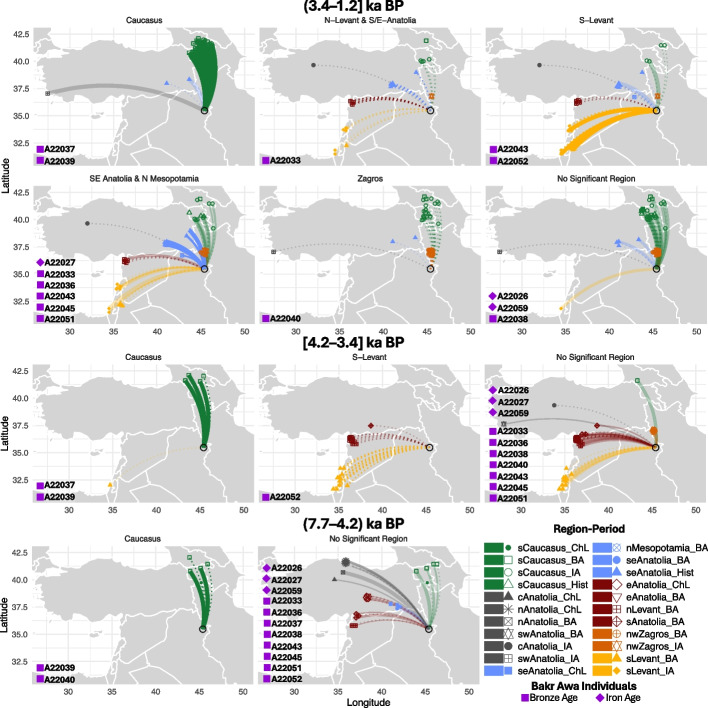
Spatiotemporal genetic affinities of the Bakr Awa individuals. Geographic display of regions where Bakr Awa individuals exhibit a significant enrichment in individual-wise qpWave clades (see “Methods”). Bakr Awa individuals are indicated with a corresponding sample label and chronology shape/color designation. Bold lines denote shared connections amongst Bakr Awa individuals to ancient individuals from enriched regions; transparent lines indicate shared connections to individuals from other regions; dashed lines represent unique qpWave pairings. Shape-color patterns correspond to Fig. 2 regional-period groups

To test for significant enrichment in macro-region qpWave clade relationships, we employed two complementary statistical approaches. The first was a permutation test generating a one-sided *p*-value (see “Methods”). However, because we observed significant differences in the distribution of individual-based pairwise 1-outgroup *f*_3_-statistic tests between the six macro-regions (see “Methods”; Additional file 1: Fig. S8; Additional file 2: Data S9), indicative of differing levels of genetic homogeneity across groupings, we additionally performed a Bayesian Generalized Linear Mixed Model (BGLMM) analysis incorporating outgroup *f*_3_-statistic tests as a random variable to account for the background population relatedness (see “Methods”; Additional file 1: Fig. S9; Additional file 2: Data S10). The BGLMM approach proved to be more conservative, yielding no unique significant results while sharing 47% of the total significant associations with the permutation method (Additional file 1: Fig. S10). When distributed across time periods, however, this pattern shifts during the temporal period TP1 (3.4–1.2 ka BP), where shared signatures exceed those found solely by the permutation approach (Additional file 1: Fig. S10). As such, we employed an integrated interpretive heuristic where only results significant for both tests were considered (Fig. 3; Additional file 2: Data S11; Additional file 1: Fig. S10), with one exception – Bakr Awa individuals possessing significant qpWave clade tests exclusively from a single macro-region, which were excluded from the BGLMM analysis, are treated as identifying significant genetic associations with said macro-region.

Despite no statistically significant BGLMM enrichment with any macro-region during the oldest time-period bin (7.7–4.2) ka BP, qualitative patterns of multiple qpWave clade links to individuals across the northern and northwestern Near East, in addition to a uniform rejection of clade formation between Bakr Awa and Zagros mountain individuals, further support the northern and northwestern Near East as the most likely source of the ancestry, shifting the Transtigris genetic landscape between Bakr Awa and the Pre-Pottery Neolithic Bestansur. The Bakr Awa qpWave connections to south eastern Anatolia and northern Mesopotamia intensify during the youngest time-period bin (3.4–1.2] ka BP, which yields the largest number of individuals included in a significant regional enrichment of qpWave clades across all periods and macro-regions (five Bronze Age, one Iron Age). The absence of ancient genomic data from SE Anatolia-N Mesopotamia for the contemporaneous (3.4–1.2] ka BP window currently precludes establishing when this enrichment in south eastern Anatolian and northern Mesopotamian connections first emerged, underscoring the importance of new data from across Mesopotamia to elucidate the formation of the Bronze Age Near Eastern genetic landscape.

Our qpWave enrichment analysis also captures Levantine-related ancestry affinities identified above. During the roughly contemporaneous [4.2–3.4] ka BP period, the Middle Bronze Age Levantine-PCA outlier A22052 is the only sample to show significant enrichment in qpWave cladal pairs with southern Levantine individuals. Then, in the youngest time-period bin (3.4–1.2] ka BP, both A22052 and Bronze Age individual A22043 – the latter slightly less shifted towards the Levantine cluster in PC space – possess significant connections in qpWave regional enrichment with the southern Levant. However, of the two, only A22043 also displays a significant connection in qpWave regional enrichment with individuals also from the SE Anatolia-N Mesopotamia macro-region, consistent with their less-than-extreme outlier position with respect to Levantine-related ancestry as described above.

Consistent with PCA/PANE and fastmixture results, the Early to Middle Bronze Age Caucasus-PCA outliers (A22037 and A22039) possess an enrichment in significant qpWave clade tests with the Caucasus macro-region in the younger (3.4–1.2] ka BP and roughly contemporaneous [4.2–3.4] ka BP periods. However, in the oldest time-period bin (7.7–4.2) ka BP, only individuals A22039 and A22040 – the latter displaying PCA affinity with individuals from the northwestern Zagros – possess significant qpWave test clade relationships exclusive with individuals from the Caucasus region. Notably, we observe no significant enrichment in qpWave Bakr Awa pairing between southern Caucasus individuals from Kura-Araxes versus non-Kura-Araxes cultural contexts (5.41–4.75 ka BP) (Fisher's exact test *p*-values ≥ 0.5), suggesting connections to the Caucasus region transcend these cultural boundaries (Additional file 1: Fig. S11).

### Y-chromosome and stable isotope signatures of mobility

Initially appearing in Areni1 in the mid-late 5th millennium BCE (6.450–5.951 ka BP) [47], the EEHG-related component of Caucasus ancestry disappeared in the early southern Caucasian Kura-Araxes culture, only to reappear in the mid-third millennium BCE with the expansion of Yamnaya-related individuals from the Eurasian steppes [20, 38, 53]. In addition, the Y-chromosome R-Z2103 (R1b) haplogroup has been identified as a genetic marker linking Yamnaya-related populations in Armenia to those in the northwestern Zagros, including Hasanlu [20, 38, 52]. Consistent with this pattern, our qpAdm analysis showed YamnayaCluster ancestry was highest among post-Kura-Araxes southern Caucasus Bronze Age and northwestern Zagros Bronze and Iron Age individuals (Additional file 1: Fig. S5). Notably, of the two Bakr Awa Caucasus-PCA outliers, A22037 possesses YamnayaCluster ancestry at the upper end of the southern Caucasus range, while A22039 falls in the middle. Moreover, A22037 – the only male among the EMBA outliers, dating to c. 2112–1800 BCE – also carries the R-Z2103 (R1b) Y-chromosome haplogroup (Additional file 2: Data S1), providing additional evidence for his connection to Yamnaya-related Caucasus ancestry.

**Fig. 4:**
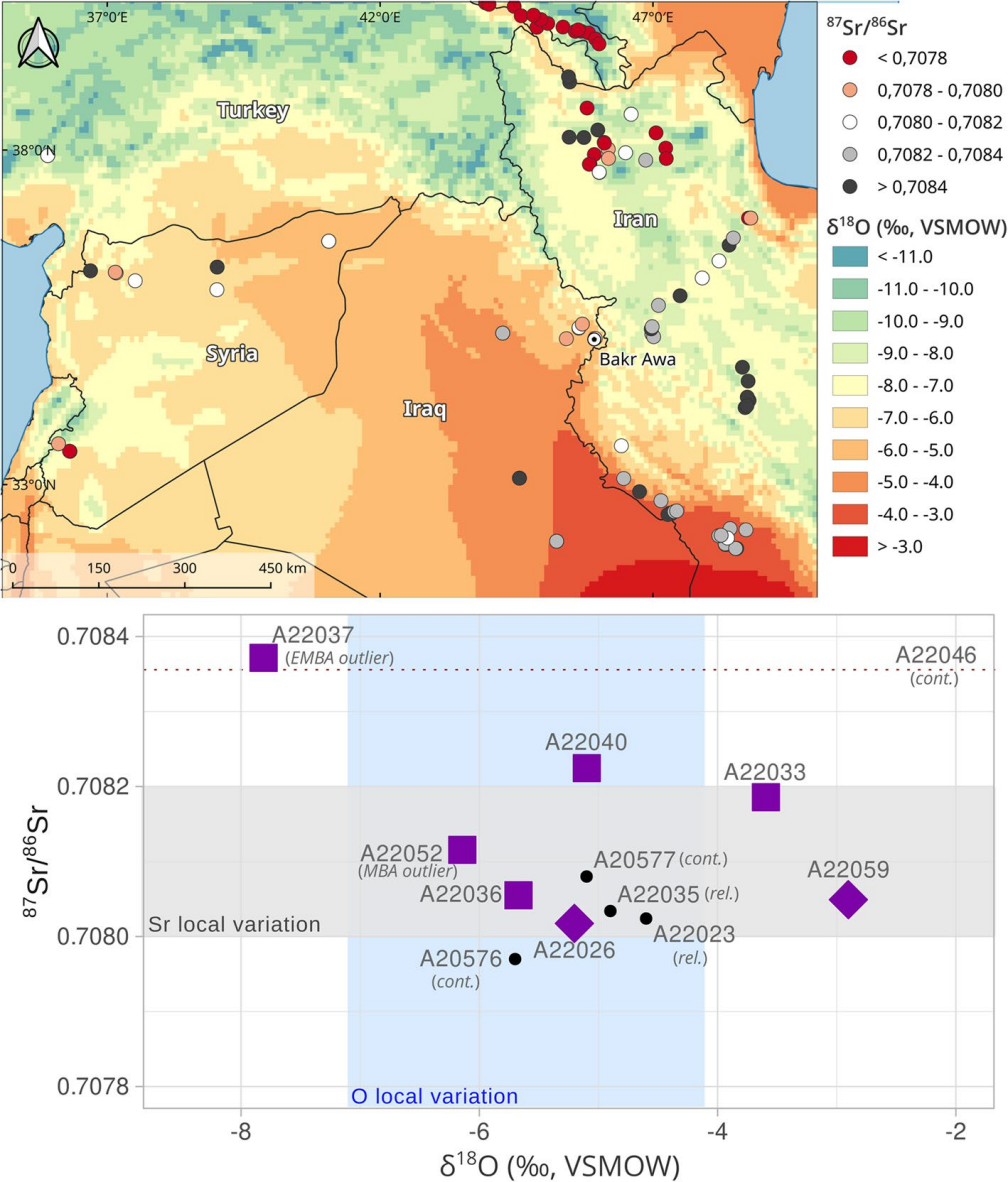
Stable isotope evidence for local and non-local origins. δ^18^O and ^87^Sr/^86^Sr values plotted against proposed local ranges (shaded regions). Departures from local signatures may indicate migration. Dotted line shows ^87^Sr/^86^Sr for A22046 (no δ^18^O data). Labels indicate chronology shape/color; "cont." = sample generated DNA but was genetically contaminated, "rel." = a related individual excluded from population analyses, individuals included in population analyses are indicated with purple squares and diamonds for the Bronze and Iron Age, respectively

To investigate potential non-local origins of the Bakr Awa individuals, we analyzed stable isotopes (δ^18^O and ^87^Sr/^86^Sr) from their first permanent molars representing early-life (Fig. 4). Whilst stable isotope analysis can provide complementary evidence for individual mobility, inherent methodological limitations warrant cautious interpretation. The relatively uniform distribution of bioavailable strontium across Southwest Asia prevents unique geographic assignments, and drinking water sources influence δ^18^O values – potentially misleading origin assignment [55]. While ^87^Sr/^86^Sr ratios alone suggested ~ 33% (4/12) of the tested individuals may have non-local origins, the more stringent dual-isotope approach identified only one individual, the Caucasus-PCA outlier A22037, whose dual isotopic signature is most consistent with an origin in the Iranian Zagros mountains east of Bakr Awa. However, A22037's genetic profile creates an apparent paradox – noted above is that the qpWave analysis reveals he possesses no significant spatiotemporal enrichment in connections with individuals from the Zagros mountains, but rather a persistent southern Caucasus affinity. Moreover, only a single Bakr Awa individual possessed a regional enrichment in qpWave clades with individuals from the Zagros region (A22040 during the younger (1.2–3.4] ka BP period), on the surface suggesting a lack of connections between the Transtigris and Zagros region during the Bronze Age. We propose that this discrepancy is likely caused by incomplete ancient DNA sampling of the northwestern Zagros mountain region from Chalcolithic to the Middle Bronze Age, currently represented by only two Middle Bronze Age individuals from Hasanlu and Hajji Firuz. As such, additional Chalcolithic to Bronze Age ancient genomes from the Zagros and eastern Anatolia will be essential for elucidating the relationship between Caucasus-related ancestry at Bakr Awa and potential intermediary populations.

### Ancestry integration characterizes the Bronze-to-Iron Age transition at Bakr Awa

Following a catastrophic fire and site abandonment in the Late Bronze Age, Bakr Awa was reoccupied during the Iron Age – a period characterized by documented population movements throughout the ancient Near East. To determine whether these events resulted in genetic ancestry shifts, we analyzed three Iron Age individuals: two dated to the Neo-Assyrian period (911–609 BCE; A22026 and A22059) and one to the Achaemenid period (550–331 BCE; A22027) against the antecedent Bakr Awa Bronze Age structure. PCA/PANE and fastmixture analyses provide preliminary indications that the genetic profiles of Iron Age individuals at Bakr Awa fall within, rather than deviate from, the diversity of the preceding Bronze Age (Fig. 2A-C). Further evidence against a major ancestry shift between the Bronze and Iron Ages comes from the qpWave spatiotemporal enrichment analysis. Consistent with most Bronze Age individuals, Iron Age individuals exhibit no significant individual-based qpWave clade enrichment with geographical regions from the two oldest time periods.

**Fig. 5:**
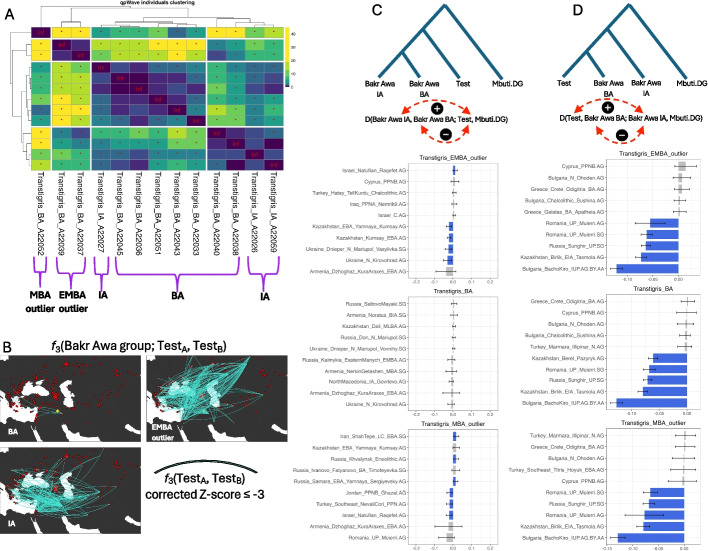
Genetic continuity and discontinuity between Bronze Age and Iron Age populations at Bakr Awa. **A** Hierarchical clustering of qpWave − log10(*p*-values); stars indicate qpWave test *p*-value ≤ 0.01. **B** Admixture *f*_3_-statistics of form *f*_3_(Bakr Awa group; *Test*_A_, *Test*_B_) with significant population pairs (adjusted Z-scores) connected by turquoise lines. **C** Symmetry D-statistics [D(Bakr Awa IA, BA; *Test*, Mbuti)] testing cladality; colored bars indicate |Z|> 3. **D** Affinity D-statistics [D(*Test*, BA; IA, Mbuti)] showing Bakr Awa Iron Age shares more drift with Bakr Awa Bronze Age groups (negative values)

To facilitate a group-level contrast of Bronze and Iron Age ancestry, we formed four Bakr Awa meta-analysis groups (three Bronze Age and one Iron Age) through hierarchical clustering on individual pairwise qpWave − log₁₀-transformed *p*-values [56–58] (see “Methods”; Fig. 5A; Additional file 2: Data S12). Our clustering separates the Levantine-PCA Bronze Age outlier (A22052) first, followed by the two Caucasus-PCA Bronze Age outliers (A22037, A22039). Among the Iron Age individuals, the individual dating to the Achaemenid period (A22027) clusters with five Bronze Age individuals, while the individuals dating to the Neo-Assyrian period (A22026, A22059) cluster with Bronze Age individuals A22038 and A22040. Although the individuals dating to the Neo-Assyrian and Achaemenid periods do not form a qpWave clade (*p* ≤ 0.01), we merged all three into a single Iron Age group to increase statistical power. Similarly, we retained the Bronze Age Levantine-PCA and Caucasus-PCA outliers as distinct clusters and merged all other Bronze Age individuals into a single meta group.

We first tested for population continuity between each of the Bakr Awa Bronze and Iron Age meta-groups using symmetry D-statistics of form D(Bakr Awa Iron Age, Bakr Awa Bronze Age; *Test*, Mbuti.DG), across 343 Eurasian *Test* groups, restricting the P1 and P2 positions solely to the Bakr Awa Arbor-PP in accordance with published guidelines (see “Methods”; Fig. 5C; Additional file 2: Data S13). Tests rejected strict continuity between the Iron Age and both Bronze Age outlier groups – the Iron Age showed excess Levantine-related ancestry relative to Caucasus-PCA outliers and excess Steppe-related ancestry relative to the Levantine-PCA outlier – but revealed no discontinuity with the non-outlier Bronze Age group. This pattern is also observed in qpAdm modeling, whereby we tested whether the Bakr Awa Iron Age could derive from one, two, or all three Bakr Awa Bronze Age source groups (see “Methods”; Additional file 2: Data S14). This implementation of qpAdm strictly avoids the mixture of Arbor-PP and Legacy 1240k data in either the left- or right-group positions. Here, only the non-outlier Bakr Awa BA group was plausible as a single source (*p*-value = 0.16), with multi-source models estimating it as the primary ancestry contributor (Transtigris_BA + Transtigris_EMBA_outlier two-source: 86 ± 5% and *p*-value = 0.50; three-source: 65 ± 16% and *p*-value = 0.67).

To determine if the Bakr Awa Iron Age shared more drift with the Bronze Age meta-groups than any other *Test* population, we used affinity D-statistics of form D(*Test*, Bakr Awa Bronze Age group; Bakr Awa Iron Age, Mbuti.DG). Whilst this configuration violates published guidelines, we re-ran the analysis with a separate SNP set designed for the co-analysis of mixed technology-generated ancient DNA data [39] and note a strong correlation between the D-statistic estimates and Z-scores computed from the two different SNP sets (Lin’s Concordance Correlation Coefficient = 0.97 and 0.94, respectively; Additional file 1: Fig. S12). The results revealed that no *Test* population shares significantly more drift with Bakr Awa Iron Age than the Transtigris Bronze Age meta-groups (see “Methods”; Fig. 5D; Additional file 2: Data S15).

We then ran admixture *f*_3_-statistics of form *f*_3_(Bakr Awa; *Test*_*A*_, *Test*_*B*_) – adjusting the Z-scores to account for multiple hypothesis testing – to evaluate whether the Bakr Awa analysis meta-groups exhibit qualitatively different patterns regarding the source populations identified in statistically significant formal admixture tests (see “Methods”; Fig. 5B; Additional file 2: Data S16). This *f*_3_-statistic configuration conforms to published guidelines, as the Arbor-PP Bakr Awa data exhibit no technical bias toward the P2 and P3 populations. Furthermore, although Arbor-PP and shotgun data share comparably low GC (allelic) bias [33], this has not been shown to generate artificial shared drift in *f*-statistics [33, 34]. Here, we observe a clear qualitative contrast between the Bronze and Iron Age. The EMBA outlier group predominantly includes Eurasian Steppe populations, whereas the Iron Age group, which also includes Eurasian Steppe populations, also possesses many southern Levantine populations among significant (adjusted Z-score ≤ −3) *f*_3_-test pairs. This result appears consistent with the symmetry D-statistic test’s rejection of strict population continuity between the Bakr Awa EMBA outlier and IA meta-groups, whilst supporting a model of ancestry integration, as the Bakr Awa IA and EMBA outlier groups share evidence of Steppe-related admixture. Interestingly, both meta-groups frequently include populations from the central Zagros mountain region in their significant *f*_3_-statistic tests, which may be functioning as a proxy for the distal Transtigris ancestry represented by Bestansur PPN. Notably, the non-outlier Bakr Awa BA group produced only four significantly negative *f*_3_-statistics, all including central Zagros Neolithic populations.

## Discussion

Despite its importance as a conduit between the Mesopotamian plains and the Zagros mountains, the Transtigris has been understudied in archaeogenetic research. In the present study, we report novel genome-wide ancient DNA (*n* = 17) and stable isotope data (*n* = 12) from Bronze and Iron Age individuals excavated at Bakr Awa, a prominent archaeological mound in the Transtigris region [59, 60]. By contextualizing our findings with published ancient genomes from across the ancient Near East and Eurasia, we uncovered the genetic ancestry of the Bronze Age Transtigris and tracked the population dynamics accompanying the transition to the Iron Age.

Previous research had demonstrated that during the Pre-Pottery Neolithic, the Transtigris ancestry possessed a close affinity to the ancestry observed in Neolithic central Zagros Mountains inhabitants [4]. Interestingly, we have been able to demonstrate that by the Bronze Age, this localized signature underwent a fundamental shift whereby the inhabitants of Bakr Awa display a diverse, heterogeneous ancestry with genetic affinities towards Pre-Pottery Neolithic and Neolithic populations in the Levant, Anatolia, northern Mesopotamia, and the southern Caucasus.

The influence of geographically diverse ancestries on the Bronze Age Bakr Awa settlement inhabitants parallels the archaeological evidence of shifting cultural influences from southern Mesopotamia (Ur III period), northern Mesopotamia (Akkadian period), and Hurrian and western Iranian traditions (Middle Bronze Age) [61]. Notably, ceramic and textual evidence confirm Hurrians among the predominant ethnolinguistic groups at Bakr Awa. However, our small sample size and limited regional comparative data necessitate caution in our interpretations. Notwithstanding these limitations, the appearance of Yamnaya-related southern Caucasus ancestry in two individuals (A22037, A22039) dating to approximately 2000 BCE – and to a lesser degree A22038 and A22040 – coincides with expanding Hurrian influence after the Gutian overthrow of the Akkadian empire (ca. 2150 BCE). One of those individuals – the adolescent male A22037 – also carries the Yamnaya-associated R-Z2103 (R1b) Y-chromosome haplogroup (A22039 is female), which is at high frequency in males from the northwestern Zagros Iron Age site of Hasanlu [52, 62] (10 of the 14 males for which a Y chromosome haplogroup can be assigned are R1b).

Previous research has identified mobility connections between Hasanlu and the southern Caucasus, evidenced by shared European hunter-gatherer (EHG) ancestry and a high frequency of the R1b Y-chromosome haplogroup [52]. Notably, when utilizing a conservative dual-stable-isotope approach, we identified the most parsimonious recent origin of A22037 as east of Bakr Awa in the Zagros mountains. However, the relatively uniform distribution of bioavailable strontium isotope ratios across Southwest Asia and the influence of drinking water on δ^18^O values assign some degree of uncertainty to this assignment. Moreover, as this is a single observation, it does not indicate large-scale migration between the Zagros and Transtigirs; however, it offers some evidence of an unknown degree of Zagros-Transtigris connectivity, potentially explaining the ancestry links between the southern Caucasus and Bakr Awa.

Despite limited data, previous research indicates that Iron Age individuals from the Urartian Kingdom's core regions near Lake Van lacked Eastern European hunter-gatherer (EHG) ancestry [52]; though, a notable female outlier from Çavuştepe (761–479 calBCE) deviates from this pattern by possessing a large fraction of Yamnaya-related ancestry (also see Additional file 1: Fig. S5). Rather, the authors propose that individuals within the Hurro-Urartian language family may actually be associated with greater Levantine-related ancestry [52]. While our multidisciplinary results (genetic, isotopic, archaeological, and textual) provide additional data surrounding the question of the nature of the Hurrian expansion into eastern Mesopotamia, the underlying demographic model connecting the southern Caucasus, eastern Anatolia, and the Zagros with Mesopotamia is undoubtedly complex. To fully elucidate this model and integrate it within the broader archaeological and historical context, future research will require extensive sampling across Bronze and Iron Age populations in Mesopotamia, eastern Anatolia, and the Zagros mountains.

Archaeological evidence indicates that Bakr Awa's prosperous Bronze Age occupation terminated in a catastrophic fire during the 15th–14th century BCE, predating the broader regional Late Bronze Age collapse (ca. 1200 BCE) by several centuries, with the site remaining abandoned until reoccupation in the Iron Age. Interestingly, we observe no evidence of major ancestry shifts accompanying this hiatus and reject a model of complete population replacement. Rather, Iron Age ancestry at Bakr Awa appears to represent an integration of the preceding Bronze Age genetic structure possessing ancestral components from each of the outlier Bronze Age meta-groups.

Despite the Shahrizor Plain's presumed incorporation into the Neo-Assyrian province of Zamua, Bakr Awa's Iron Age pottery shows Urartian and Median rather than Neo-Assyrian affinities – a pattern potentially mirrored in our genetic findings. The Neo-Assyrian period dated individuals lack a significant regional enrichment of qpWave clade relationships across all time periods, suggesting diverse rather than region-specific ancestral connections. In contrast, the Achaemenid period individual shows a significant enrichment in genetic clade relationships with roughly contemporaneous individuals from southeastern Anatolia-northern Mesopotamia. Though preliminary, these patterns are suggestive that the Achaemenid transition may have involved a subtle ancestry shift toward northern Mesopotamian-related ancestry at Bakr Awa; however, expanded sampling across the entire Mesopotamian region and larger sample sizes for the Bakr Awa Iron Age will be necessary for validation.

The integration of divergent ancestries observed in the Iron Age community at Bakr Awa following the Bronze Age hiatus reflects a broader emerging picture of ancient Near Eastern archaeogenetics during this period – namely the lack of major ancestry shifts, such as was previously observed during the Chalcolithic period [63, 64], despite being a time of higher individual mobility. While sample sizes remain limited and regional sampling sparse, the observation of decreasing–or stabilizing [56]–inter-regional *F*_ST_ from the Bronze and into the Iron Age with a simultaneous increase in inter-regional genetic distance (measured as 1—*f*_3_), appears to support a proposed "expanding mobility model" whereby regionally unique distant gene flow drives regional divergence [64]. Synthesizing this framework with the proposed "transient mobility" hypothesis – where non-reproductive migrants lead to the preservation of local geographic genetic structures [56]–provides a possible explanatory model for the temporal population structure stability. Under these hypotheses, future sampling should expect to uncover in regions of high mobility an increase in “ancestry outliers” similar to what has been observed in the Mediterranean [56, 58]. Ultimately, refining our understanding of the impacts of Iron Age mobility on the genetic landscape and structure of the eastern Mediterranean and ancient Near East will require larger sample sizes and, importantly, broader site representation.

Throughout this manuscript, we have implicitly assumed that shifts in genetic profiles reliably track population movements [65] and that the identified source ancestries are broadly representative of their periods and locales, thereby allowing inferences of the spatiotemporal homeland of ancestry shifts in the Transtigris. However, several methodological limitations could challenge these premises. In particular, the combination of small sample cohorts and population structure could result in undetected local ancestry in an older population that, when not accounted for, could create a false signal of migration-driven ancestry shifts in the younger population. Our inference of an ancestry shift between the Pre-Pottery Neolithic and the Bronze Age in the Transtigris is susceptible to this bias, given the low coverage and small sample size at Bestansur. Nevertheless, our conclusion that a major ancestry shift occurred between the Pre-Pottery Neolithic and the Bronze Age is consistent with the emerging evidence of large-scale demographic changes during the Chalcolithic and Early Bronze Age [64] across both the northern ancient Near East [63], central and northwestern Zagros [47, 66], and the southern Levant [47, 67].

Cognizant of the technical biases that arise from the co-analysis of data generated across different in-solution hybridization capture assays (such as the Arbor Prime Plus kit), we followed published guidelines in configuring our population genetic analyses to limit the induction of artificial signatures of shared genetic drift between samples generated from the same technology. Where specific tests necessitated deviation from the published guidelines we either demonstrated that the observed biological signals ran inverse to the expected bias (for example, when using the outgroup *f*_3_-statistic to identify differences in regional shared drift between the Bestansur PPN and Bakr Awa groups), or we verified our findings using the conservatively filtered "Compatibility" SNP panel (such as the D-statistic affinity between the Bakr Awa Bronze and Iron Age groups).

## Conclusions

In conclusion, our manuscript's primary findings appear to be driven by genuine biological population history and are consistent with Mesopotamia's role as a "melting pot" of ancient Near Eastern ethnolinguistic groups – complementing archaeological and textual reconstructions; namely, (1) an ancestry shift between the Pre-Pottery Neolithic and Bronze Age characterized by the deviation from a Neolithic central Zagros-related ancestry towards a northern Near Eastern ancestry profile possessing Anatolian-northern Mesopotamian and subtly Levantine-related signatures; (2) the presence of population structure in the Bronze Age driven by divergent ancestries such as excess southern Levantine-related and Caucasus Yamnaya-related ancestry; and (3) the weakening of Bronze Age population structure and integration of the divergent ancestries in the Iron Age community following site abandonment and re-occupation – a pattern suggestive of a shift in societal dynamics accompanying the emergence of the Iron Age.

## Methods

### Laboratory methods

#### Stable-isotope analysis

For the strontium and oxygen isotope analysis, the first permanent molars of 12 individuals were selected (Additional file 1: Note S2; Additional file 2: Data S2). To establish the levels of bioavailable strontium ratio, samples of four modern plants were collected in the area of Shahrizor Plain and the neighbouring hill [68]. The strontium isotope analyses were carried out in the Isotope Laboratory of the Adam Mickiewicz University at Poznań, Poland. An isolated sample was cleaned using an ultrasonic bath in ultra-pure water. Then, ca. 10 mg of powder was treated five times with ultrapure acetic acid [69]. Next, samples were dissolved in HNO_3_ and kept overnight in PFA vials on a 100 °C hot plate. The Sr separation was achieved using a miniaturized chromatographic technique [70]. A strontium was loaded with a TaCl_5_ activator on a single Re filament and analysed in dynamic collection mode on a Finnigan MAT 261 mass spectrometer. The measured NBS 987 Sr standard yielded ^87^Sr/^86^Sr = 0.710238 ± 10 [71]. The ^87^Sr/^86^Sr values were corrected to ^86^Sr/^88^Sr = 0.1194, and then normalized to the certified value of NIST-987 = 0.710240.

Whenever possible, samples of powdered enamel carbonate were measured for oxygen isotope ratio in the Stable Isotope Laboratory at the Centre for Arctic Gas Hydrate, Environment, and Climate at The Arctic University of Norway, Tromsø, Norway. Each sample was placed in 4.5 mL vials and flushed with He, and five drops of water-free H_3_PO_4_ were added manually with a syringe. After equilibration for > 3 h at T = 50 °C, the samples were analyzed on Gasbench II and MAT253 IRMS. Measurements were normalized to VPDB by three in-house standards (Isolab A: δ^13^C_VPDB_ = 1.96 ± 0.05‰, δ^18^O_VPDB_ = −2.15 ± 0.05‰; Isolab B: δ^13^C_VPDB_ = −10.21 ± 0.05‰, δ^18^O_VPDB_ = −18.59 ± 0.04‰; Merck CaCO_3_: δ^13^C_VPDB_ = −48.95‰, δ^18^O_VPDB_ = −13.98‰), which were normalized to international standards: NBS18, NBS19, LSVEC. Oxygen stable isotopes were calculated in retaliation to the Vienna PeeDee Belemite standard and expressed in per mill (‰). Further, δ^18^O_VPDB_ were calculated to δ^18^O_SMOW_ according to a formula proposed by Coplen et al. 1983 [72], and then into drinking water values following a formula proposed by [73], allowing for direct comparison between obtained results and annual mean ^18^O values of local precipitation [74].

#### Ancient DNA extraction and library preparation

All sample preparation, DNA extraction, and library preparation steps were performed in the purpose-built ancient DNA laboratory at the Australian Centre for Ancient DNA (ACAD), Adelaide University, Australia. Sample pretreatment consisted of ultra-violet (UV) light treatment for 30-min on each side [75] to minimise surface contamination. When sampling the petrous bones, we preferentially selected the dense region within the otic capsule as it has been shown to have an average higher concentration of endogenous DNA in samples from arid conditions [76, 77]. Tooth material was taken from the cementum layer of the tooth root after the internal pulp and dentine were removed following protocols outlined in Higgins et al. 2013 and Damgaard et al. 2015 [78, 79]. We ground ~ 50–100 mg of petrous and tooth material into a coarse powder using a stainless-steel mortar, pestle, and sleeve mechanism similar to [80]. During sample processing, a blank control was included for each set of seven samples to monitor contamination. Following the sampling and pulverisation of teeth and petrous bones, we predigested the sample powder in 1 mL of decalcifying agent 0.5 M Ethylenediaminetetraacetic (EDTA) (pH 8.0) (Life Technologies, Carlsbad, CA, USA) for one hour at 37ºC under constant rotation [79]. The EDTA supernatant was removed by pipetting following centrifugation. The powder then underwent overnight (~ 8 h) digestion in a lysis buffer consisting of 970 mL of 0.5 M EDTA and 30 μL of Proteinase-K 20 mg/mL (Life Technologies) under constant rotation at 55ºC. Isolation and separation of the DNA from the supernatant was performed through medium-sized silica adsorption [81] in a modified phosphate-binding buffer (PB) [13.6 mL PB buffer (Qiagen, Valencia, CA, USA), 7 µl Tween-20 (Sigma-Aldrich) and 420 µl 3 M sodium acetate (~ pH 5) (Sigma-Aldrich, Saint Louis, MO, USA)] and rotated at room temperature for one hour. The silicon dioxide particles were then purified in 80% ethanol, and the DNA was eluted with 100 µL of TLE buffer [10 mM Tris, 0.1 mM EDTA, pH 8.0]. We constructed double-stranded DNA libraries [32] that were partially UDG-treated to restrict cytosine deamination to the terminal nucleotides [31]. In addition, 7-mer internal barcode sequences in both truncated P5 and P7 adapters were added following Llamas et al. 2016 [82] to allow sample identification and exclusion of downstream contamination.

#### DNA enrichment and high-throughput sequencing

Before enrichment, successfully amplified libraries were overamplified to reach the minimum required DNA input of 1000 ng per sample. For each library, the PCR reaction mix consisted of 5–10 µl of library, 25 µl of KAPA HiFi HotStart ReadyMix (Roche), 5 µl each of 10 µM IS5 and IS6 primers [83], and ultrapure water in a total volume of 50 µl. PCR amplification was performed with an initial denaturation and polymerase activation at 98 °C for 2 min, 15 cycles of 98 °C for 20 s, 56 °C for 30 s, 72 °C for 45 s, and a final extension at 72 °C for 5 min. DNA purification was performed using 1.2 × AmpureXP beads with two 80% ethanol washes. We enriched the ancient DNA libraries with the Daicel Arbor Biosciences “myBaits Expert Human Affinities Prime Plus” in-solution enrichment reagents that target the core panel of 1.24 million SNPs along with the mitochondrial genome and an additional set of 46,218 Y chromosome SNPs [33, 34] (https://arborbiosci.com/genomics/targeted-155sequencing/mybaits/mybaits-expert/mybaits-expert-human-affinities). We followed the myBaits protocol of two enrichment rounds with hybridisation temperature at 70 °C and 20 amplification cycles in the first enrichment followed by 10 cycles in the second round. The post- enrichment PCR amplification was performed using KAPA HiFi HotStart ReadyMix (Roche) and IS5 and IS6 primers as described above, with a 98 °C initialisation for 24 s, 7 cycles of 98 °C for 15 s, 60 °C for 30 s, 72 °C for 30 s, and a 72 °C final extension for 60 s. For a subset of libraries that required a reconditioning PCR to reduce heteroduplexes, they were concentrated down to 5 µl and mixed with 10 µl of Herculase Buffer (Agilent), 5 µl of 2.5 nM dNTPs, 1 U of Herculase II Fusion (Agilent), 1 µl each of 10 µM IS5 and IS6 primers, and ultrapure water in a final volume of 50 µl. This solution was then reconditioned with one cycle of 95 °C for 2 min, 58 °C for 2 min, and 72 °C for 5 min. Successfully captured libraries were pooled at equimolar concentration and sequenced on a NovaSeq 6000 platform at the Kinghorn Centre for Clinical Genomics (Sydney, NSW, Australia).

### Computational methods

#### Bioinformatic processing

High-throughput sequence data were processed following commonly used aDNA practices. Sequence reads were deconvoluted using barcodes (I7) and converted from BCL to fastq using BCL2FASTQ conversion software version v2.19.1.403 (support.illumina.com/downloads/bcl2fastq_conversion_software.html). Demultiplexed reads were then processed using Nextflow v21.03.0.edge build: 5518, and nf-core/eager v2.4.1 [84] (a full list of software versions and the eager pipeline report can be found in Additional file 2: Data S17). Sequence reads were mapped to the GRCh37d5 reference genome using bwa-aln with parameters --n 0.01 --k 2 --l 1024 [85]. Bam files were filtered for quality (mapping quality ≥ q25 and base quality ≥ Q30), and a minimum read length of 30 bp. Two nucleotides were trimmed from the terminal ends of all retained reads using the trimBam function of bamUtil (https://github.com/statgen/bamUtil). We called pseudohaploid genotypes with pileupCaller (https://github.com/stschiff/sequenceTools) on the Legacy 1240 k SNPs. Genetic sex determination was performed using SexDetERRmine (https://github.com/nf-core/modules/tree/master/modules/nf-core/sexdeterrmine) with default quality cut-off values for -q30 and -Q30. Authentication of ancient DNA was performed using DamageProfiler [86] to analyze fragment size distribution and post-mortem damage patterns at read termini. Contamination assessment was conducted using mitoverse HaploCheck v1.3.2 [87] for mitochondrial DNA and ANGSD [40] for nuclear DNA in male specimens.

#### Y-chromosome analysis

Y-chromosome haplogroups were assigned using yhaplo v.1.1.2 [88] software, analyzing 20,903 ISOGG consortium SNP positions. Quality control measures resulted in 17,078 retained SNPs after correcting 62 positions and excluding 3,824 SNPs that failed to meet quality thresholds, including removal of provisional, multiallelic, and technically compromised markers.

#### Kinship analysis

To estimate kinship, we used BREADR 1.0.2 [43] using the default settings to calculate the pairwise mismatch rate and to estimate genetic relatedness between pairs of individuals across the Bakr Awa cohort. We identified five first-degree and four second-degree kinships (Additional file 1: Fig. S2). A detailed analysis of the Bakr Awa kinship structure and burial context will be provided in a forthcoming study.

### Population genetic analyses

#### Dataset

The newly generated ancient data were merged with published ancient genomes compiled in the Allen Ancient DNA Resource (AADR) v.62.0 [3, 4, 20, 37, 47, 49, 50, 52, 53, 56, 62–64, 66, 67, 89–104] through the Eigensoft v.7.2.0 mergeit function [44, 105]. A detailed list of individuals, along with their grouping label for each population genetic analysis, is provided in Additional file 2: Data S6.

#### Principal components analysis

For our principal component analysis (PCA), we merged our sequenced data with the publicly available AADR Affymetrix Axiom Genome-Wide Human Origins 1 array (“HO” AADR v.62.0) dataset. Using the smartpca function in EIGENSOFT v.7.2.0 [44, 105], we projected a subset of ancient west Eurasian populations onto variation formed from 70 global modern populations [45] using the parameter file options, numoutlieriter: 0, numoutlierevec: 0, numoutevec: 10, newshrink: YES, hiprecision: YES, and lsqproject: YES.

#### Ancestry decomposition

We used two separate approaches to model the ancestry of the Bakr Awa individuals:

1. For our ancestry modeling of principal components with non-negative least squares (PANE) 1.2.0 [48], we fixed five sources that demarcate the ancestral variation of Near Eastern populations as observed in PCA: sLevant_HG, nwAnatolia_N, cZagros_N, sCaucasus_HG, and EEHG. The individual genetic IDs associated with each analysis group are recorded in Additional file 2: Data S6. Given the substantially larger sample size of southern Caucasus Bronze Age individuals (*N* = 116), we randomly sampled 20 individuals for plotting purposes.
2. We performed model-based clustering analysis with fastmixture [51] in unsupervised mode using ancient individuals at K = 2–12 and cross-validation = 5. We used PLINK v1.9.0-b.8 to remove variants with minor allele frequency of 0.05 and linkage disequilibrium (LD) pruning with parameters --indep-pairwise 200 25 0.5. We plotted the fastmixtrue results with the package AncestryPainter 2.0 [106].

#### D-statistic

We further evaluated the unique ancestry present within each Bakr Awa analysis group through symmetry D-statistic analysis of form D(Bakr Awa_i_, Bakr Awa_j_; Test, Mbuti.DG) using the qpdstat function from admixtools2 v.2.0.0 [46, 98] with parameters allsnps = TRUE and f4mode = FALSE. To test the degree of allele sharing between the Bakr Awa Bronze and Iron Age groups, we performed a D-statistic test of form D(Test, Bakr Awa_Bronze Age_; Bakr Awa_Iron Age_, Mbuti.DG) with the same parameter values. Because this D-statistic configuration violated published guidelines, we investigated whether the affinity between the two periods was inflated due to technical artifacts. To evaluate this, we restricted our standard 1240k dataset to the "Compatibility" panel [39], a filtered SNP panel consisting of approximately one million SNPs successfully assayed across many published ancient human DNA datasets. Using the convertf utility from EIGENSTRAT, we supplied a list of incompatible markers to the badsnpname parameter, yielding a conservatively filtered dataset of 700,585 valid autosomal SNPs. We quantified the impact of this filtering by calculating Lin’s Concordance Correlation Coefficient (CCC) and Mean Bias between the unfiltered and filtered datasets across D-statistic estimates, Z-scores, and Standard Errors. Finally, we isolated the 20 most divergent D-statistic estimates (|Z|> 3) to determine if the applied SNP filtering systematically altered both the direction and statistical significance of the genetic affinity signals (Additional file 1: Fig. S12).

#### *f*_3_-statistic

All *f*_3_-statistic tests were computed in admixtools2 v.2.0.0 [46, 98] using the qp3pop function with parameters allsnps = TRUE. We evaluated the genetic distance between Bakr Awa and ancient *Test* groups through the outgroup *f*_3_-statistic of form *f*_3_(Mbuti.DG; Bakr Awa_i_, *Test*). To assess which ancient groups are consistent with being a mixture to form the Bakr Awa group ancestry, we performed an admixture *f*_3_-statistic of form *f*_3_(Bakr Awa group; *Test*_*A*_, *Test*_*B*_). We were not able to analyze the Bakr Awa MBA outlier individual, as it is a single individual. To account for the multiple testing of admixture statistics, we applied the Benjamini-Hochberg (BH) procedure to control the False Discovery Rate (FDR). We subsequently calculated 'adjusted Z-scores' to provide a standardized measure of significance that incorporates the FDR penalty. These were derived by applying the inverse standard normal cumulative distribution function (quantile function) to the halved adjusted *p-*values, assuming a two-tailed test.

We performed pairwise outgroup *f*_3_-statistic tests of form *f*_3_(OldAfrica; *Test*_*A*_, *Test*_*B*_) between ancient individuals from six meta-geographic regions (described below) to determine significant differences in the distribution of inter-regional genetic similarity. These values were subsequently converted to a dissimilarity metric 1-*f*_3_. To evaluate differences in internal genetic homogeneity within each time period, we restricted our distributional analyses strictly to within-region pairs. We applied omnibus Kruskal–Wallis rank-sum tests to assess global differences in within-region genetic affinity across all meta-regions, followed by pairwise Wilcoxon rank-sum tests to compare the 1-*f*_3_ distributions between meta-regions. The resulting *p*-values were adjusted for multiple testing using the Benjamini–Hochberg procedure. We additionally evaluated genetic homogeneity by calculating the multivariate homogeneity of group dispersions using the betadisper function in the R package vegan 2.7–2 [107]. For this analysis, we constructed full individual-by-individual 1-*f*_3_ dissimilarity matrices for each time period. Because the 1-*f*_3_ values are non-Euclidean, we independently applied both Cailliez corrections and square-root transformations to the distance matrices prior to eigen-decomposition. Dispersion was quantified by calculating the distance of each individual to the spatial median of its respective meta-region in principal-coordinate space, providing robustness against outliers. We tested for global differences in dispersion using ANOVA and permutation tests (999 permutations) and performed pairwise comparisons of group homogeneity using Tukey's Honestly Significant Difference (HSD) tests (Additional file 1: Fig. S8; Additional file 2: Data S9).

#### qpWave regional spatiotemporal clustering

We used qpWave testing implemented in admixtools2 v.2.0.0 [46, 98] to determine whether each Bakr Awa individual formed a genetic clade with ancient individuals from six surrounding macro-regions: 1) Caucasus, 2) Zagros, 3) southeastern Anatolia and northern Mesopotamia, 4) northern Levant, southern and eastern Anatolia, 5) southern Levant, and 6) central, southwestern, and northern Anatolia. For each Bakr Awa individual, we conducted qpWave/qpAdm analysis at rank = 0 against a diverse panel of 15 right-groups: OldAfrica, WHGA, EEHG, YamnayaCluster, neIran_ChL, cZagros_N_GanjDareh, nwZagros_N, sCaucasus_HG, sCaucasus_N, nMesopotamia_PPN, seAnatolia_PPN_Cayonu, nwAnatolia_N, sLevant_HG, sLevant_PPN, and sLevant_ChL. All analyses were performed with parameters allsnps = TRUE and fudge_twice = TRUE. Comparisons with fewer than 30,000 overlapping SNPs were excluded. To account for multiple-hypothesis testing, we applied Benjamini–Hochberg correction [108] to the resulting qpWave *p*-values independently within each Bakr Awa individual's set of pairwise tests. Corrected *p*-values ≥ 0.05 were interpreted as evidence that a Bakr Awa individual formed a genetic clade (i.e., clade failed to be rejected) with a given ancient individual.

To determine if Bakr Awa individuals possessed an enrichment in significant qpWave clade connections with individuals from specific geographical regions through time, we first implemented a non-parametric, permutation-based enrichment test using custom scripts in R [109]. This test was applied independently across the three time-period bins (TP:1–3) defined roughly relative to the Bakr Awa Bronze Age: (3.4–1.2] ka BP, [4.2–3.4] ka BP, and (7.7–4.2) ka BP. For each Bakr Awa individual, we defined the observed test statistic (*k*_obs_) as the total number of clade-forming pairs (BH corrected *p*-value ≥ 0.05) associated with a given target meta-region. To assess statistical significance, we generated an empirical null distribution by randomly permuting the six geographical-region labels assigned to the comparative populations *B* = 10,000 times. Importantly, this permutation procedure preserves the total number of significant connections and the total number of individuals per region, but randomizes the association between the two.

For each permutation, *i*, we calculated the number of significant qpWave connections assigned to the test geographical region (*k*_perm, *i*_) and calculated an empirical one-sided *p*-value using the formula:

$$p = \frac{\sum_{i=1}^{B} I(k_{\mathrm{perm},i} \ge k_{\mathrm{obs}}) + 1}{B + 1}$$

where $\mathrm{I}(\cdot)$ the indicator function assigns a value of 1 to permutations where the randomized significant qpWave connections assigned to the test geographical region equaled or exceeded the counts for empirical data (*k*_perm_ ≥ *k*_obs_), and 0 otherwise. An empirical *p*-value then is calculated as the proportion of permutations where the randomized qpWave significance count assigned to the test region was greater than or equal to the observed test region's observed count. A pseudocount is added to both the numerator and denominator to ensure *p*-values are never exactly zero.

Because we observe the distributions of individual-based outgroup *f*_3_-statistic tests of form *f*_3_(OldAfrica; *Test*_*A*_, *Test*_*B*_) differ between meta-geographical regions, we also ran a Bayesian Generalized Linear Mixed Model (GLMM) framework using the MCMCglmm [110] package in R with the matrix of individual-based outgroup *f*_3_-statistic tests included as a random effect to capture the background genetic structure. Over each of the three time-period bins ((3.4–1.2] ka BP, [4.2–3.4] ka BP, and (7.7–4.2) ka BP), we used the binary outcome of cladality (BH corrected qpWave *p*-value ≥ 0.05) as our response variable, the macro-region as the fixed effect predictor, and employed a categorical family specification for binary logistic regression. The genetic relatedness matrix was generated by transforming *f*_3_-statistic estimates to a similarity metric (1-*f*_3_), which was then projected to the nearest positive semi-definite matrix using Higham's algorithm (Matrix v 1.7–4 [111–113]). Prior to inversion for the ginverse argument, eigenvalues were evaluated; if any fell below 10^–8^, a small ridge penalty (10^−6^I) was added to ensure stability. Individual-by-time-period subsets that contained fewer than 5 observations or lacked regional contrast were excluded. *f*_3_-statistic matrix was checked for positive semi-definiteness, inverted, and supplied to the ginverse argument in MCMCglmm.

For the fixed effects, we employed a multivariate normal prior with mean zero and variance–covariance matrix equal to five times the identity matrix. We fixed the residual variance at one, and for the random effect variance component, we used a parameter-expanded prior with V set to 1, nu equal to 1, alpha.mu equal to 0, and alpha.V equal to 1000. We executed four independent Markov Chain Monte Carlo chains in parallel, each running for 5,000,000 iterations with a burn-in period of 500,000 iterations and a thinning interval of 2,500. Chain convergence was assessed using the Gelman-Rubin diagnostic, with potential scale reduction factors calculated for all fixed effect parameters. Stationarity was evaluated using the Heidelberger-Welch diagnostic, and effective sample sizes were computed to verify sufficient independent samples for reliable inference.

Model validation incorporated posterior predictive checks (PPC) using the bayesplot [114] package to assess both calibration (mean prediction) and distributional fit. PPCs and classification performance – quantified using recreceiver operating characteristic (ROC) analysis and area under the curve (AUC) calculated via the pROC [115] package – were calculated using marginal (fixed-effects-only) predicted probabilities to conservatively test the discriminative ability of the geographic regions independent of individual-level random effects. Additionally, we checked for potential confounding between fixed and random effects by visualizing the distribution of random effect estimates across macro-regions.

Final inference was based on combined posterior samples from all four chains. We report posterior means (log-odds), 95% highest posterior density (HPD) intervals, odds ratios, and Bayesian *p*-values (pMCMC) calculated as twice the minimum of the proportion of samples above or below zero. We calculated the proportion of total variance attributable to the genetic random effect as Vg/(Vg + π^2^/3), where π^2^/3 represents the residual variance on the standard logistic distribution. Results meeting stringent criteria – pMCMC less than 0.05, proportion of genetic variance exceeding 0.9, and 95% HPD intervals excluding zero – were designated as high-confidence genetic affinity assignments.

#### Bakr Awa individuals qpWave hierarchical clustering

We formed analysis groups of the Bakr Awa individuals through qpWave clustering using admixtools2 v.2.0.0 [46, 98] with parameters allsnps = TRUE, whereby we performed pairwise tests of each Bakr Awa individual against a diverse set of 21 right-groups:

WHGA, EEHG, YamnayaCluster, neIran_ChL, cZagros_N_GanjDareh, cZagros_ChL_SehGabi, nwZagros_N, sCaucasus_HG, sCaucasus_N, sCaucasus_LNChL, sCaucasus_ChL_Areni1, nMesopotamia_PPN, seAnatolia_PPN_Cayonu, seAnatolia_ChL, eAnatolia_ChL, nwAnatolia_N, nLevant_ChL, sLevant_HG, sLevant_PPN, sLevant_ChL, and OldAfrica. We transformed the *p*-values to distance measures by taking their -log10 value and then performed hierarchical clustering on the -log10 transformed *p*-values with the pheatmap v.1.12 [116] software with the clustering_method = "average".

#### qpAdm ancestry modeling

All qpAdm ancestry modeling was performed with the admixtools2 v2.0.0 package [46, 98] with parameters allsnps = TRUE, full_results = TRUE, fudge_twice = TRUE. We performed individual-level ancestry testing using a set of source populations more ancestrally close to the Bakr Awa individuals: cZagros_ChL_SehGabi, eAnatolia_ChL, sCaucasus_LNChL, sLevant_PPN, and YamnayaCluster. In this qpAdm test, we set the following outgroups: OldAfrica, Russia_MA1_UP.SG, Russia_UstIshim_IUP_snpAD.DG, nwAnatolia_N, cZagros_N_GanjDareh, sLevant_HG, EEHG, and sCaucasus_HG. We standardized the qpAdm ancestry weight estimates by projecting the vector of estimated weights for each individual onto the probability simplex using an efficient projection algorithm [117]. This algorithm computes the Euclidean projection of a vector onto the simplex, and the transformation identifies the set of weights that are non-negative and sum to one while minimizing the Euclidean distance to the original unconstrained estimates.

To test for population continuity at Bakr Awa, we evaluated whether Bakr Awa Bronze Age individuals could serve as genetic sources for the Bakr Awa Iron Age group using fixed qpAdm models. We systematically tested one-, two-, and three-way combinations of Bakr Awa Bronze Age individuals as potential sources. All analyses employed the qpAdm function with a fixed panel of 23 right-groups: OldAfrica, WHGA, EEHG, YamnayaCluster, neIran_ChL, cZagros_N_GanjDareh, cZagros_ChL_SehGabi, nwZagros_N, nwZagros_BA, sCaucasus_HG, sCaucasus_ChL, sCaucasus_LNChL, sCaucasus_BA, nMesopotamia_PPN, seAnatolia_PPN_Cayonu, seAnatolia_BA, nwAnatolia_N, nAnatolia_ChL, eAnatolia_ChL, nLevant_BA_Alalakh, nLevant_BA_Ebla, sLevant_HG, and sLevant_PPN.

#### Runs of homozygosity

We estimated runs of homozygosity (ROH) values in our ancient Bakr Awa individuals using the software hapROH (v0.63) [54] with default parameters. ROH was determined using the default genetic map of the software and 5008 haplotypes from the 1000 Genome as a reference panel [118], and SNPs were called for each chromosome and individual.

## Computational resources

Bioinformatic processing was performed on Adelaide University Phoenix High Performance Computing (HPC) cluster. Admixture *f*_3_-statistic and individual-based qpWave regional analyses were performed on the Pennsylvania State University Computing ROAR cluster. All other population genetic analyses were performed locally with the R statistical package v.4.3.2 [119] along with dplyr v.1.1.4 [120], data.table 1.14.10 [121], tidyverse v.2.0.0 [122], ggplot2 v.3.5.1 [123], ggpubr v.0.6.0 [124], ggrepel v.0.9.5 [125], ggmap v.4.0.0 [126], maps v.3.4.2 (10.32614/CRAN.package.maps), mapdata v.2.3.1 (10.32614/CRAN.package.mapdata), igraph v.1.6.0 [127], and viridis v.0.6.5 [128]. The authors gratefully acknowledge the data storage service SDS@hd supported by the Ministry of Science, Research, and the Arts Baden-Württemberg (MWK) and the German Research Foundation (DFG) through grant INST 35/1503–1 FUGG.

## Supplementary Information

Additional file 1: Archaeological and anthropological background Note S1, Stable isotope analysis Note S2, and Supplementary Figures S1 to S12.

Additional file 2: Supplementary tables Data S1-17.

## Data Availability

The new ancient genomes reported in this paper can be accessed at the European Nucleotide Archive (ENA) under the following study accession number: PRJEB111543 (ERS29682454: ERS29682480) (secondary accession ERP192152) [129]. The corresponding genotype dataset and analysis scripts are available at Zenodo (10.5281/zenodo.19963881) [109].
