# Supplementary material for: Mesopotamian ancient DNA reveals Iron Age integration of heterogeneous Bronze Age genetic ancestries following resettlement
Source: Genome Biol. 2026 Jun 12;27:260. doi: 10.1186/s13059-026-04145-4 (PMC13483502; doi:10.1186/s13059-026-04145-4)
Supplement: Supplementary file 1 [file 13059_2026_4145_MOESM1_ESM.pdf]

Supplementary Materials for

Mesopotamian ancient DNA reveals Iron Age integration of heterogeneous Bronze Age genetic ancestries following resettlement

Matthew P. Williams[1,2,3†*], Rafał Fetner[4], Yassine Souilmi[1,5†], Ulrike Bürger[6], Arkadiusz Sołtysiak[4†], Aidan Richards[3], Wolfgang Haak[7], Raymond Tobler[1,8†], Bastien Llamas[1,5,9,10†], Peter A. Miglus[6†], Alan J. Cooper[11†]

1. Australian Centre for Ancient DNA, Faculty of Sciences, Engineering and Technology, The Adelaide University, Adelaide, SA, 5005, Australia
2. Biology Department, The Pennsylvania State University, University Park, Pennsylvania, USA
3. Department of Biological Sciences, Walla Walla University, College Place, Washington, USA
4. Department of Bioarchaeology, Faculty of Archaeology, University of Warsaw, Warsaw, Poland
5. The Environment Institute, School of Biological Sciences, Adelaide University, Adelaide, SA, 5005, Australia
6. Institute for Pre-and Protohistory and Near Eastern Archaeology, University of Heidelberg, Sandgasse 7, 69117 Heidelberg, Germany
7. Department of Archaeogenetics, Max Planck Institute for Evolutionary Anthropology, Leipzig, Germany
8. Evolution of Cultural Diversity Initiative, Australian National University, Canberra, ACT, 2601, Australia
9. National Centre for Indigenous Genomics, John Curtin School of Medical Research, Australian National University, Canberra, ACT, 2601, Australia
10. Indigenous Genomics, The Kids Research Institute Australia, Adelaide, SA, 5000, Australia
11. Gulbali Institute, Charles Sturt University, Albury, NSW, 2640, Australia

*Corresponding author: Matthew P. Williams (matthew.williams@wallawalla.edu)
[†] Senior authors

Note S1: Archaeological and anthropological background

Contact: Ulrike Bürger (ulrike.buerger@uni-heidelberg.de), Rafał Fetner (rafetner@uw.edu.pl), and Peter A. Miglus (peter.miglus@uni-heidelberg.de)

The Site

The site of Bakr Awa is situated in the Zagros piedmont of Iraqi Kurdistan, where the Diyala River has its source. With 40 ha, it is the largest *tell* in the southern part of the fertile Shahrizor Plain, comprising a central citadel mound and a surrounding lower town area.

Bakr Awa has been settled since the Late Chalcolithic times onwards. Layers of the 4th millennium BC were detected in the central part of the site in a sounding, where a pottery assemblage of beveled-rim bowls (BRB) and other jars of the Uruk period came to light. The eastern part of the lower town had not been settled until the Early Bronze Age, where in several deep soundings, virgin soil was reached directly below layers of the early 3rd millennium BC. The pottery from these layers included fragments of Scarlet Ware, a characteristic of the Early Dynastic period in the Diyala Region and neighboring Luristan (*1*).

Layers of the late 3rd millennium are characterized by architecture with stone foundations. Pottery has analogies to shapes and decorations of the Akkadian period, but fragments of the local Bakr Awa Painted Ware (BAPW) were found in contexts of the 22nd to 19th century BC as well.

During the late Early and Middle Bronze Age, the site became a prosperous city, well embedded within trans-regional exchange networks of the Ur III, Isin-Larsa, and early Old Babylonian periods. In the first half of the 2nd millennium BC, construction techniques and architecture equal those of the southern Mesopotamian heartland, while many artefacts are comparable to middle and north Mesopotamian as well as western Iranian types (*2*). Most of the samples examined in the present study were taken from the burials of this period; however, not all excavated burials are part of the present study.

Despite a short occupation hiatus at the end of the Middle Bronze Age, the city kept its function as an important administrative center during the Late Bronze Age, as proven by varied textual evidence with analogies in the Hurrian sphere. However, this occupation phase ended in a destructive fire around 1400 BC, and the site was abandoned for several centuries (*2*).

Settlement activities were resumed in the Iron Age, but to a lesser extent, and with a local material culture showing rather Urartian and Median influences than analogies with Mesopotamia or Assyria in particular. Several burials were exposed as well, some also dating to the subsequent Achaemenid period (*1*).

Little can be said about the situation at the end of the 1st millennium BC and during the first centuries AD, as neither any significant architecture or installations nor burials were discovered. Any traces of occupation would have been destroyed by the vast settlement and burial activities of the Islamic Middle Ages, which also affected the deeper occupation layers (*1*).

In Modern Times, the site was still occupied by a rural village until the 1980s, when the houses were destroyed, and its inhabitants forcibly resettled during the Iraq-Iran war and Saddam Hussein's Anfal campaign. During the embargo in the 1990s, the site was deeply affected by looting activities, leaving the archaeological site literally scarred. It was only in the 2010s, in the context of a national reparation initiative, that the Bakr Awa village was resettled.

Archaeological research

Early digging activities at Bakr Awa were allegedly initiated by Captain George Martin Lees, who held a post in the British Mandate administration in the early 1920s at nearby Halabja (*3*, *4*). Just a few years later, Ephraim Avigdor Speiser examined the existing trenches, which he attributed to treasure hunters, and had some soundings conducted himself (*5*).

Proper archaeological fieldwork was not instigated until the beginning of the construction of the Darband-i Khan Dam, 1956–61, when the Directorate General of the Iraqi Antiquities Service surveyed the affected area, as the newly created reservoir would flood large parts of the southern Shahrizor Plain. During the project led by Muhammad Ali Mustafa, around 20 settlement mounds were documented in the so-called Darband-i Khan Basin. In 1959 and the following years, additional rescue excavations were carried out at some of the sites, including Bakr Awa 1960–61(*3*, *4*).

The political situation in Iraq in the second half of the 20th century prevented further investigations in the region until the end of the Second Gulf War. Archaeological research was resumed in cooperation with the Slemani Antiquities and Heritage Directorate, when Peter A. Miglus and his team from the University of Heidelberg first conducted a survey in the Shahrizor Plain in 2009, and subsequently started excavating at Bakr Awa in 2010. Six seasons of fieldwork were carried out in 2010+11, 2013+14, and 2017+18, mainly focusing on the Bronze Age occupation (*6*, *7*).

The Burials

Late Early Bronze Age [ca. 2300–2000 BC]

The late phase of the Early Bronze Age is contemporary with the historical Akkadian and subsequent Ur III period. During this time, the settlement at Bakr Awa is characterized by buildings with strong stone foundations, one of them a small shrine, but just a few infant burials were found in these layers.

<u>Burial BA1399</u>

Earth grave

- A22041 (BA1399/003): Male (genetically)

The grave contained the remains of a newborn found without any burial goods. The state of preservation was good, with the skull, thorax, and both limbs present. A pathological condition, cribra orbitalia, was noted. This one is commonly attributed to a stress associated with malnutrition.

<u>Burial BA2304</u>

Pot burial

- A22047 (BA2304/003): Male (genetically)

The grave contained the remains of a newborn found in the pottery jar. The state of preservation was good, with skull, thorax, and both upper and lower limbs present.

Transitional late Early Bronze Age – early Middle Bronze Age (EMBA) [around 2000 BC]
Several burials derived from disturbed stratigraphic contexts barely provided any datable grave goods; furthermore, their radiocarbon dates span over two centuries each. Some of them seem to have still been contemporary with the last occupation phase of the late EBA buildings. This is especially the case for tomb BA1400 (not part of the present study), which is the earliest chamber burial and the only one built of stone at Bakr Awa so far. Some graves must be slightly younger, but still predate the main MBA mud brick architecture. Therefore, a more precise assignment than transitional late Early to early Middle Bronze Age is not suitable. It is noteworthy that some of the individuals from the graves show signs of violence, like the pregnant woman with a head injury in BA1311, or were rather disposed of than properly buried, like the human remains in oven BA2265.

<u>Burial BA1311</u>
Earth grave

- A22033 (BA1311/001):

The grave contained the remains of a young adult female who was pregnant at the moment of death. The state of preservation was good, with skull, thorax, and both upper and lower limbs present. She bears a healed head injury (blunt force trauma) and fractures of the right metacarpals.

- A22034 (BA1311/005):

Remains of a foetus were found in the anatomic order at the level of the abdomen. The state of preservation was good, with skull, thorax, and both upper and lower limbs present.

<u>Burial BA1314</u>
Earth grave

- A22037 (BA1314/001):

The grave contained the remains of an adolescent (14–15 years old). The preservation was good, with the skull, thorax, and both upper and lower limbs present. Linear enamel hypoplasia indicates stress episodes during childhood. Similarly, the osteoarthritis of the second cervical vertebra.

<u>Burial BA1348/1387</u>
Secondary burial, disposal?

- A22038 (BA1348/001A):
- A22039 (BA1348/001B):

The pit contained a commingled assemblage of human remains. The assemblage contained elements of cranial and post-cranial skeleton, with bones of the thorax, upper and lower limbs present. Selected skulls represent two adult individuals whose remains were found in the assemblage. Due to the commingled nature of the assemblage, the pathological changes of the post-cranial skeleton cannot be ascribed to one or other individual. Evidence of caries was found on the premolars of both individuals. Post-cranial lesions include osteoarthritic changes of the right acetabulum.

Burial BA2265

Oven burial / secondary burial, disposal?

- A22045 (BA2265/002):
- A22046 (BA2265/006):
- A20578 (BA2265/006):

The oven contained remains of an adult female (BA2265/006), and human remains in a secondary context (BA2265/002). The remains of the female were in good condition, including the skull, thorax, and both upper and lower limbs. Among pathological changes, fracture of the vertebra and caries were present.

Middle Bronze Age [ca. 2000–1600 BC]

During the Middle Bronze Age, the urban development at Bakr Awa reached a climax. At the turn from the 3rd to the 2nd millennium BC, the construction technique at Bakr Awa shifted from stone to mudbrick architecture. The layout and features of a residential courtyard house and the new type of pitched-brick vaulted tombs exemplify strong connections to the southern Mesopotamian heartland. Richly equipped tombs and burials with jewelry and weapons prove that it was, overall, a period of wealth and prosperity. Materials like precious stones were imported from far away, proving that the city was integrated into a long-distance trade network. At least three occupation phases can be distinguished, each characterized by specific pottery types.

MBA phase 1 (ca. 2050–1950 BC)

The earlier phase is contemporary with the late Ur III and early Isin-Larsa periods. This is the founding period of the aforementioned courtyard house. Ovoid beakers with tall and wide cylindrical necks were found in tombs BA2950 and BA2500 (both not part of the present study), and have analogies in the South (*8*).

Burial BA2604

Pot burial

- A22051 (BA2604/001):

The grave contained the remains of a newborn. The state of preservation was good, with the skull, thorax, and both upper and lower limbs present.

MBA phase 2 (ca. 1950–1800 BC)

The main occupation phase is contemporary with the Isin-Larsa period. Characteristic Istakan beakers with a round base and carinated cylindrical body were found in these settlement layers and drainage shaft BA2671, as well as in tombs BA1108 and BA5122* (not part of the present study). Rare analogies are only known from Luristan (Haerinck – Overlaet 2008, 19 Fig. 8, 8–11, Pl. 3), the Shahrizor and Raniya Plains (Eidem 2020, 180 Pl. 1, 1–5: Shemshara, pre-Kuwari layers VIII+VII).

Tomb BA1108

Pitched-brick tomb

- A22022 (BA1108/152):
- A22023 (BA1108/172):
- A22032 (BA1108/079):

- A22035 (BA1108/156):
- A22036 (BA1108/083):

Tomb BA1108 contained the remains of eight individuals, six adults and two subadults. The minimum number of individuals (MNI) for adults was calculated based on the presence of unique bones, in this case, 6 axis vertebrae, 6 frontal bones, 6 right hamate, 6 left 4th metatarsals, and 6 pairs of temporal bones. The MNI of subadults was estimated based on the number of unique bones (calcaneus, distal epiphysis of right femur, mandible, left pubic bone, and proximal diaphysis of right tibiae), and stage of bone maturation and length of long bones. In most cases, age-at-death was estimated based on a single method, and therefore, it can serve as general information about age only. Molar teeth were moderately worn, indicating rather mature adults. This observation is supported by a single pelvis of a male individual, also expressing morphological changes typical of a mature adult. Comparison of the age-at-death indicators suggests that individuals buried here were more commonly mature adults. Remains of subadults were separated from the adult bone assemblage. The age-at-death of the subadults was assessed based on dental development as a 6-year-old child and second as a 10-year-old child.

MBA phase 3 (ca. 1800–1700 BC)

The latter phase is contemporary with the late Isin-Larsa and early Old Babylonian periods. Fine ware beakers ("Hurrian cup") with a small pedestal foot, round body, and high funnel neck were found in several burials and tomb BA5122*, as well as in the settlement layers. Noteworthy is especially room BA2300 with its filling BA2295 (1947–1746 BCcal), which in our previous publications has been erroneously dated to the Late Bronze Age. Close analogies for the beakers are known from the Diyala region (*9*), Raniya Plain (*10*), and from Yorgan Tepe (*11*). Signs of disturbance mark the end of this occupation phase at Bakr Awa. For example, the broken pottery ensemble in Room BA2300 remained in situ, and incomplete tomb BA5122* was just built pars pro toto as a cenotaph. The site was briefly abandoned, probably as a consequence of an earthquake.

<u>Burial BA2084</u>

Earth grave / secondary burial

- A22043 (BA2084/011):

Grave BA2084 contained the remains of two individuals, a mature adult male (BA2084/11) in the anatomic order and a young adult female (BA2084/1) in the secondary position. Preservation of both skeletons was good, with bones of the skull, thorax, and both upper and lower limbs present. The male individual expressed severe hypoplastic changes of enamel, as well as caries, and ante mortem tooth loss. The female skeleton expresses only carious changes.

<u>Burial BA2358</u>

Earth grave

- A22048 (BA2358/001):

The grave contained the remains of a mature adult male individual. The preservation was good; however, only the bones of the skull, thorax, and pectoral girdle were available for the analysis. Degenerative bone disease was noted on the sterno-clavicular joint of both sides. Caries was noted on premolar teeth, and a periapical cyst was noted in the maxilla at the root of the left

upper premolar. The newly formed bone formation noted on the external surface of the ribs could be the effect of an inflammation.

Burial BA2646
Earth grave

- A22052 (BA2646/001):

The grave contained the remains of a mature adult individual. The preservation was good, but only the skull, pectoral girdle, upper thorax, and a few carpals were present. Among pathological changes, ante mortem tooth loss, caries, hypoplasia, cribra orbitalia, and Schmoerl were noted.

Burial BA2688
Earth grave

- A22053 (BA2688/003):

The grave contained the remains of a mature adult female. The preservation was good with the skull, thorax, upper and lower limbs present. Among pathological changes, osteoarthritis of the four cervical vertebra was noted.

Burial BA2696
Earth grave

- A22054 (BA2696/004):

The grave contains the remains of a child (ca. 8 years old). The state of preservation was good, with the skull, thorax, and both lower and upper limbs present. Among pathological changes, cribra orbitalia and linear enamel hypoplasia were noted, both indicating nonspecific stress.

Contextually ambiguous:
Next to the MBA tomb BA1108, an earth burial BA1375 was discovered. The base level of the interment equaled the vault's top level. Due to its location, it was impossible to determine from where the burial had been dug. The layers above were heavily disturbed by later occupation activities and slope erosion. Other graves in the vicinity on a comparable level date either to the Middle Bronze or the Iron Age. With no conclusive stratigraphic evidence, without any grave goods, which would allow for typological dating, and since no radiocarbon dating has been conducted yet, BA1375 preliminarily remains without a certain chronological assignment.

Burial BA1375
Earth grave

- A22040 (BA1375/001):

The grave contained the remains of a young adult male. The state of preservation was good, with the skull, thorax, upper and lower limbs present. No pathological changes were noted.

Iron Age [c. 1200–330 BC]
Layers of the 1st millennium BC were heavily affected by settlement and burial activities of the Islamic Middle Ages, as well as by modern looting pits. Therefore, our knowledge about the Iron Age occupation is fragmentary, and the attribution of a fortification wall surrounding the citadel mound is plausible but uncertain. In the eastern lower town, remains of stone pavements and the fire chamber of a kiln were preserved. Furthermore, several graves came to light, one (BA7022, not part of the present study) was equipped with iron tools. The associated pottery comprised a

reddish slipped ware with analogies in the Urartian and Median sphere of the 8th–6th century BC (*12*, *13*) instead of Neo-Assyrian ceramic types. This is quite a remarkable find because the Shahrizor Plain is supposed to have been part of the Neo-Assyrian province of Zamua at that time. A few other graves were of a younger date, related objects tie them to an overall Achaemenid sphere.

IA phase 1 (ca. 1000–550 BC)
Burial BA1049
Earth grave

- A22026 (BA1049/001):

The grave contained the remains of a young adult female. The preservation was good, with the skull, thorax, upper and lower limbs present. The pathological changes include caries and linear enamel hypoplasia.

Burial BA2940
Earth grave

- 22059 (BA2940/001):

The grave contained the remains of a child (about 4 years old). The preservation was good, with skull, thorax, and both upper and lower limbs present. Caries was noted on the second deciduous molar.

IA phase 2 (ca. 550–330 BC)
Burial BA1156
Earth grave

- A20576 (BA1156/001):

The grave contained the remains of a mature adult male. The preservation was good, with the skull, thorax, and both upper and lower limbs present. No pathological changes were noted.

Burial BA1157
Secondary burial

- A20577 (BA1157/001):

Bones were found in a secondary context, but they belong to a single individual, a young adult female. The preservation was good, with the skull, thorax, upper and lower limbs present. The pathological changes include antemortem tooth loss and inflammation of the alveolar process.

Burial BA1148
Earth grave

- A22025 (BA1148/003):

The grave contained the remains of an infant (about 2 years old). The preservation was good; however, only the skull and cervical vertebrae were available for the analysis.

Burial BA1085
Earth grave

- A22027 (BA1085/001):

The grave contained the remains of a child (about 11 years old). The preservation was good, but only the skull, cervical vertebrae, and upper thorax were available for analysis. Linear enamel hypoplasia was noted on canines and premolars.

Note S2: Stable isotope analysis

Contact: Rafał Fetner (rafetner@uw.edu.pl) and Arkadiusz Sołtysiak (a.soltysiak@uw.edu.pl)

Bioavailable strontium and oxygen background

of Shahrizor Plain and the neighboring hill. The data were completed by analogous measurements published by (*14*) for Bestansur (NW Shahrizor) and supported by the studies of groundwater (*15*). The local values of the oxygen stable isotopes were assessed based on (https://wateriso.utah.edu/waterisotopes/pages/data_access/oipc.html) (*16*, *17*). Plant strontium isotope values vary between 0.70798 and 0.70815, with the lowest value observed in the hill area near Darband-i Gawra passage; others sampled around the Bakr Awa express values between 0.70799 and 0.70815. Analogous samples from Bestansur express the variation between 0.70815 and 0.70816 for the Plain, and 0.70795 and 0.70803 for the hills (*15*).

Those measurements can be supported by the geochemical studies of groundwater in the Tanjero (main river of Shahrizor) water shade, which shows variation of Sr isotope ratio values between 0.7080 and 0.7082 (*15*). Therefore, the values between 0.7080 and 0.7082 can be considered as local for the Shahrizor Plain, bearing in mind that local hills can express slightly lower Sr values. The calculated annual mean δ18OSMOW value of the modern precipitation in the region of Halabja is -5.6 per mill (95% CI: -5.1 and -6.1 per mill). Therefore, with some tolerance, due to various effects impacting the δ18O values of drinking water and the tissues of the consumer (especially during the first years of life). This ratio can be considered local for the region of Shahrizor.

Individual measurements

As an early developing tooth, the first permanent molar represents the first years of an individual's life (e.g., (*18*)). The strontium isotope ratio was measured for 12 individuals. The 87Sr/86Sr value varies between 0.70797 and 0.70838, with 8 values within the estimated local range of Shahrizor Plain (see Additional file 2: Data S2: strontium values). The oxygen stable isotope ratio was measured for 11 individuals (except for BA2265/006 due to the low amount of available enamel). The oxygen stable isotope ratio varied between -7.8 and -2.9 per mill, with 3 clear outlying values (Additional file 2: Data S2: oxygen values).

The place of origin is difficult to assess, as the mapping of bioavailable strontium in Southwest Asia is far from sufficient for this type of study. Hence, only areas where specific strontium and oxygen isotope ratios co-occur can be indicated. Those areas can be, with much caution, treated as potential places of the individual's origin. Local origin can be ascribed to a total of 6 individuals: A22026, A22023, A22035, A22036, A20577, and A22052. While others might come to Shahrizor from different environments. The isotope values measured for A20576 co-occur in the foothills of Zagros, not far from Bakr Awa, and possibly indicate a short-distance migration. Values of two individuals (A22059 and A22033) co-occur in Southern Mesopotamia, while values of A22040 co-occur in the Mesopotamian Plains (e.g., the west of Bakr Awa).

Finally, values of A22037 co-occur in the Iranian Zagros (e.g., the East of Bakr Awa). For a single individual, A22046, only the Sr ratio was measured, and those values are present in many regions of SW Asia. Values of oxygen stable isotopes are more difficult to interpret. The meta study by (*19*), based on 4,085 analyzed individuals from 316 sites, indicates that intra-sample variability of δ18O in dental enamel of human individuals coming from an archeological context is usually more than 3 per mill. Moreover, in the samples below 25 individuals, the statistical approach should be avoided to draw any conclusions. Therefore, this figure will be used to identify potential non-local individuals. However, this approach can produce a false positive and should be used with caution (*19*). Following this recommendation, the local range for human individuals should be between -7.1 and -4.1 per mill, hence the following individuals bear the potential of non-local origin: A22037, A22033, A22059. Considering both isotopic systems, only one individual can be safely pointed out as a migrant: A22037. The case of A22046 remains unclear because interpretation can be based only on the strontium isotope value, which indicates a non-local origin. Analysis was carried out using the R language for statistical computing (*20*) in RStudio (2023.12.0), with assistance of the ggplot2 package (*21*). Maps were generated using QGIS software (v. 3.34).

Supplementary Figures

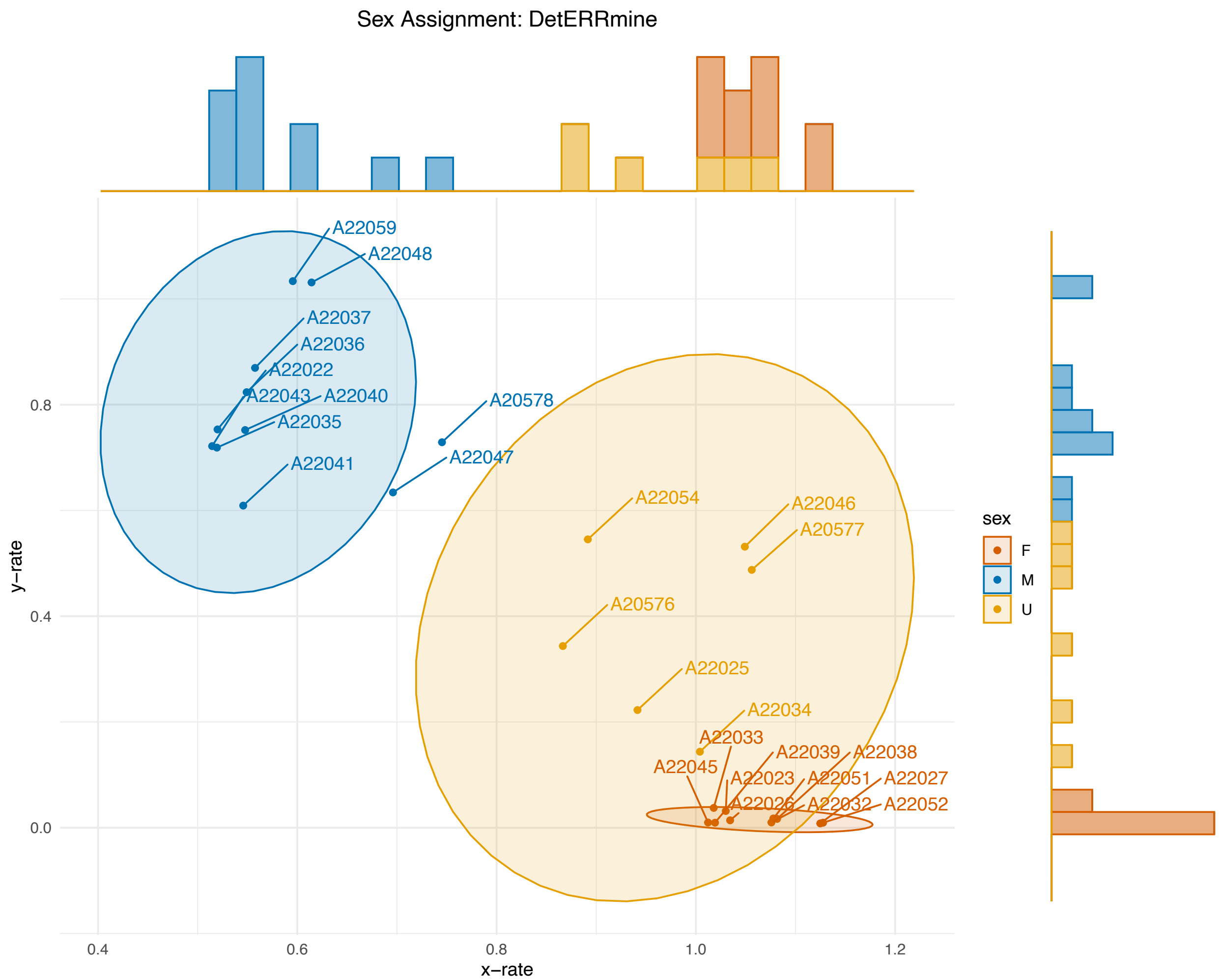

Fig. S1: Genetic sex assignment of the Bakr Awa individuals.
Genetic sex was estimated using the DetERRmine algorithm, which quantifies relative DNA read coverage across autosomal and sex chromosomes. The scatter plot displays the relative X-chromosomal coverage (x-rate) against Y-chromosomal coverage (y-rate) for each individual. Females characteristically present with an x-rate of ~1 and y-rate of ~0, while males exhibit x- and y-rates of approximately 0.5. Among the Bakr Awa samples, six individuals displayed intermediate x- and y-values (clustered in the center) and were classified as "genetic sex undefined." As documented in the manuscript methods and Supplementary Data 1, all specimens within this undefined category demonstrated evidence of DNA contamination and were excluded from downstream population genetic analyses

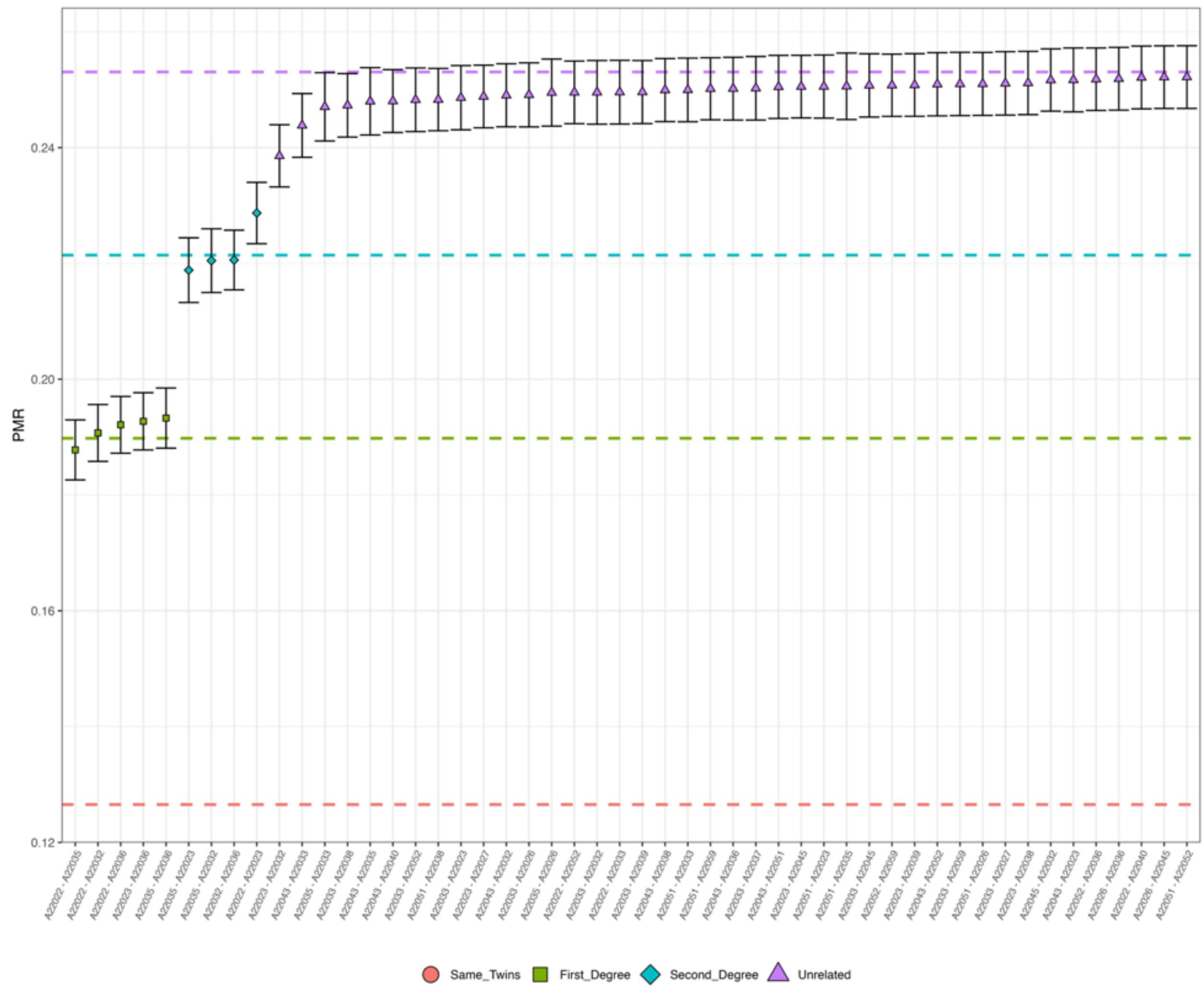

Fig. S2: Kinship estimation of the Bakr Awa individuals.
Kinship among the Bakr Awa individuals was estimated using the BREADR algorithm (v.1.0.1), which quantifies identical, first-, and second-degree kinship relationships through computational analysis of pairwise mismatch rates derived from genome-wide pseudo-haploid genetic data. The plot visualizes the pairwise mismatch rates, allowing for the identification of related individuals. The analysis identified five first-degree and four second-degree relationships within the dataset. To avoid analyzing close relatives in population genetic statistics, only the highest-coverage individual from each identified kinship group was retained for downstream analysis.

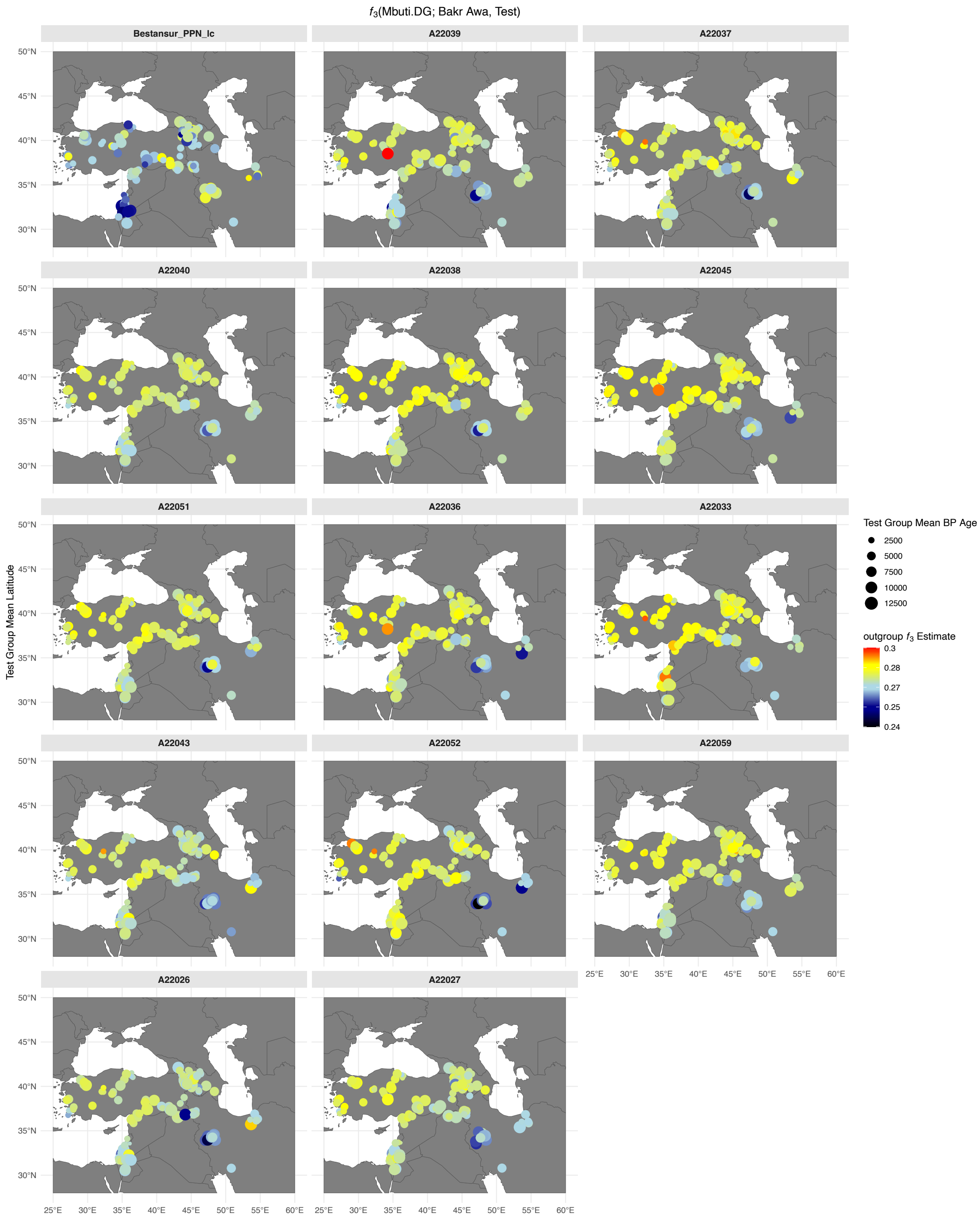

Fig. S3: Spatiotemporal Genetic affinity. Spatial distribution of shared genetic drift calculated using outgroup f_3-statistics of the form f_3(Mbuti.DG; Transtigris Group; Test Population). Warmer colors indicate higher shared drift. (Top Left): The Pre-Pottery Neolithic (PPN) Bestansur group (Bestansur_PPN_lc) exhibits strong genetic affinity to central Zagros-related populations, consistent with its geographic location. (Remaining Panels): In contrast, Bakr Awa Bronze Age individuals show a distinct ancestry shift, displaying predominant genetic links to Anatolian-related groups and secondary connections to northern Mesopotamian, Levantine, and

Caucasus-related populations. This illustrates the divergence between the PPN ancestry profile and the later Bronze Age population structure.

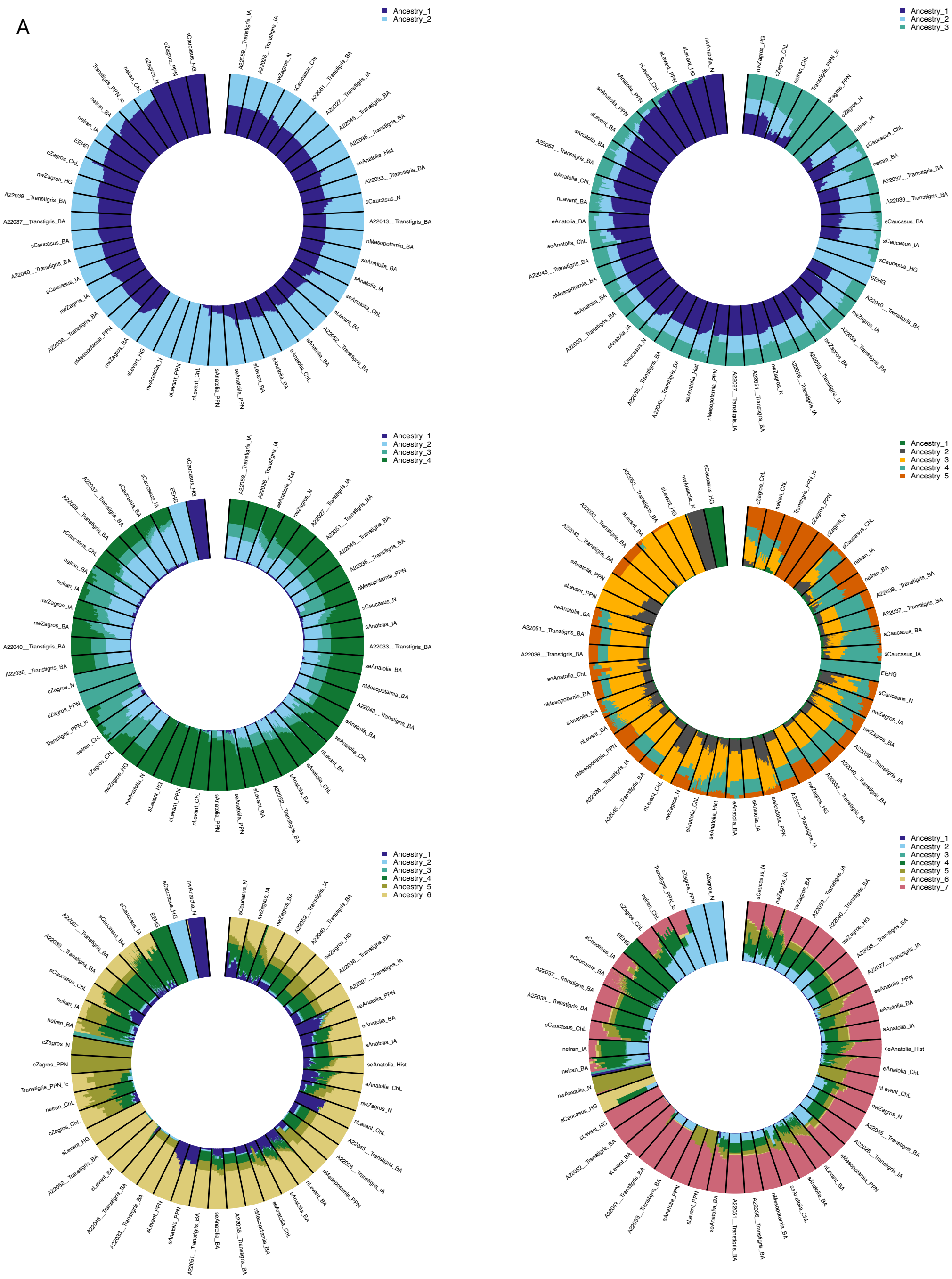
A
Ancestry_1
Ancestry_2
Ancestry_3
Ancestry_4
Ancestry_5
Ancestry_6
Ancestry_7

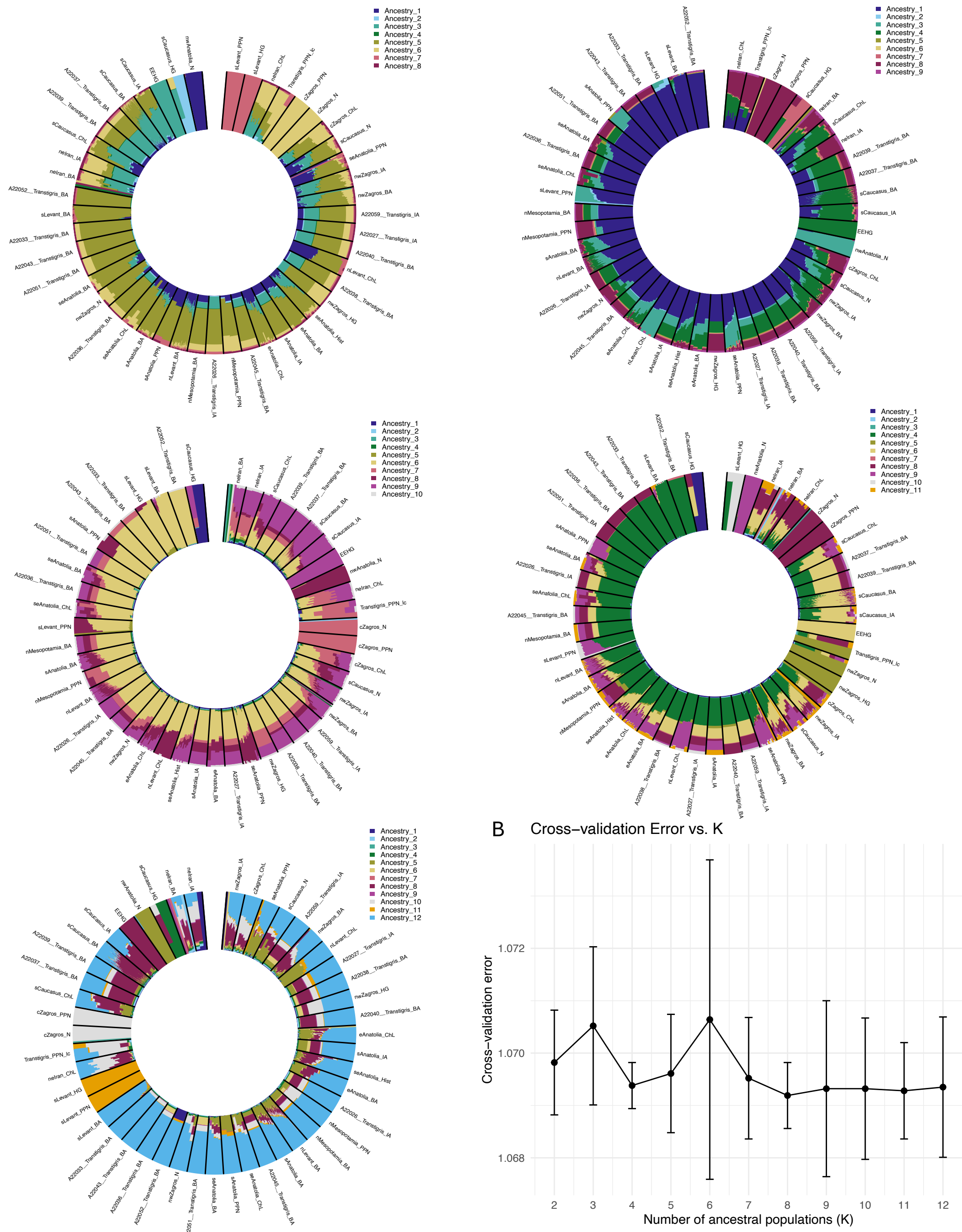

Fig. S4. Bakr Awa and Regional Ancestry Diversity

(A) Unsupervised model-based clustering performed using fastmixture for K=2–12. Each vertical bar represents an individual, colored by the proportion of ancestral components. (B) Cross-validation error plot for K=2 through K=12. Genotype data were processed using PLINK v1.9.0-b.8 to filter for minor allele frequency (0.05) and linkage disequilibrium (--indep-pairwise 200 25 0.5). The resulting ancestry proportions were visualized using AncestryPainter 2.0.

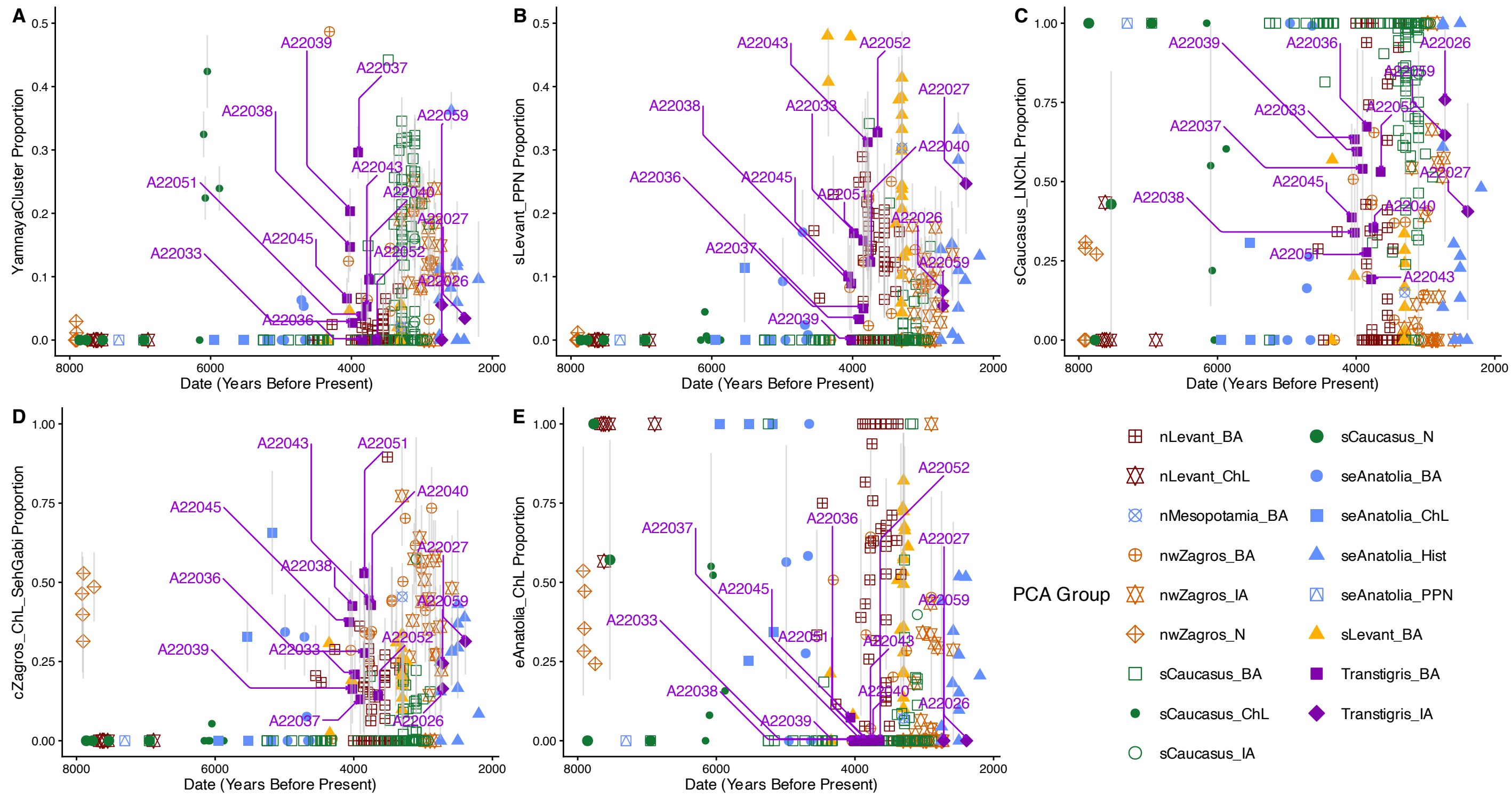

Fig. S5: Ancestral Near Eastern five-source qpAdm model.
Five-source qpAdm modeling of ancestries characterizing post-Neolithic Near Eastern population diversity: Central Zagros Chalcolithic (cZagros_ChL), Southern Caucasus Late Neolithic-Chalcolithic (sCaucasus_LNChL), Eastern Anatolia Chalcolithic (eAnatolia_ChL), Southern Levantine Pre-Pottery Neolithic (sLevant_PPN), and a Yamnaya-related Steppe metapopulation (YamnayaCluster) associated with Bronze Age southern Caucasus ancestry.

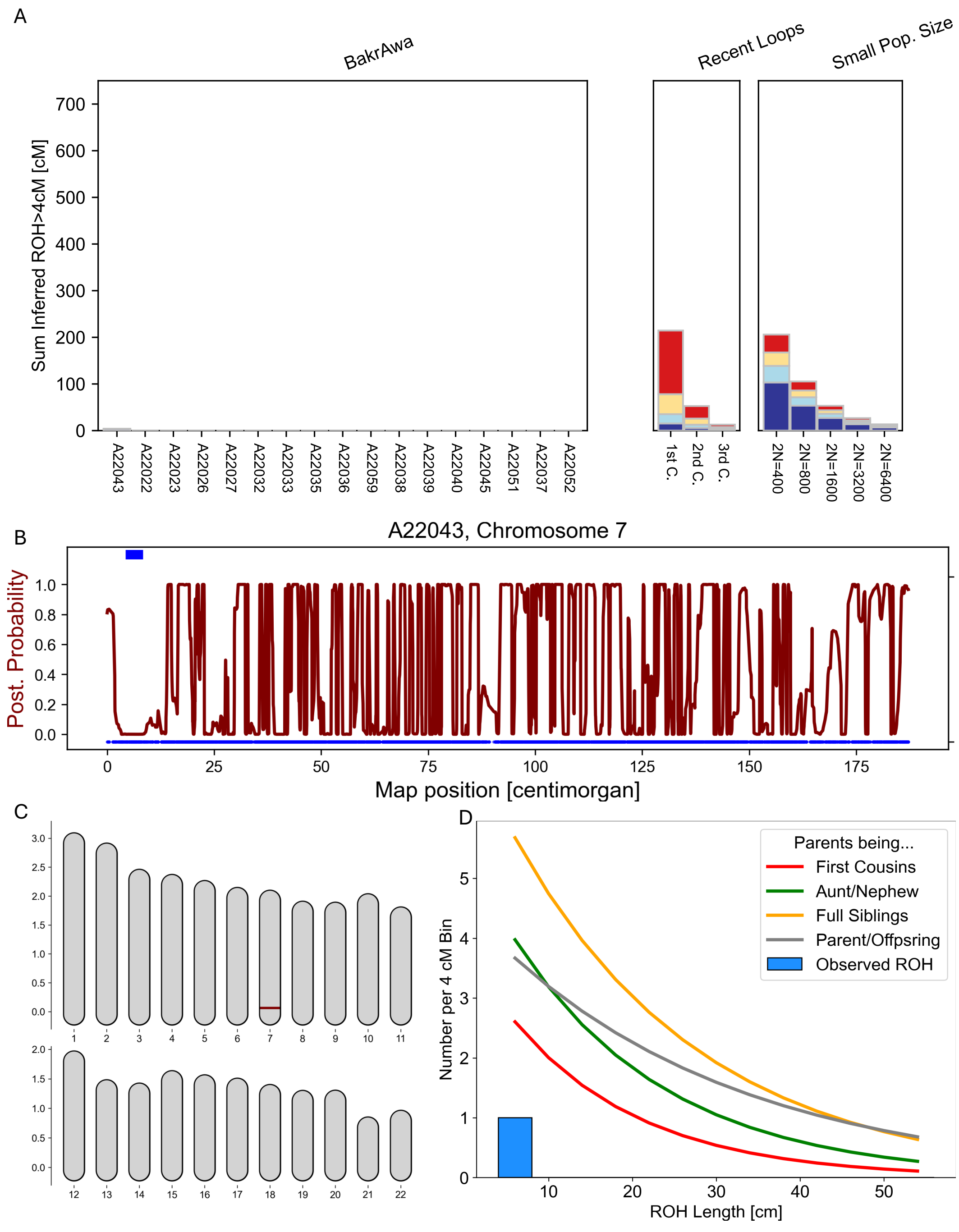

Fig. S6: Runs of homozygosity values.
(A) Each vertical bar represents an individual, with colored segments indicating the sum of runs of homozygosity (ROH) across four length classes (4–8, 8–12, 12–20, >20 cM). Bakr Awa individuals are shown on the left; expected ROH for offspring of close kin (first, second, or third cousins) or different effective population sizes (*Ne*), based on analytical calculations, are shown on the right. (B) Posterior probability of non-IBD state on chromosome 7 for A22043, which contains the longest and only inferred IBD segment. (C) Distribution of ROH across 22

autosomes (maroon), with map length annotated in Morgans. (D) Histogram of ROH lengths with expected densities for different degrees of parental relatedness.

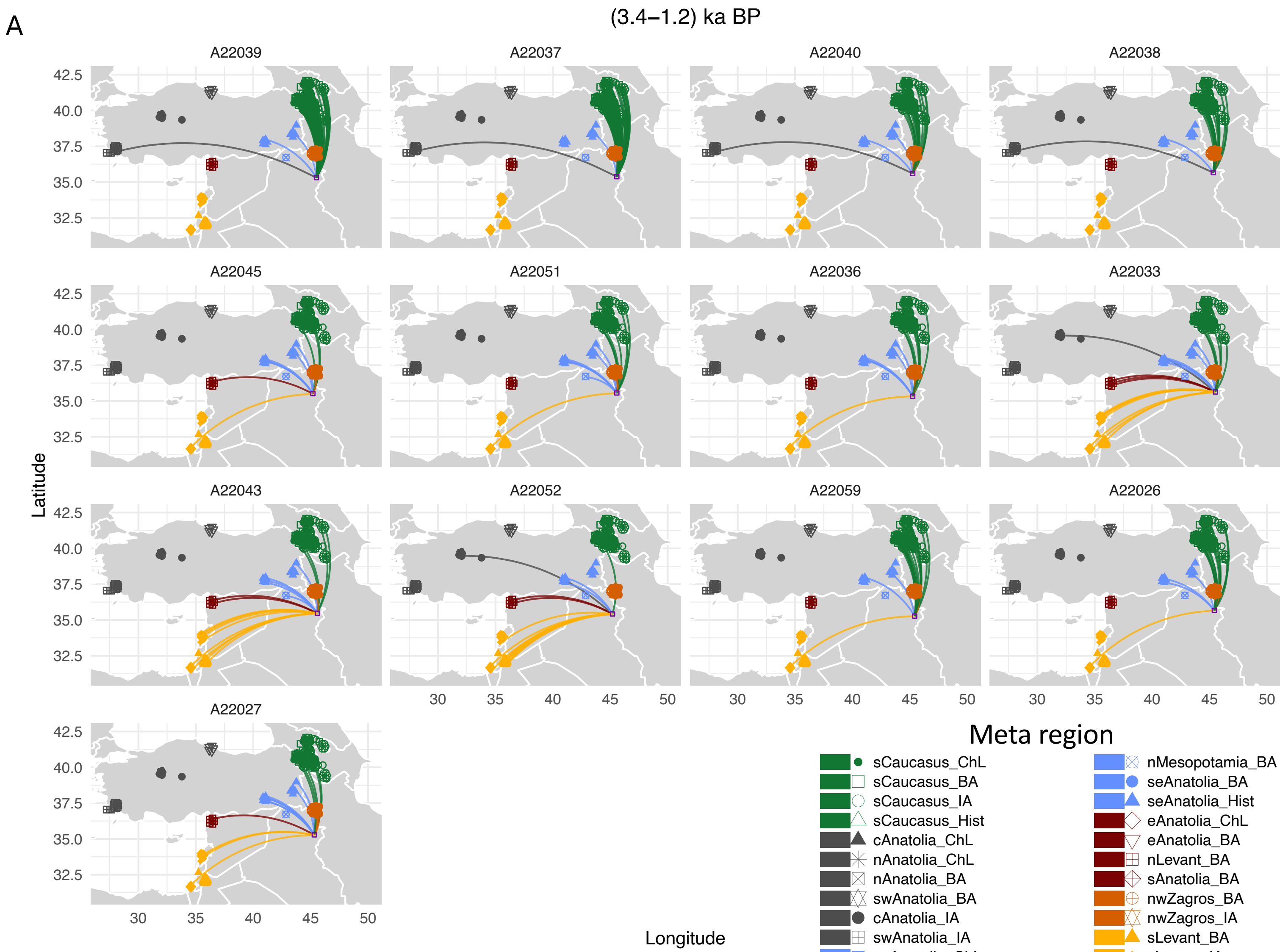
A
(3.4–1.2) ka BP
A22039
A22037
A22040
A22038
A22045
A22051
A22036
A22033
A22043
A22052
A22059
A22026
A22027
Latitude
Longitude
Meta region
sCaucasus_ChL
sCaucasus_BA
sCaucasus_IA
sCaucasus_Hist
cAnatolia_ChL
nAnatolia_ChL
nAnatolia_BA
swAnatolia_BA
cAnatolia_IA
swAnatolia_IA
seAnatolia_ChL
nMesopotamia_BA
seAnatolia_BA
seAnatolia_Hist
eAnatolia_ChL
eAnatolia_BA
nLevant_BA
sAnatolia_BA
nwZagros_BA
nwZagros_IA
sLevant_BA
sLevant_IA

B

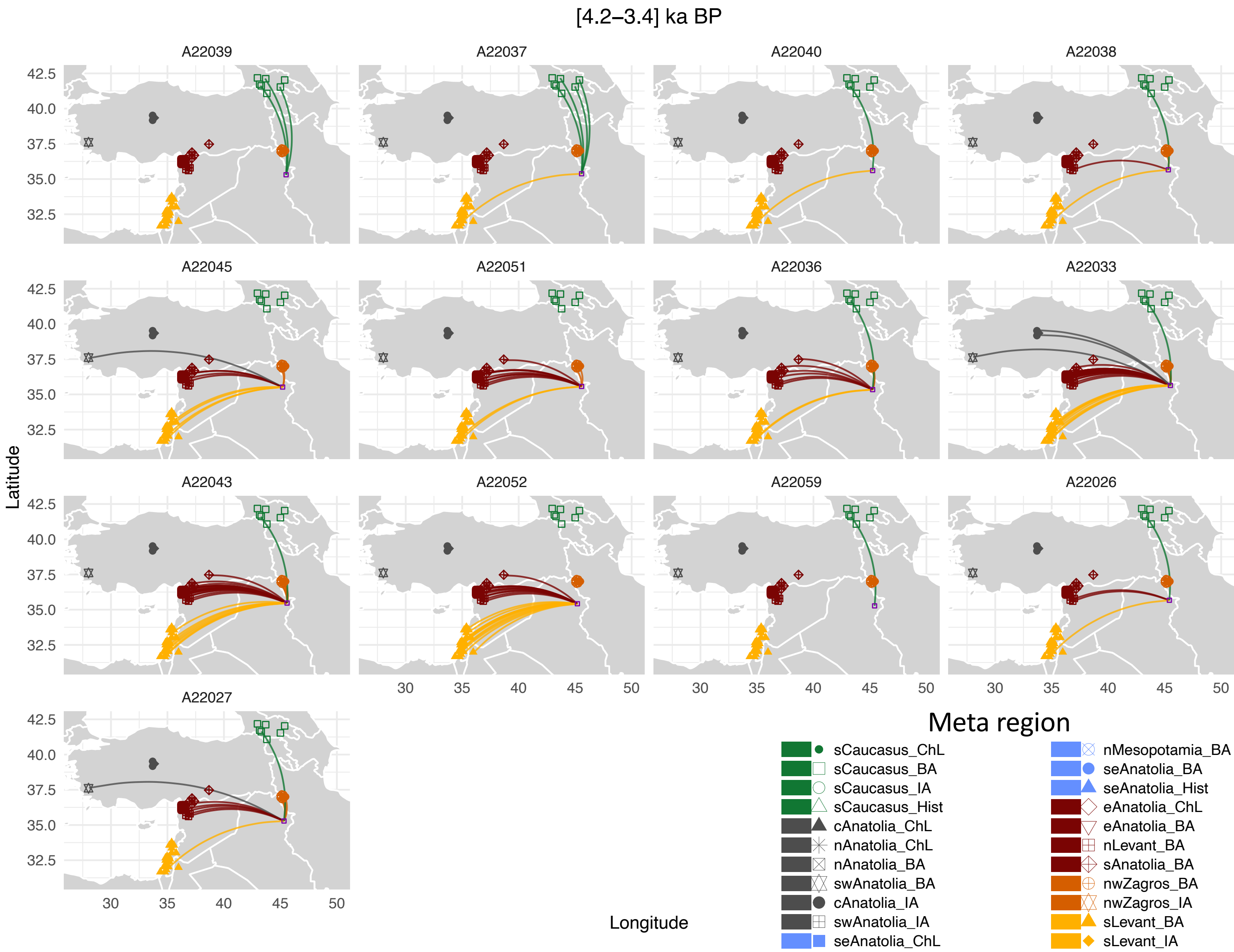
[4.2–3.4] ka BP
A22039
A22037
A22040
A22038
A22045
A22051
A22036
A22033
A22043
A22052
A22059
A22026
A22027
Latitude
Longitude
42.5
40.0
37.5
35.0
32.5
30
35
40
45
50
Meta region
sCaucasus_ChL
sCaucasus_BA
sCaucasus_IA
sCaucasus_Hist
cAnatolia_ChL
nAnatolia_ChL
nAnatolia_BA
swAnatolia_BA
cAnatolia_IA
swAnatolia_IA
seAnatolia_ChL
nMesopotamia_BA
seAnatolia_BA
seAnatolia_Hist
eAnatolia_ChL
eAnatolia_BA
nLevant_BA
sAnatolia_BA
nwZagros_BA
nwZagros_IA
sLevant_BA
sLevant_IA

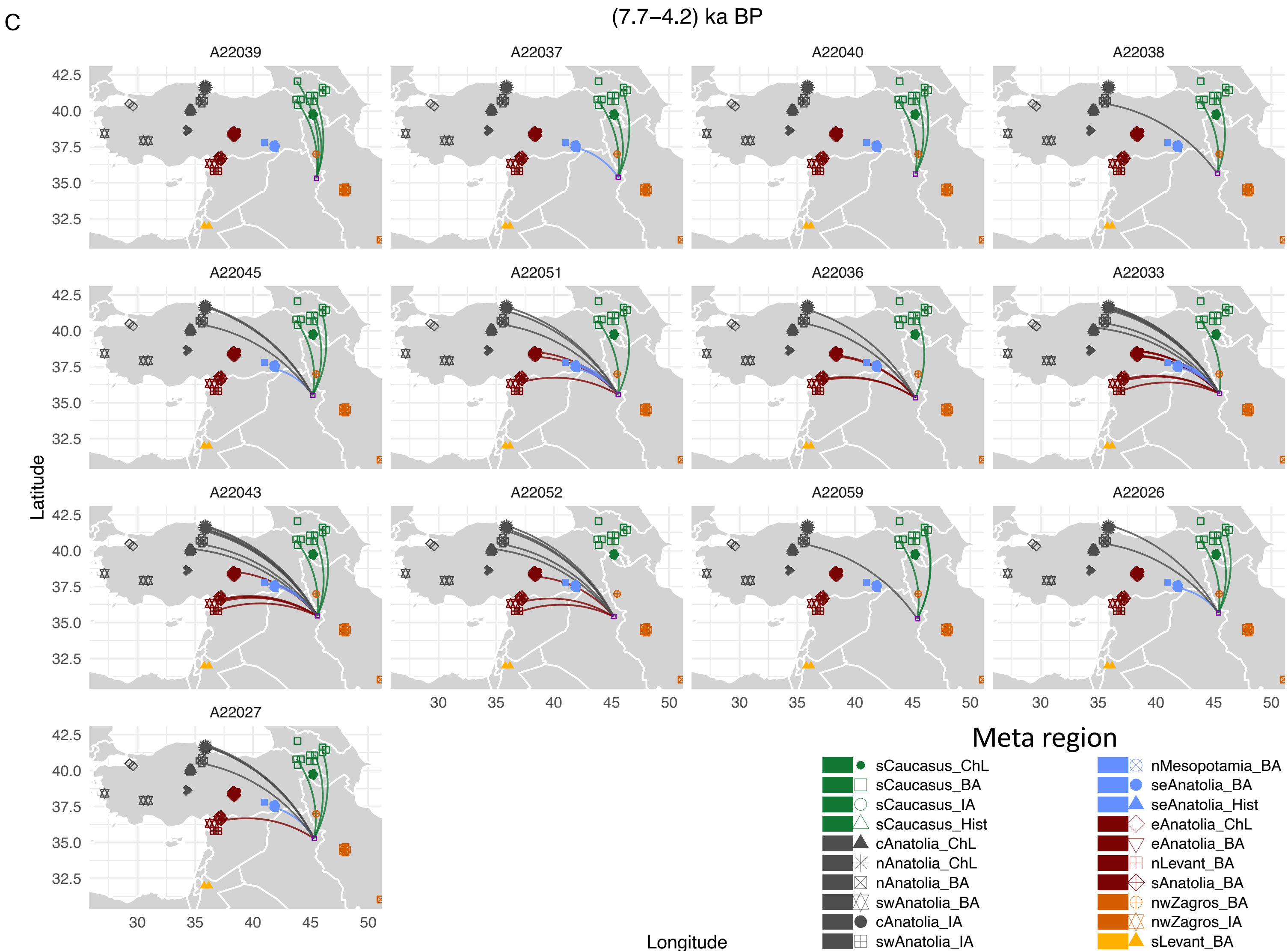

Fig. S7: Spatiotemporal qpWave individual-clade connections.
Maps displaying significant genetic cladality between Bakr Awa individuals and ancient populations from surrounding regions across three time periods: A = (3.4–1.2] ka BP, B = [4.2–3.4] ka BP, and C = (7.7–4.2) ka BP. Lines connect a Bakr Awa individual to a test individual if they form a statistical clade (p-value ≥ 0.05 after Benjamini-Hochberg correction) in pairwise qpWave analysis relative to a set of outgroups.

A

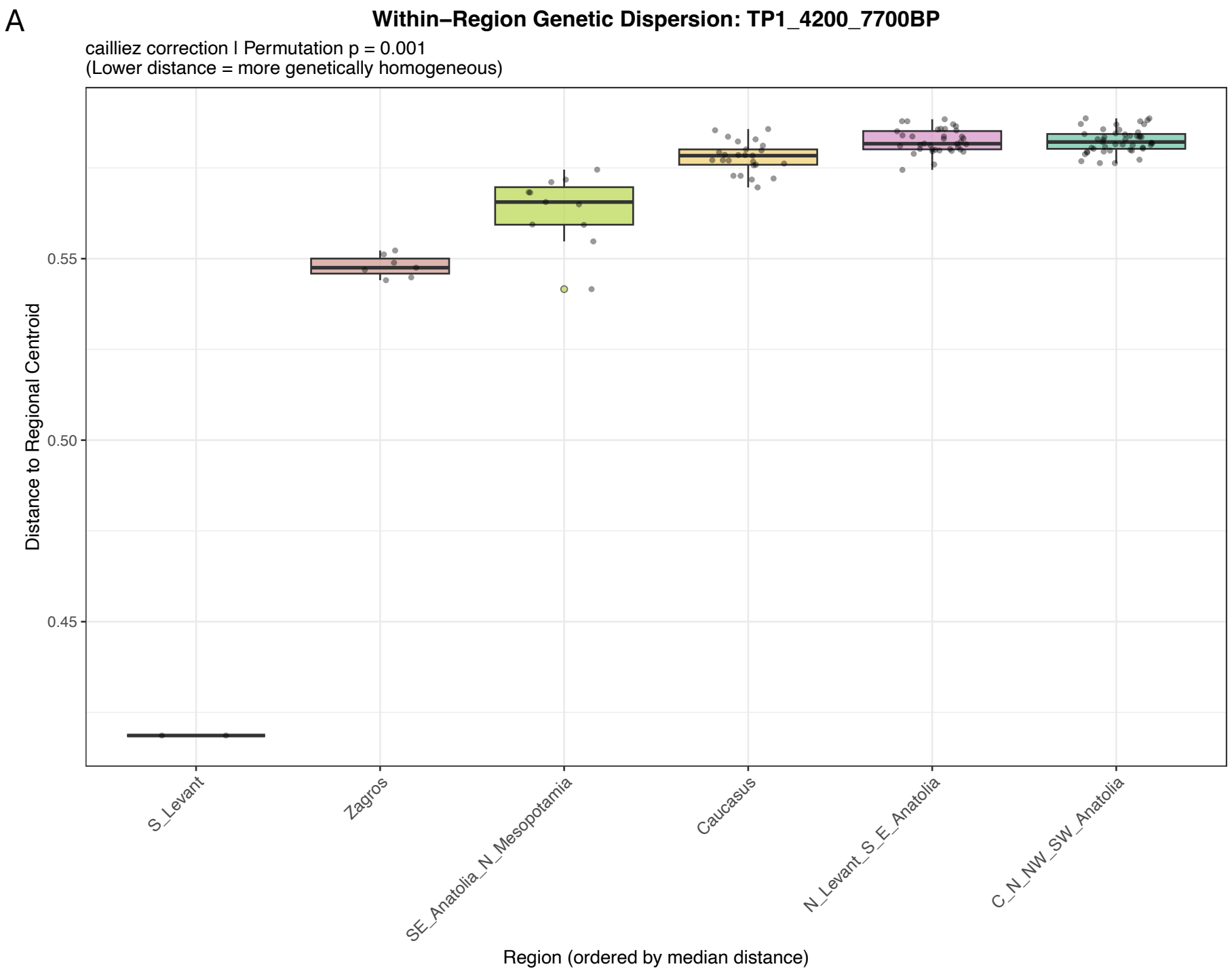
Within–Region Genetic Dispersion: TP1_4200_7700BP
cailliez correction | Permutation p = 0.001
(Lower distance = more genetically homogeneous)
Distance to Regional Centroid
0.55
0.50
0.45
S_Levant
Zagros
SE_Anatolia_N_Mesopotamia
Caucasus
N_Levant_S_E_Anatolia
C_N_NW_SW_Anatolia
Region (ordered by median distance)

B

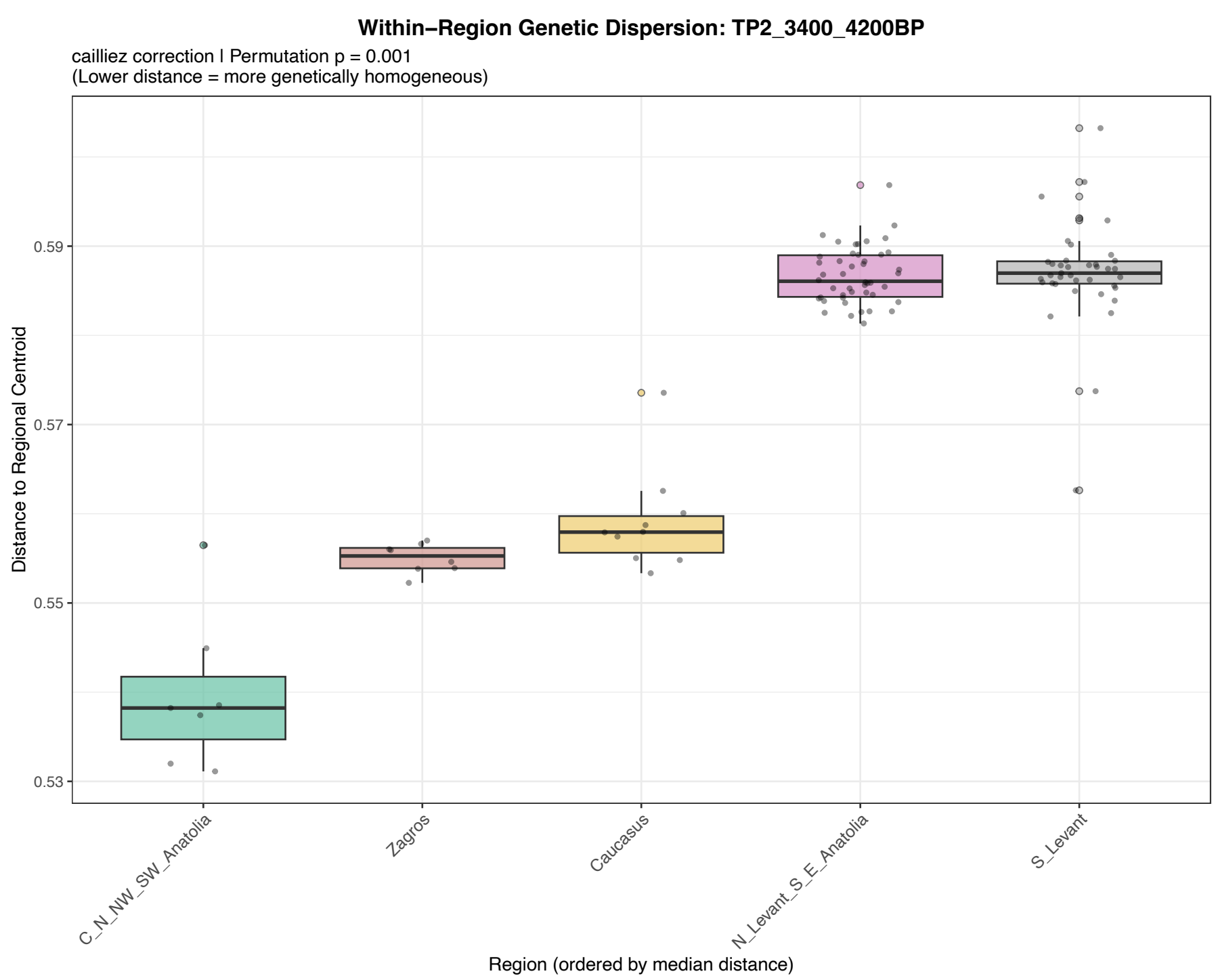
Within–Region Genetic Dispersion: TP2_3400_4200BP
cailliez correction | Permutation p = 0.001
(Lower distance = more genetically homogeneous)
Distance to Regional Centroid
0.59
0.57
0.55
0.53
C_N_NW_SW_Anatolia
Zagros
Caucasus
N_Levant_S_E_Anatolia
S_Levant
Region (ordered by median distance)

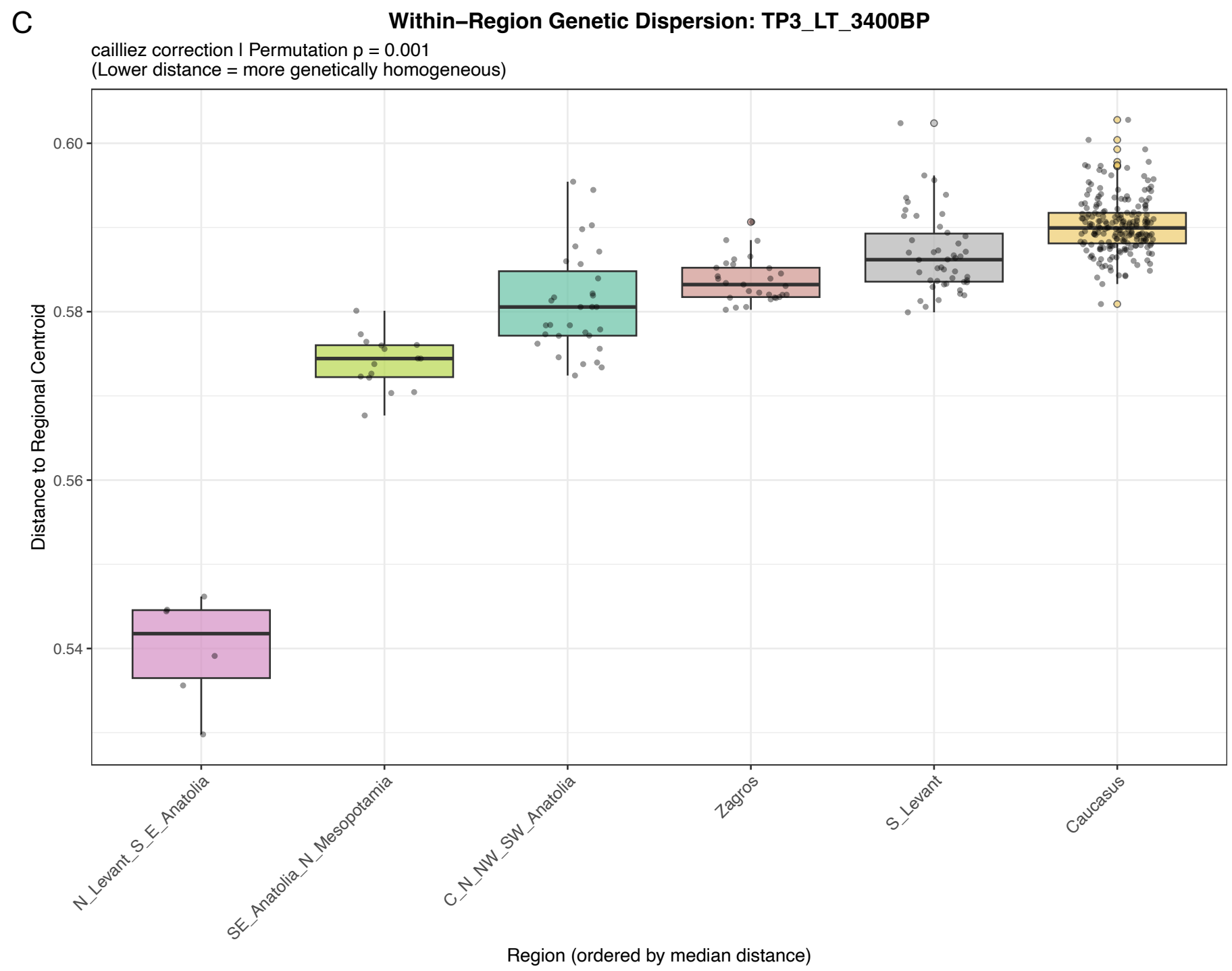

Fig. S8: Within-region distribution of individual outgroup f_3-statistic.
Boxplots showing the distribution of genetic distance (1 - f_3) to the regional centroid for six meta-regions: Zagros, S_Levant, N_Levant/SE_Anatolia, Caucasus, C/N/NW/SW_Anatolia, and SE_Anatolia/N_Mesopotamia. The plots are stratified by time period (A = TP1: 4200–7700 BP; B = TP2: 3400–4200 BP; C = TP3: <3400 BP). Lower distance values indicate higher genetic homogeneity within the region. These distributions demonstrate significant differences in genetic dispersion across regions.

[4.2–3.4] ka BP

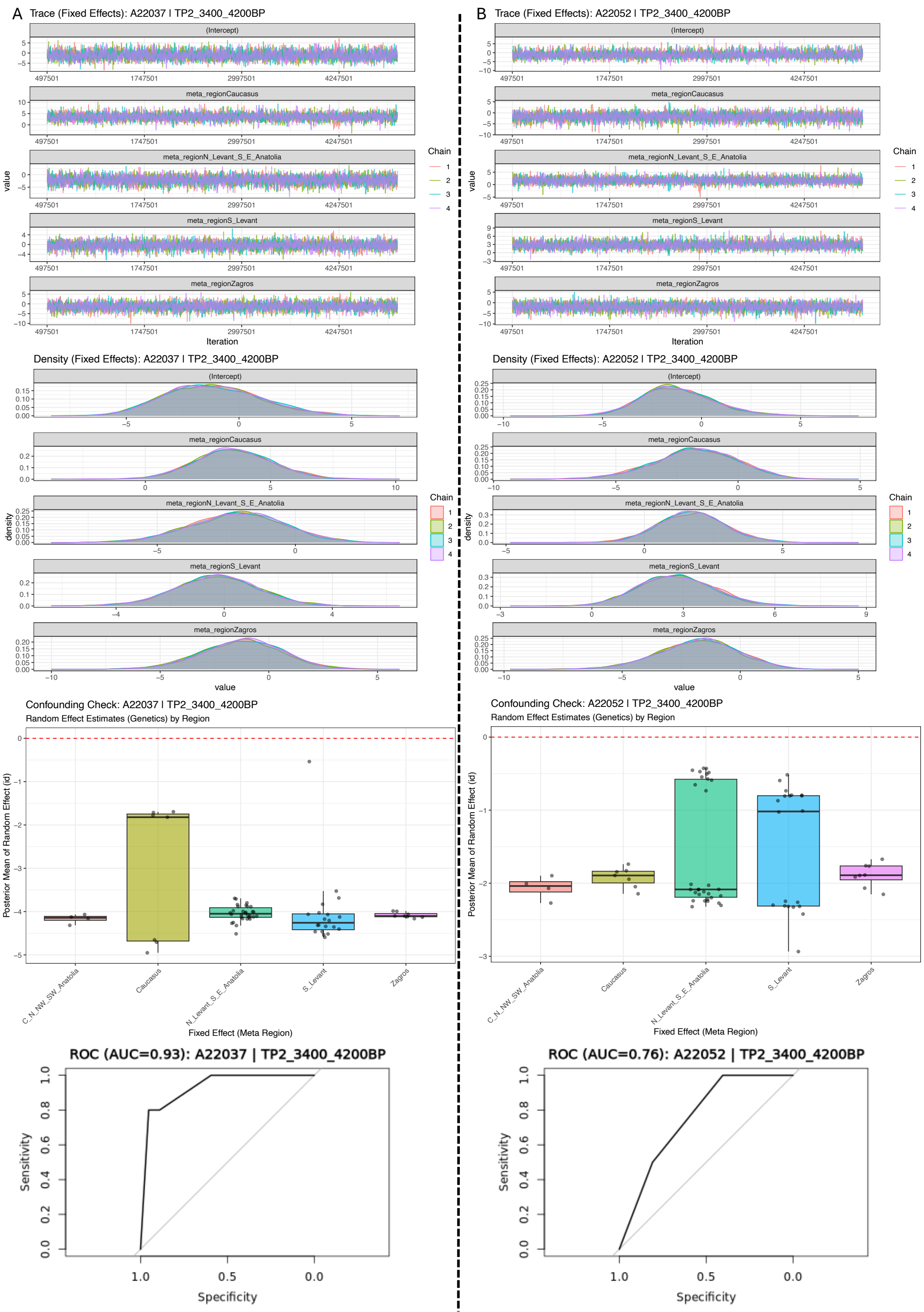
A
Trace (Fixed Effects): A22037 I TP2_3400_4200BP
(Intercept)
meta_regionCaucasus
meta_regionN_Levant_S_E_Anatolia
meta_regionS_Levant
meta_regionZagros
value
Iteration
Chain
1
2
3
4
Density (Fixed Effects): A22037 I TP2_3400_4200BP
density
value
Confounding Check: A22037 I TP2_3400_4200BP
Random Effect Estimates (Genetics) by Region
Posterior Mean of Random Effect (id)
C_N_NW_SW_Anatolia
Caucasus
N_Levant_S_E_Anatolia
S_Levant
Zagros
Fixed Effect (Meta Region)
ROC (AUC=0.93): A22037 | TP2_3400_4200BP
Sensitivity
Specificity
B
Trace (Fixed Effects): A22052 I TP2_3400_4200BP
Density (Fixed Effects): A22052 I TP2_3400_4200BP
Confounding Check: A22052 I TP2_3400_4200BP
Random Effect Estimates (Genetics) by Region
ROC (AUC=0.76): A22052 | TP2_3400_4200BP

(3.4–1.2] ka BP

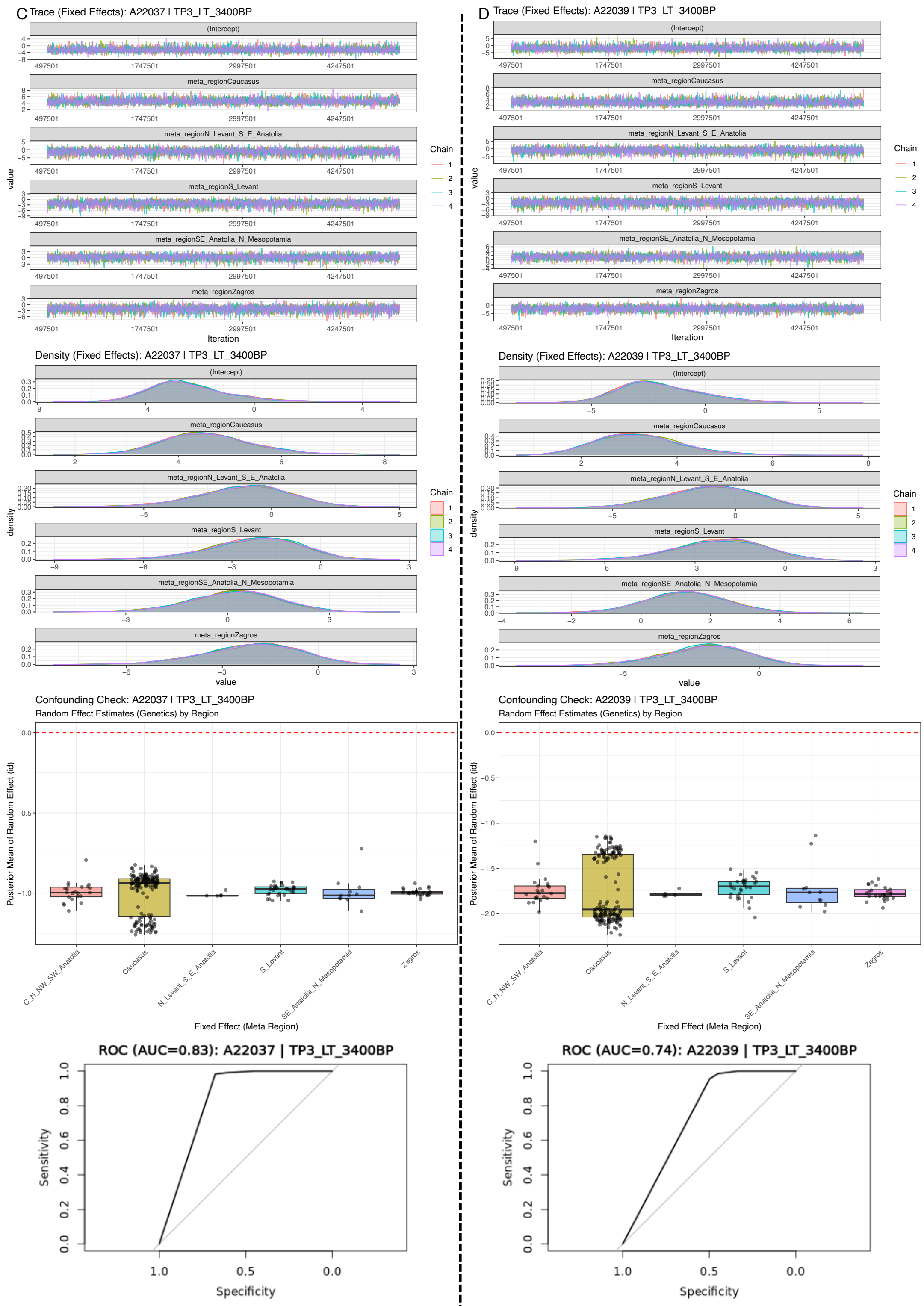
C
Trace (Fixed Effects): A22037 | TP3_LT_3400BP
(Intercept)
meta_regionCaucasus
meta_regionN_Levant_S_E_Anatolia
meta_regionS_Levant
meta_regionSE_Anatolia_N_Mesopotamia
meta_regionZagros
value
Iteration
Chain
Density (Fixed Effects): A22037 | TP3_LT_3400BP
density
value
Confounding Check: A22037 | TP3_LT_3400BP
Random Effect Estimates (Genetics) by Region
Posterior Mean of Random Effect (id)
C_N_NW_SW_Anatolia
Caucasus
N_Levant_S_E_Anatolia
S_Levant
SE_Anatolia_N_Mesopotamia
Zagros
Fixed Effect (Meta Region)
ROC (AUC=0.83): A22037 | TP3_LT_3400BP
Sensitivity
Specificity
D
Trace (Fixed Effects): A22039 | TP3_LT_3400BP
Density (Fixed Effects): A22039 | TP3_LT_3400BP
Confounding Check: A22039 | TP3_LT_3400BP
Random Effect Estimates (Genetics) by Region
ROC (AUC=0.74): A22039 | TP3_LT_3400BP

(3.4–1.2] ka BP

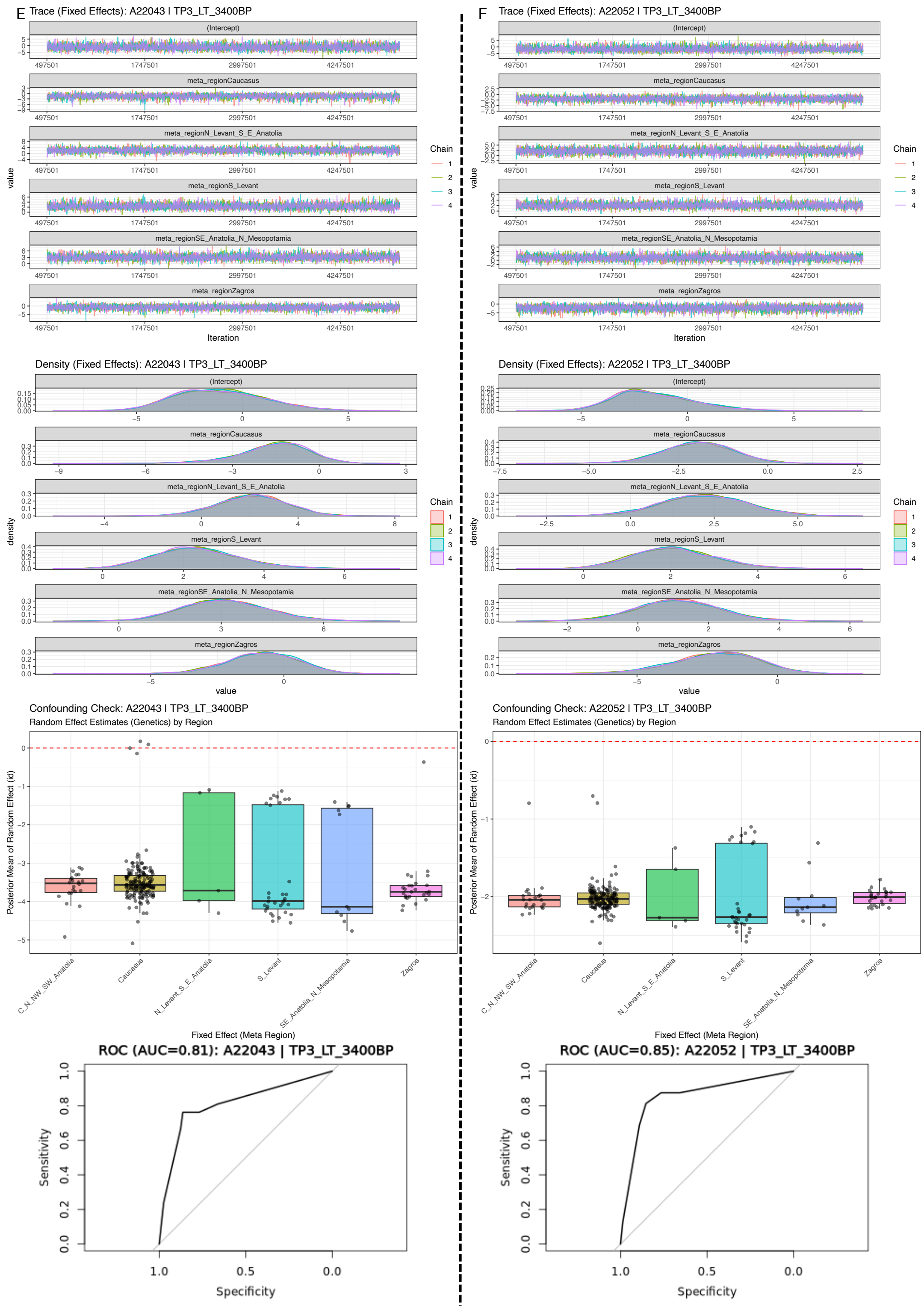
E
Trace (Fixed Effects): A22043 | TP3_LT_3400BP
(Intercept)
meta_regionCaucasus
meta_regionN_Levant_S_E_Anatolia
meta_regionS_Levant
meta_regionSE_Anatolia_N_Mesopotamia
meta_regionZagros
value
Iteration
Chain
Density (Fixed Effects): A22043 | TP3_LT_3400BP
density
value
Confounding Check: A22043 | TP3_LT_3400BP
Random Effect Estimates (Genetics) by Region
Posterior Mean of Random Effect (id)
C_N_NW_SW_Anatolia
Caucasus
N_Levant_S_E_Anatolia
S_Levant
SE_Anatolia_N_Mesopotamia
Zagros
Fixed Effect (Meta Region)
ROC (AUC=0.81): A22043 | TP3_LT_3400BP
Sensitivity
Specificity
F
Trace (Fixed Effects): A22052 | TP3_LT_3400BP
Density (Fixed Effects): A22052 | TP3_LT_3400BP
Confounding Check: A22052 | TP3_LT_3400BP
Random Effect Estimates (Genetics) by Region
ROC (AUC=0.85): A22052 | TP3_LT_3400BP

(3.4–1.2] ka BP

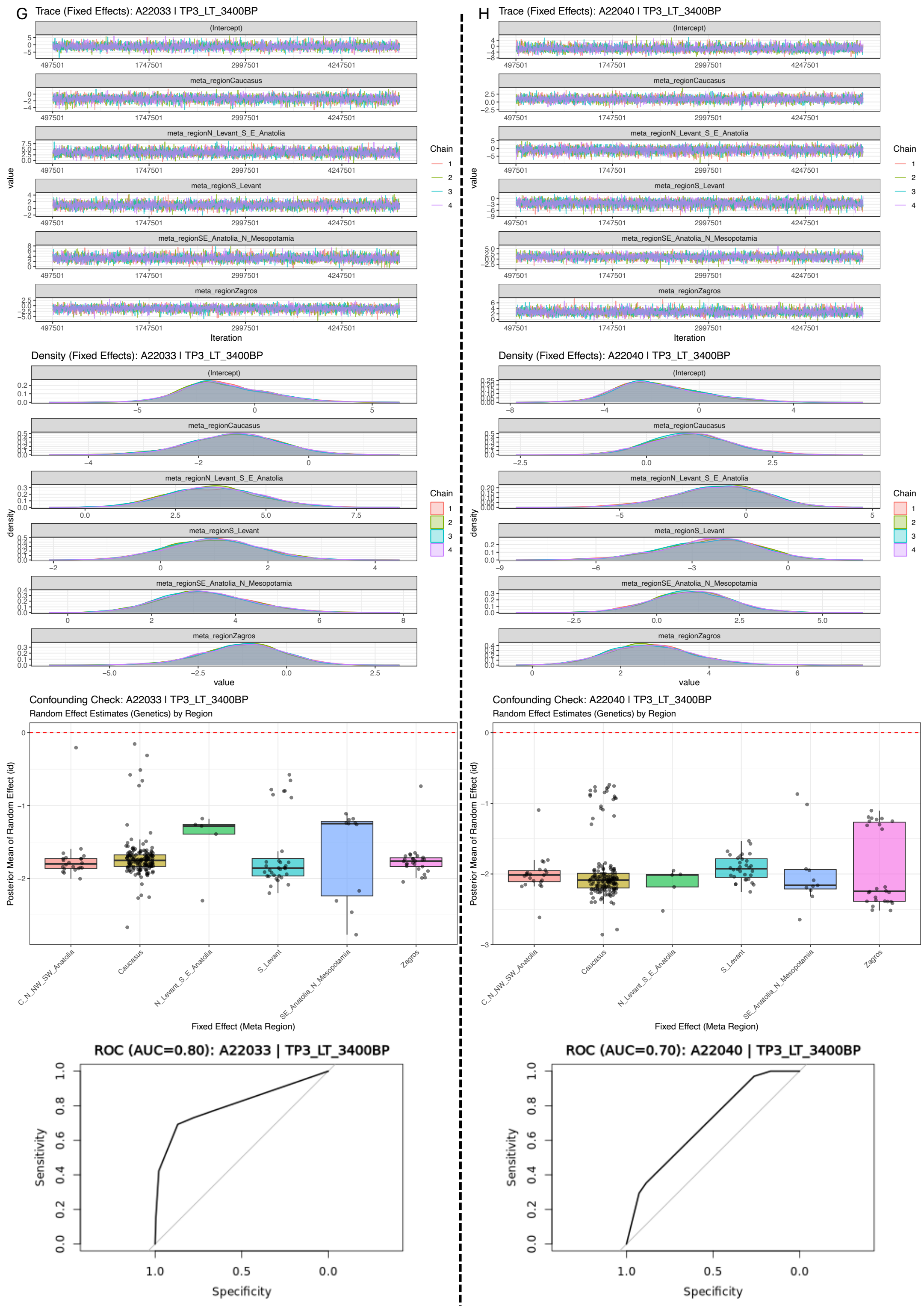
G
Trace (Fixed Effects): A22033 | TP3_LT_3400BP
(Intercept)
meta_regionCaucasus
meta_regionN_Levant_S_E_Anatolia
meta_regionS_Levant
meta_regionSE_Anatolia_N_Mesopotamia
meta_regionZagros
value
Iteration
Chain
Density (Fixed Effects): A22033 | TP3_LT_3400BP
density
value
Confounding Check: A22033 | TP3_LT_3400BP
Random Effect Estimates (Genetics) by Region
Posterior Mean of Random Effect (id)
C_N_NW_SW_Anatolia
Caucasus
N_Levant_S_E_Anatolia
S_Levant
SE_Anatolia_N_Mesopotamia
Zagros
Fixed Effect (Meta Region)
ROC (AUC=0.80): A22033 | TP3_LT_3400BP
Sensitivity
Specificity
H
Trace (Fixed Effects): A22040 | TP3_LT_3400BP
Density (Fixed Effects): A22040 | TP3_LT_3400BP
Confounding Check: A22040 | TP3_LT_3400BP
Random Effect Estimates (Genetics) by Region
ROC (AUC=0.70): A22040 | TP3_LT_3400BP

(3.4–1.2] ka BP

I

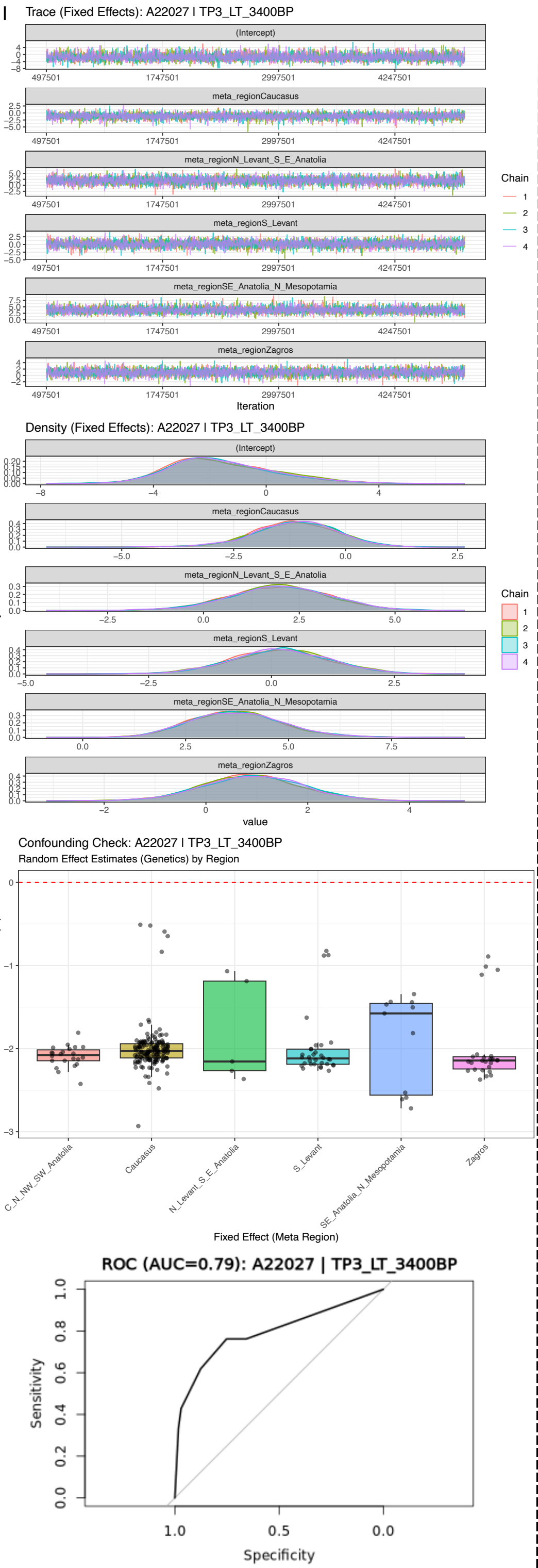
Trace (Fixed Effects): A22027 | TP3_LT_3400BP
(Intercept)
meta_regionCaucasus
meta_regionN_Levant_S_E_Anatolia
meta_regionS_Levant
meta_regionSE_Anatolia_N_Mesopotamia
meta_regionZagros
value
Iteration
Chain
1
2
3
4
Density (Fixed Effects): A22027 | TP3_LT_3400BP
density
value
Confounding Check: A22027 | TP3_LT_3400BP
Random Effect Estimates (Genetics) by Region
Posterior Mean of Random Effect (id)
C_N_NW_SW_Anatolia
Caucasus
N_Levant_S_E_Anatolia
S_Levant
SE_Anatolia_N_Mesopotamia
Zagros
Fixed Effect (Meta Region)
ROC (AUC=0.79): A22027 | TP3_LT_3400BP
Sensitivity
Specificity

J

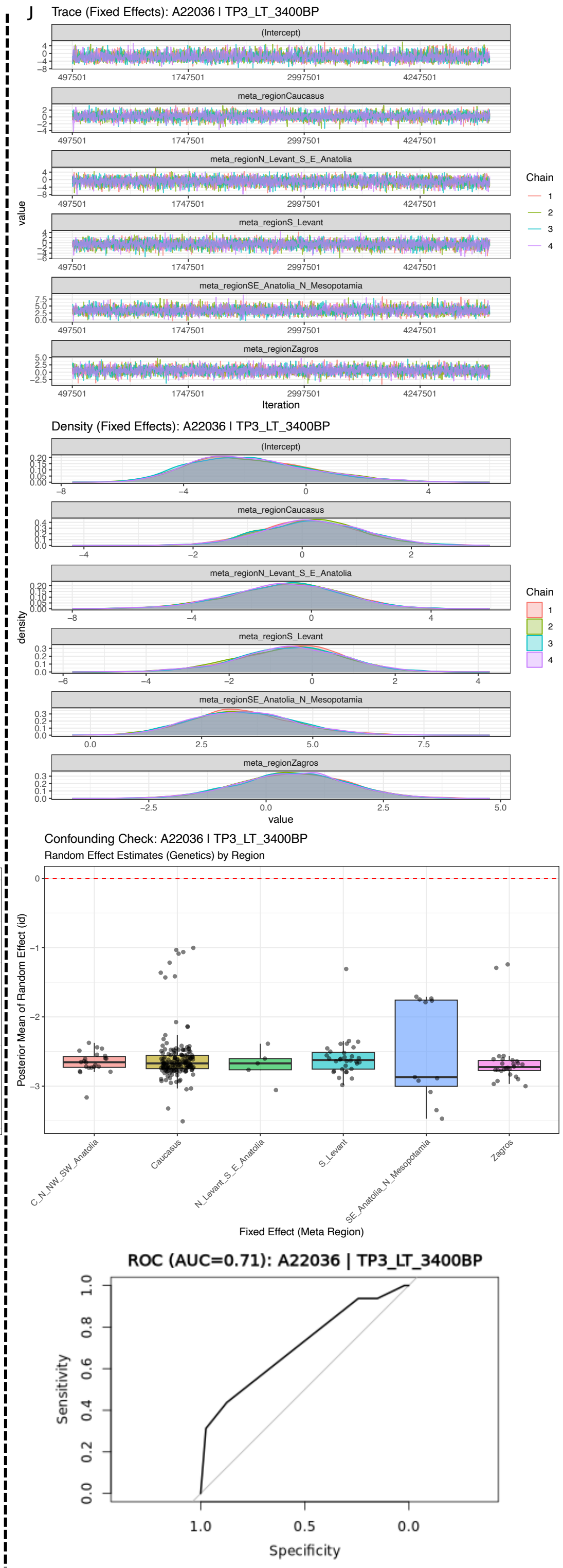
Trace (Fixed Effects): A22036 | TP3_LT_3400BP
(Intercept)
meta_regionCaucasus
meta_regionN_Levant_S_E_Anatolia
meta_regionS_Levant
meta_regionSE_Anatolia_N_Mesopotamia
meta_regionZagros
value
Iteration
Chain
1
2
3
4
Density (Fixed Effects): A22036 | TP3_LT_3400BP
density
value
Confounding Check: A22036 | TP3_LT_3400BP
Random Effect Estimates (Genetics) by Region
Posterior Mean of Random Effect (id)
C_N_NW_SW_Anatolia
Caucasus
N_Levant_S_E_Anatolia
S_Levant
SE_Anatolia_N_Mesopotamia
Zagros
Fixed Effect (Meta Region)
ROC (AUC=0.71): A22036 | TP3_LT_3400BP
Sensitivity
Specificity

(3.4–1.2] ka BP

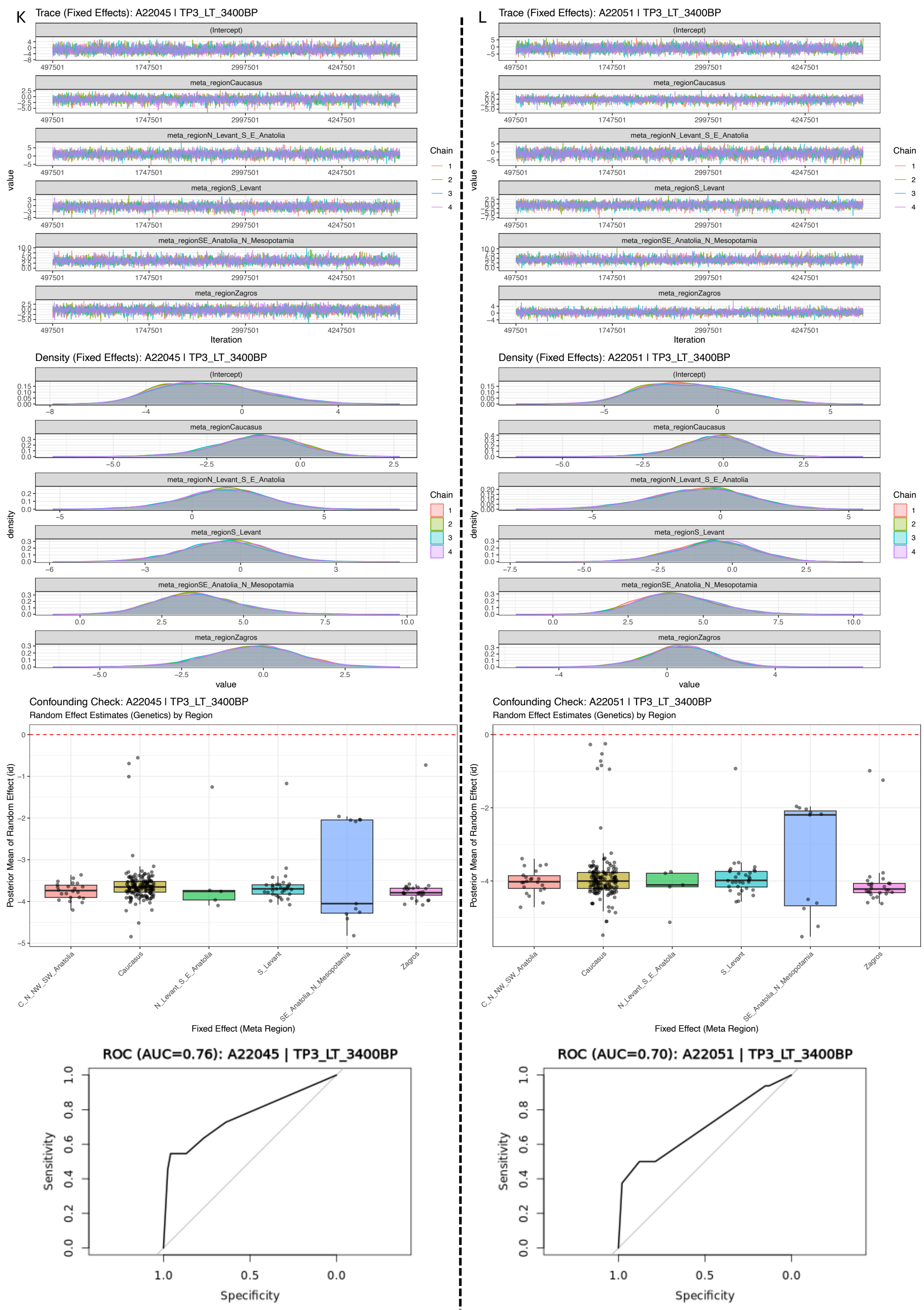

Fig. S9: Bayesian Generalized Linear Mixed Model (BGLMM) diagnostics and confounding checks.

This figure displays diagnostic plots for the BGLMM analysis, which tested the association

between significant qpWave admixture signals in Bakr Awa individuals and specific geographic meta-regions. Each panel (A–L) corresponds to a separate sample and includes four diagnostic subplots: 1) **Trace plots** for Fixed Effects (meta-regions), demonstrating MCMC chain convergence across four parallel runs. 2) **Density plots** showing the posterior probability distribution of the effect size for each meta-region. 3) **Confounding checks**, visualized as boxplots with jittered points, comparing the Fixed Effect (meta-region) against the posterior mean of the Random Effect (genetic ID). These checks confirm that the regional enrichments are not driven solely by background genetic structure, which the model captures via an outgroup f_3-statistic kinship matrix. 4) **ROC curves**, reporting the Area Under the Curve (AUC) to evaluate the model's predictive accuracy. Panels A–B represent results from 4.2–3.4 ka BP, while panels C–L represent results from 3.4–1.2 ka BP periods.

A

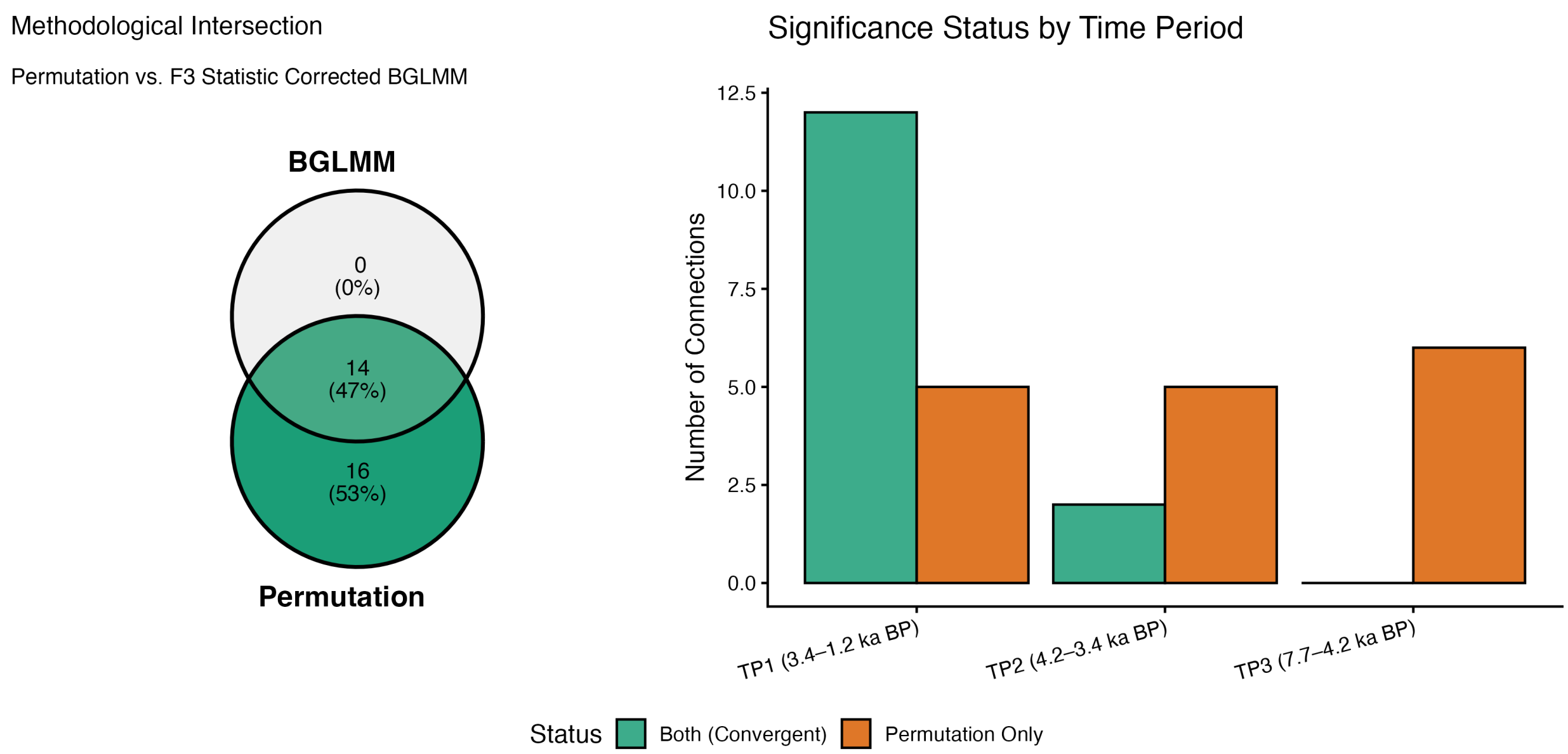
Methodological Intersection
Permutation vs. F3 Statistic Corrected BGLMM
BGLMM
0
(0%)
14
(47%)
16
(53%)
Permutation
Significance Status by Time Period
Number of Connections
12.5
10.0
7.5
5.0
2.5
0.0
TP1 (3.4–1.2 ka BP)
TP2 (4.2–3.4 ka BP)
TP3 (7.7–4.2 ka BP)
Status
Both (Convergent)
Permutation Only

B

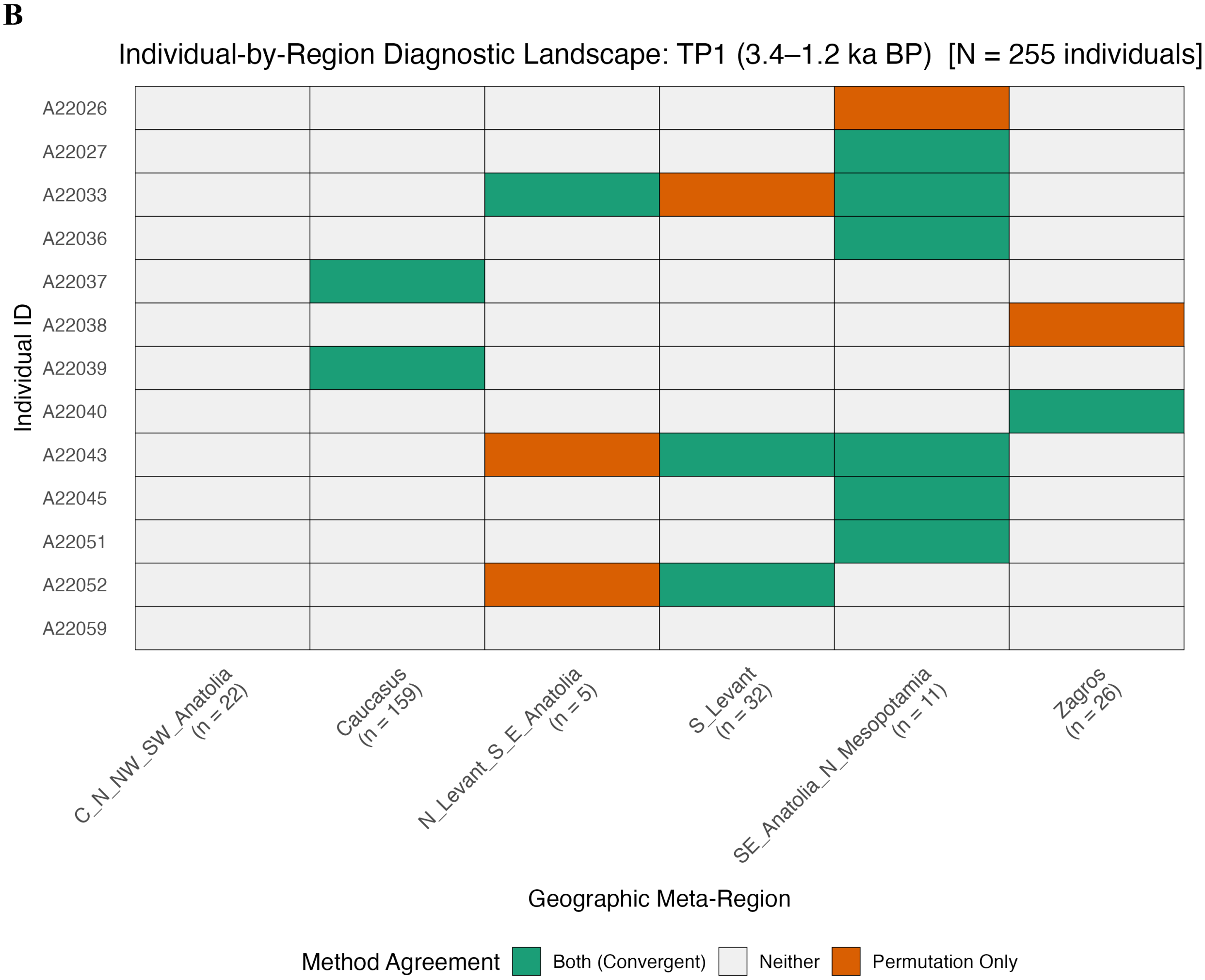
Individual-by-Region Diagnostic Landscape: TP1 (3.4–1.2 ka BP) [N = 255 individuals]
Individual ID
A22026
A22027
A22033
A22036
A22037
A22038
A22039
A22040
A22043
A22045
A22051
A22052
A22059
C_N_NW_SW_Anatolia (n = 22)
Caucasus (n = 159)
N_Levant_S_E_Anatolia (n = 5)
S_Levant (n = 32)
SE_Anatolia_N_Mesopotamia (n = 11)
Zagros (n = 26)
Geographic Meta-Region
Method Agreement
Both (Convergent)
Neither
Permutation Only

C

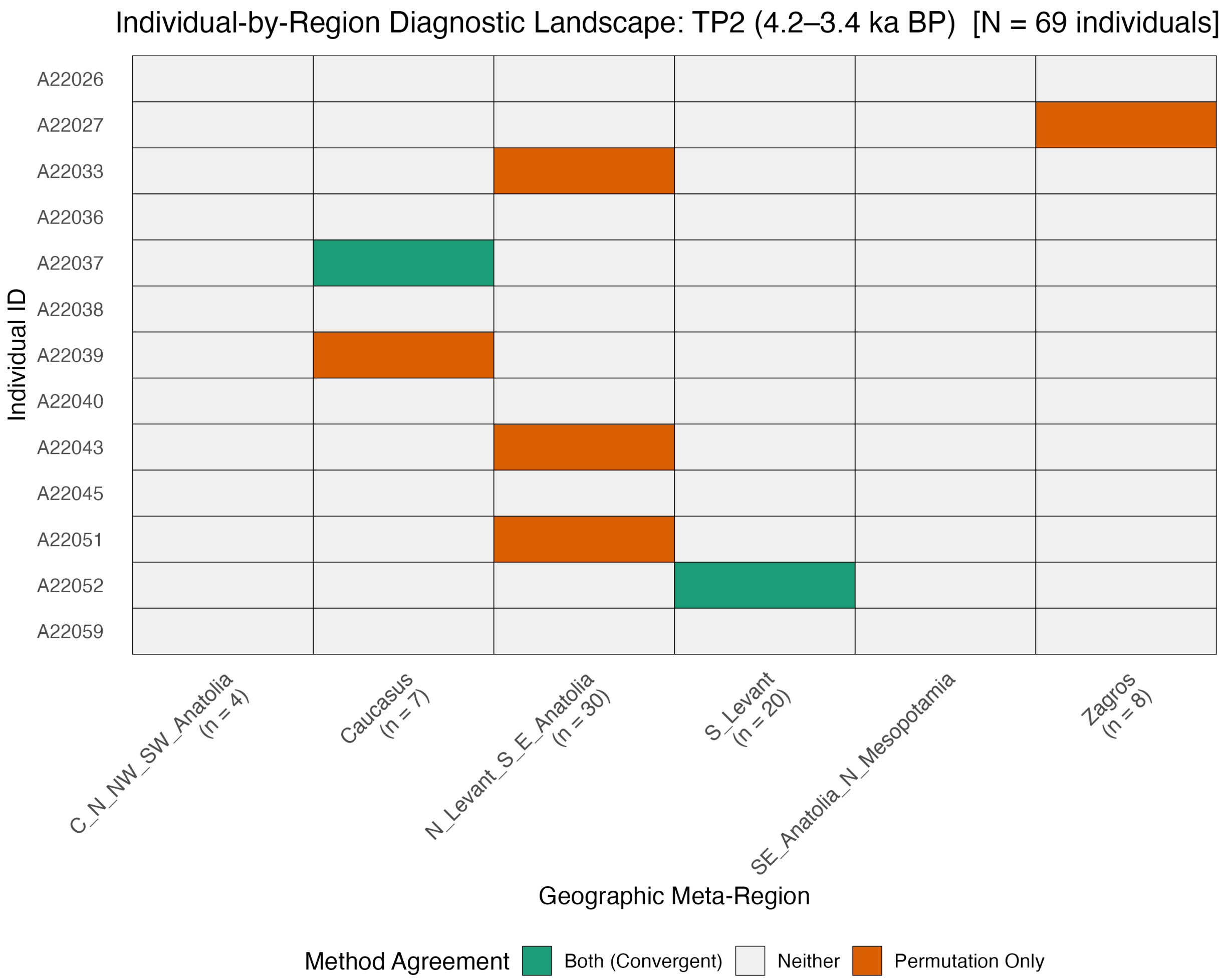
Individual-by-Region Diagnostic Landscape: TP2 (4.2–3.4 ka BP) [N = 69 individuals]
Individual ID
A22026
A22027
A22033
A22036
A22037
A22038
A22039
A22040
A22043
A22045
A22051
A22052
A22059
C_N_NW_SW_Anatolia (n = 4)
Caucasus (n = 7)
N_Levant_S_E_Anatolia (n = 30)
S_Levant (n = 20)
SE_Anatolia_N_Mesopotamia
Zagros (n = 8)
Geographic Meta-Region
Method Agreement
Both (Convergent)
Neither
Permutation Only

D

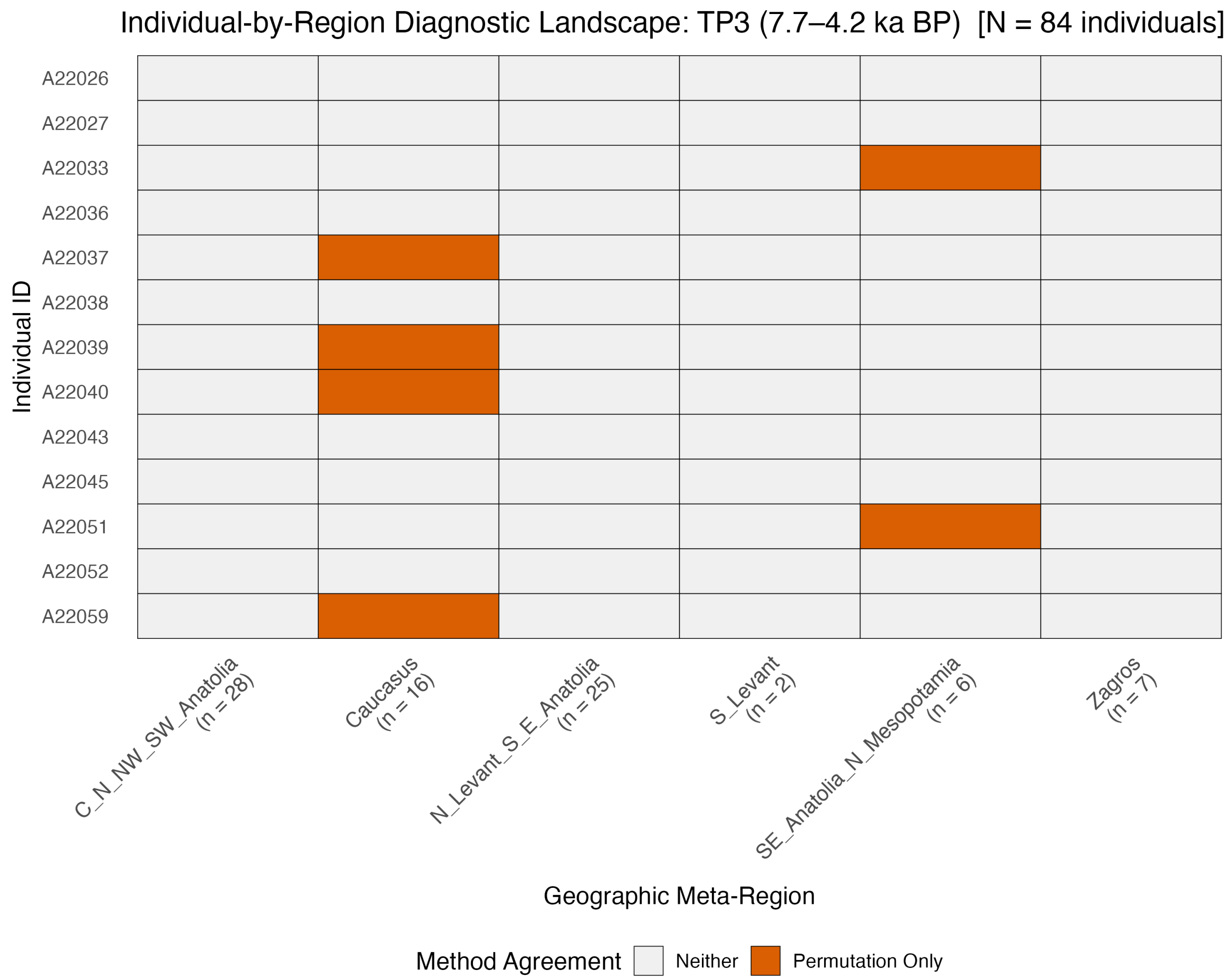

Fig. S10: Comparison between permutation and BGLMM approach to qpWave enrichment tests.
We compared affinity signals across 13 Bakr Awa individuals, three time periods (TP1: 4.2–7.7 KBP, TP2: 3.4–4.2 KBP, TP3: <3.4 KBP), and five meta-regions using two methods: a Permutation test (significance at $p < 0.05$) and a Bayesian Generalized Linear Mixed Model (BGLMM) incorporating outgroup $f3$-statistics (significance at pMCMC < 0.05 and 95% CI excluding zero). This figure evaluates how correcting for baseline genetic kinship (via outgroup $f3$-statistics in a BGLMM) filters signals initially identified by a Permutation approach. **(A) Methodological intersection and significance status.** The Venn diagram (left) illustrates the total overlap of all significant tests, revealing that the BGLMM acts as a strict, conservative filter. The bar chart (right) stratifies their shared and unique connections by time period. **(B-D) Comparison by Time Period.** Three tile plots map the analytical outcome of every individual-region pair. Colors indicate methodological agreement: green represents robust signals significant in both models ("Both Convergent"); orange highlights potential false positives driven by shared baseline ancestry between the individuals in the corresponding spatiotemporal window ("Permutation Only"); and gray indicates non-significant pairings ("Neither").

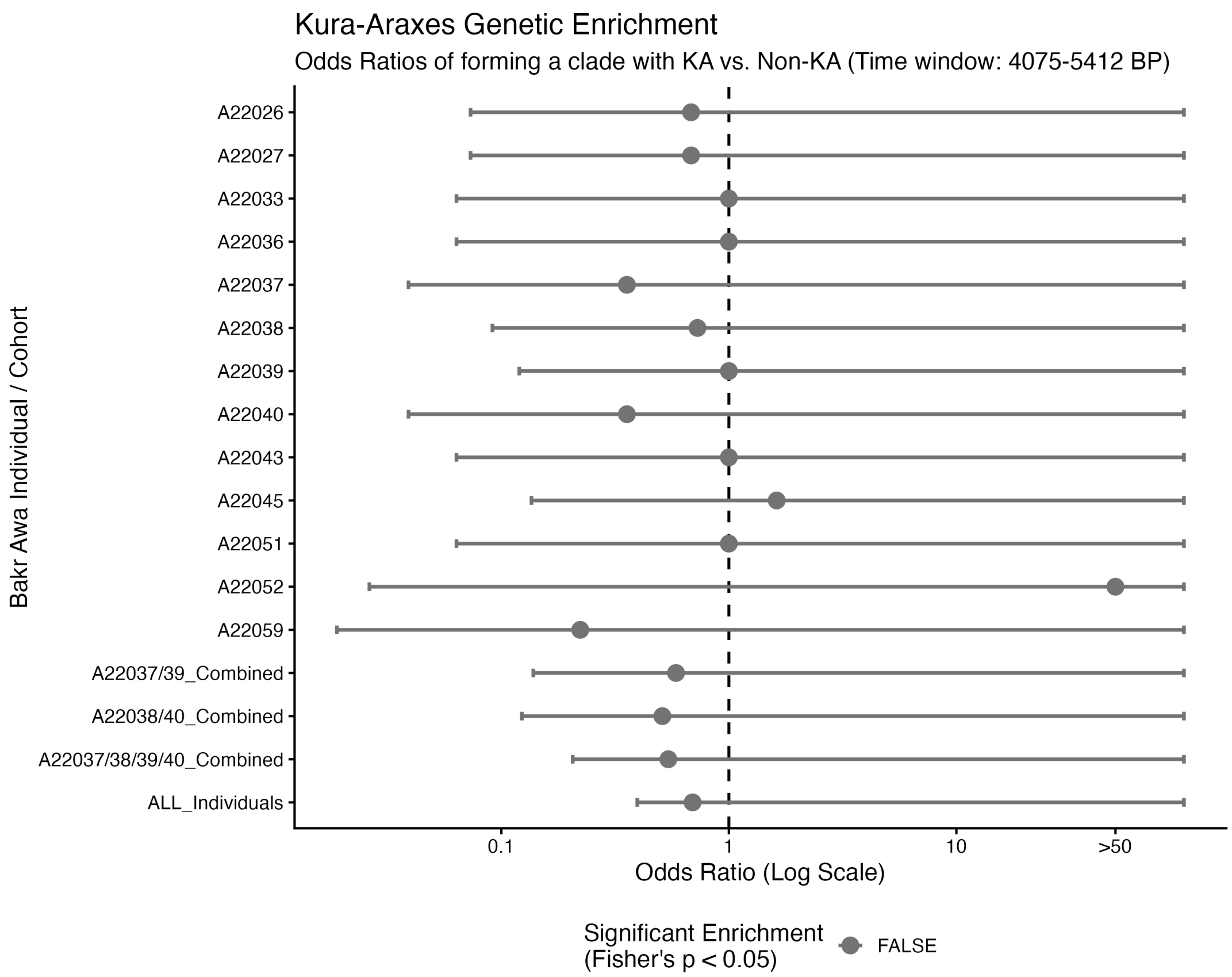

Fig. S11: Kura-Araxes Genetic Enrichment Analysis.
This forest plot visualizes whether individual Bakr Awa samples exhibit a statistically significant enrichment of genetic connections to Southern Caucasus individuals from Kura-Araxes (KA) archaeological contexts, relative to contemporaneous individuals from the same geographic region. We set an analysis window based on the known dates of the sampled KA individuals (4325–5162 BP) plus a 250-year buffer. As a result, the test only included KA and non-KA individuals dated between 4075 and 5412 BP. For each Bakr Awa individual, genetic affinities to the reference pool were evaluated via qpWave, where a successful connection (clade) was defined as a Benjamini-Hochberg corrected p-value >= 0.05. A 2x2 contingency table was constructed for each individual, tallying connection status (Connected vs. Not Connected) against archaeological context (KA vs. Non-KA). To increase statistical power and test specific subsets, connection counts were also pooled to evaluate aggregate groupings (e.g., paired subsets and all individuals combined). A one-sided Fisher's Exact Test was then utilized to calculate the exact hypergeometric probability of enrichment and the corresponding Odds Ratio. Each dot represents the calculated point estimate of the Odds Ratio (OR) for a specific Bakr Awa individual, plotted on a logarithmic scale (x-axis). The color indicates statistical significance: gray denotes a lack of significant enrichment (Fisher's exact > 0.05). The horizontal lines extending from each dot represent the 95% Confidence Interval (CI) bounds for the computed Odds Ratio. The vertical dashed line represents the null hypothesis of the test (Odds Ratio = 1). An OR of 1 indicates no enrichment; it signifies that a Bakr Awa individual has an equal probability of forming a genetic clade with a KA individual as they do with a contemporaneous non-KA individual from the Southern Caucasus. Values to the right of this line (OR > 1) indicate

a positive enrichment, meaning the individual is more likely to form a clade with a KA individual than a non-KA individual. Conversely, values to the left of the line (OR < 1) indicate a depletion, meaning the individual is less likely to form a clade with a KA individual compared to a non-KA individual.

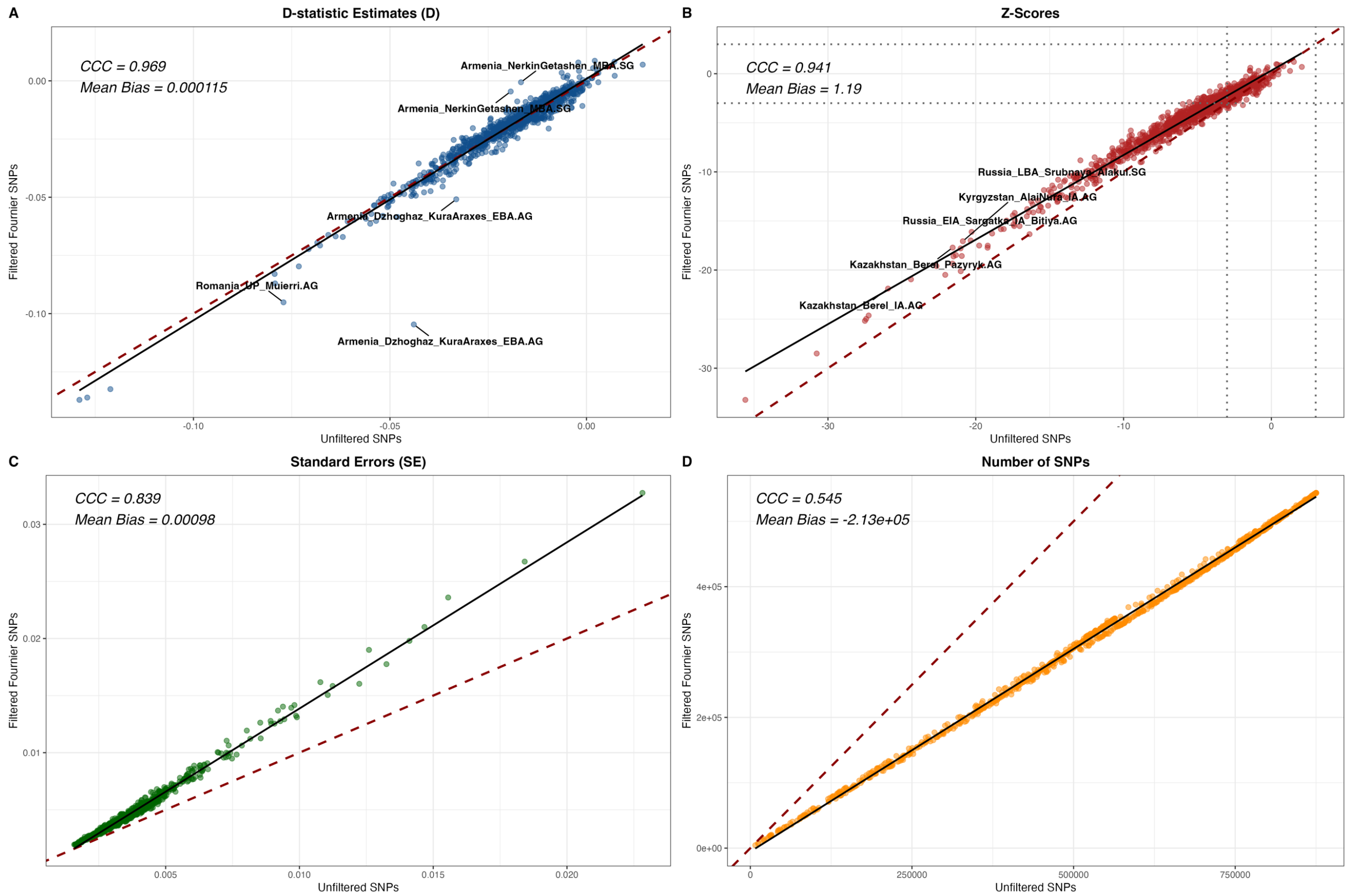
A
D-statistic Estimates (D)
CCC = 0.969
Mean Bias = 0.000115
Armenia_NerkinGetashen_MBA.SG
Armenia_NerkinGetashen_MBA.SG
Armenia_Dzhoghaz_KuraAraxes_EBA.AG
Romania_UP_Muierri.AG
Armenia_Dzhoghaz_KuraAraxes_EBA.AG
Filtered Fournier SNPs
Unfiltered SNPs
B
Z-Scores
CCC = 0.941
Mean Bias = 1.19
Russia_LBA_Srubnaya_Alakul.SG
Kyrgyzstan_AlaiNura_IA.AG
Russia_EIA_Sargatka_IA_Bitiya.AG
Kazakhstan_Berel_Pazyryk.AG
Kazakhstan_Berel_IA.AG
Filtered Fournier SNPs
Unfiltered SNPs
C
Standard Errors (SE)
CCC = 0.839
Mean Bias = 0.00098
Filtered Fournier SNPs
Unfiltered SNPs
D
Number of SNPs
CCC = 0.545
Mean Bias = -2.13e+05
Filtered Fournier SNPs
Unfiltered SNPs

E

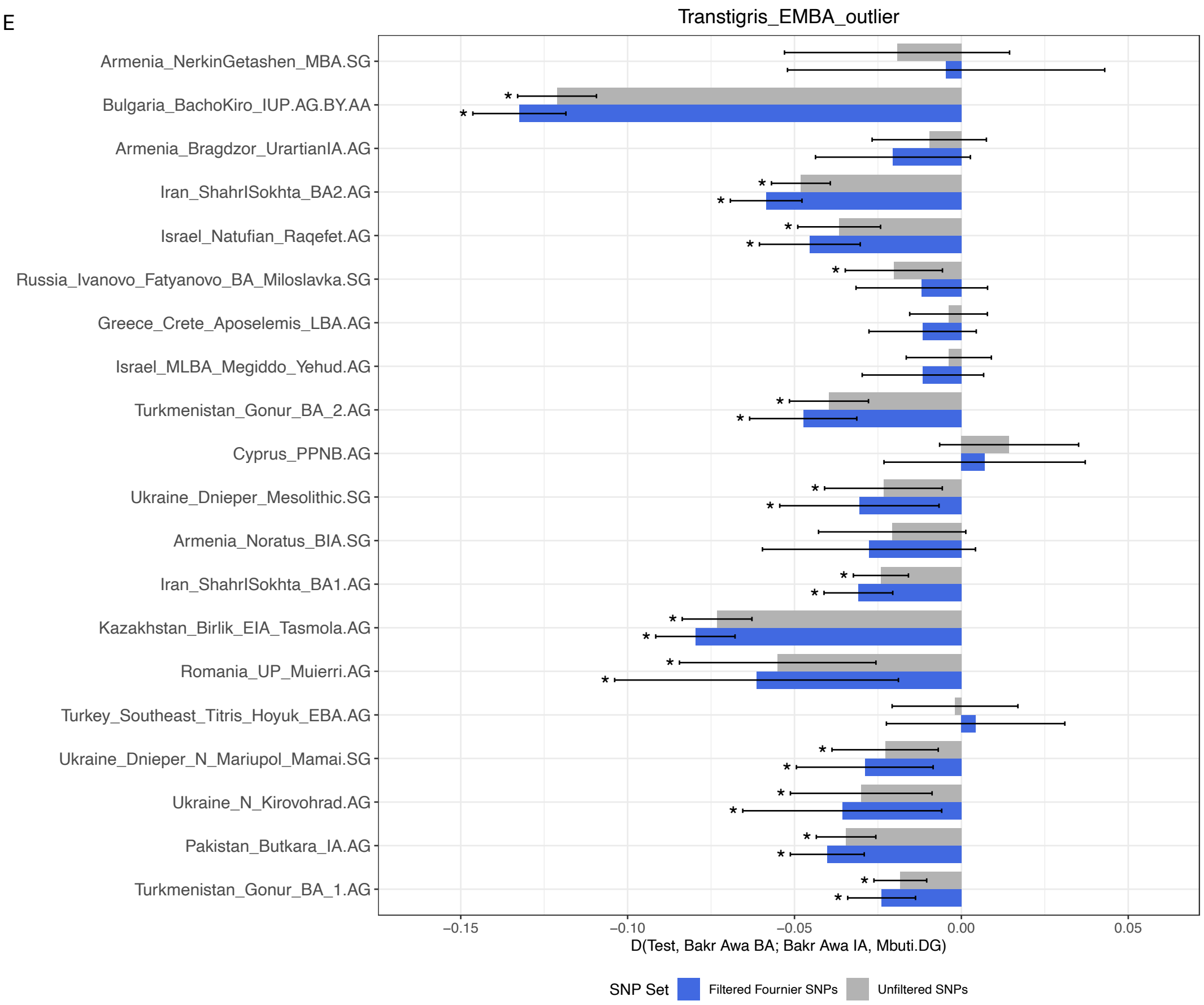
Transtigris_EMBA_outlier
Armenia_NerkinGetashen_MBA.SG
Bulgaria_BachoKiro_IUP.AG.BY.AA
Armenia_Bragdzor_UrartianIA.AG
Iran_ShahrISokhta_BA2.AG
Israel_Natufian_Raqefet.AG
Russia_Ivanovo_Fatyanovo_BA_Miloslavka.SG
Greece_Crete_Aposelemis_LBA.AG
Israel_MLBA_Megiddo_Yehud.AG
Turkmenistan_Gonur_BA_2.AG
Cyprus_PPNB.AG
Ukraine_Dnieper_Mesolithic.SG
Armenia_Noratus_BIA.SG
Iran_ShahrISokhta_BA1.AG
Kazakhstan_Birlik_EIA_Tasmola.AG
Romania_UP_Muierri.AG
Turkey_Southeast_Titris_Hoyuk_EBA.AG
Ukraine_Dnieper_N_Mariupol_Mamai.SG
Ukraine_N_Kirovohrad.AG
Pakistan_Butkara_IA.AG
Turkmenistan_Gonur_BA_1.AG
−0.15
−0.10
−0.05
0.00
0.05
D(Test, Bakr Awa BA; Bakr Awa IA, Mbuti.DG)
SNP Set
Filtered Fournier SNPs
Unfiltered SNPs

F

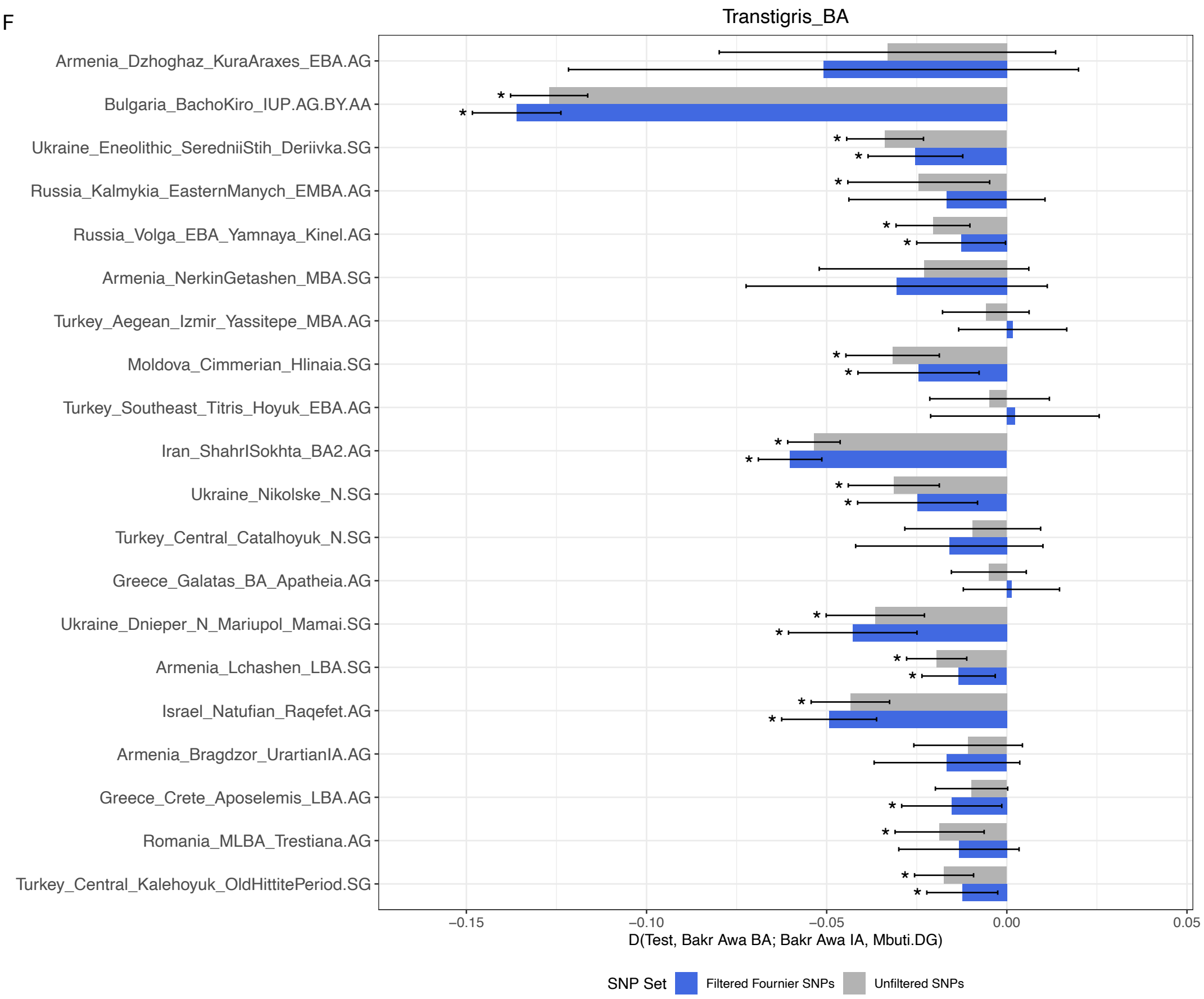
Transtigris_BA
Armenia_Dzhoghaz_KuraAraxes_EBA.AG
Bulgaria_BachoKiro_IUP.AG.BY.AA
Ukraine_Eneolithic_SeredniiStih_Deriivka.SG
Russia_Kalmykia_EasternManych_EMBA.AG
Russia_Volga_EBA_Yamnaya_Kinel.AG
Armenia_NerkinGetashen_MBA.SG
Turkey_Aegean_Izmir_Yassitepe_MBA.AG
Moldova_Cimmerian_Hlinaia.SG
Turkey_Southeast_Titris_Hoyuk_EBA.AG
Iran_ShahrISokhta_BA2.AG
Ukraine_Nikolske_N.SG
Turkey_Central_Catalhoyuk_N.SG
Greece_Galatas_BA_Apatheia.AG
Ukraine_Dnieper_N_Mariupol_Mamai.SG
Armenia_Lchashen_LBA.SG
Israel_Natufian_Raqefet.AG
Armenia_Bragdzor_UrartianIA.AG
Greece_Crete_Aposelemis_LBA.AG
Romania_MLBA_Trestiana.AG
Turkey_Central_Kalehoyuk_OldHittitePeriod.SG
−0.15
−0.10
−0.05
0.00
0.05
D(Test, Bakr Awa BA; Bakr Awa IA, Mbuti.DG)
SNP Set
Filtered Fournier SNPs
Unfiltered SNPs

G

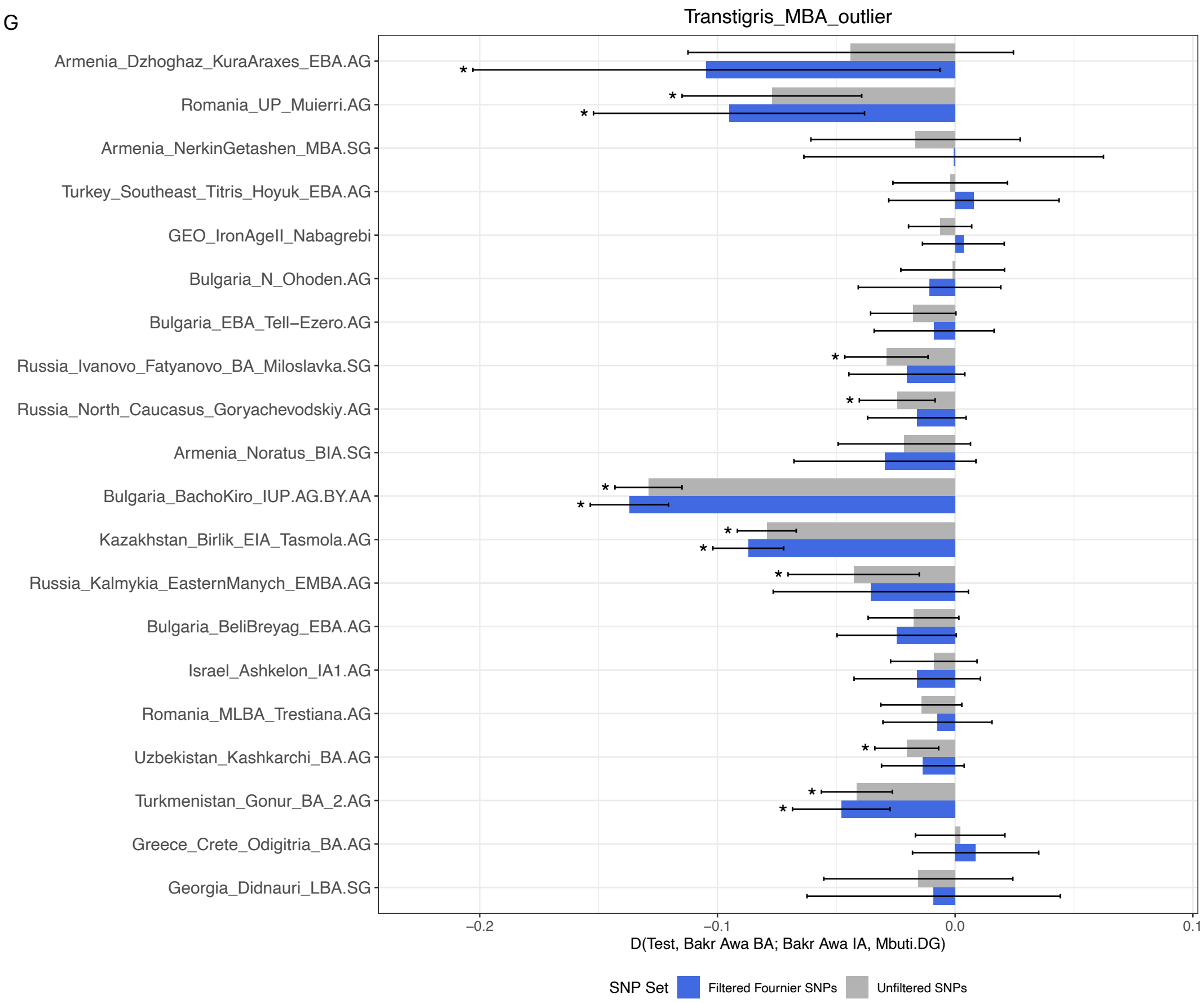

Fig. S12: Diagnostic comparison of D-statistics and statistical power between unfiltered and conservatively filtered SNP datasets.
To evaluate potential technical bias in genetic affinity, we computed *D*(Test, Transtigris; Transtigris_IA, Mbuti.DG) using the full Agilent 1240k SNP panel versus a conservatively filtered Compatibility panel (Fournier et al., 2025 (*22*)). **(A–D)** Scatter plots demonstrating the impact of SNP filtering on (A) *D*-statistic estimates, (B) Z-scores, (C) Standard Errors (SE), and (D) Number of SNPs. Deviations from the 1:1 expectation (red dashed line) are quantified using Lin's Concordance Correlation Coefficient (CCC) and Mean Bias. The plots reveal that large SNP loss as a result of filtering (D) systematically inflates standard errors (C) and shrinks Z-scores toward zero (B), while *D*-statistic estimates remain highly concordant (A). **(E–G)** Bar charts displaying the top 20 most divergent *D*-statistic estimates between unfiltered (gray) and filtered (blue) datasets for Transtigris_EMBA_outlier, Transtigris_BA, and Transtigris_MBA_outlier (|Z| > 3). Collectively, these analyses demonstrate that the 13.4% reduction in significantly negative Z-scores is likely an artifact of diminished statistical power rather than a genuine biological shift.

References

1. P. A. Miglus, “About Bakr Awa” in *The Archaeology of the Kurdistan Region of Iraq and Adjacent Regions*, K. Kopanias, J. MacGinnis, Eds. (Archaeopress Publishing LTD, Gordon House 276 Banbury Road Oxford OX2 7ED, 2016), pp. 229 – 239.

2. U. Bürger, Cultural encounters and local traditions : a discussion of the Middle Bronze Age evidence at Bakr Awa. *Zagros studies*, 67–92 (2020).

3. W. J. Arkell, George Martin Lees, 1898-1955. The Royal Society London [Preprint] (1955).

4. M. B. Al-Husaini, The Excavations at Tel Bakr-Awa. *Sumer* **18**, 140–164 (1962).

5. E. A. Speiser, Southern Kurdistan in the annals of Ashurnasirpal and today. *The Annual of the American Schools of Oriental Research* **8**, 1–41 (1926).

6. P. A. Miglus, U. Bürger, M. Heil, F. M. Stepniowski, “Ausgrabung in Bakr Āwa 2010” in *Zeitschrift für Orient-Archaologie Band 4 - 2011*, M. Ess, R. Eichmann, Eds. (Walter de Gruyter, Berlin, 2011).

7. P. A. Miglus, U. Bürger, R. A. Fetner, S. Mühl, A. Sollee, Excavation at Bakr Awa 2010 and 2011. *Iraq* **75**, 43–88 (2013).

8. J. A. Armstrong, H. Gasche, *Mesopotamian Pottery: A Guide to the Babylonian Tradition in the Second Millennium BC* (Oriental Institute of the University of Chicago, 2014).

9. P. Delougaz, Pottery from the Diyala region. *Oriental Institute publications* (1952).

10. J. Læssøe, Det første assyriske imperium: et aspekt. *Festskrift udgivet af Københavns Universitet i anledning af Universitetets aarsfest* (1966).

11. R. F. S. Starr, Nuzi: report on the excavation at Yorgan Tepa near Kirkuk, Iraq, conducted by Harvard university in conjunction with the American schools of oriental research and the University museum of Philadelphia, 1927-1931. v. 1: text. (2024).

12. S. Kroll, “Keramik urartäischer Festungen in Iran. Ein Beitrag zur Expansion Urartus in Iranisch- Azarbaidjan” in *Archäologische Mitteilungen Aus Iran, Ergänzungsband 2* (Teheran Department of the German Archaeological Institute, Berlin, 1976).

13. S. A. Paspalas, A Persianizing Cup from Lydia. *Oxf. J. Archaeol.* **19**, 135–174 (2000).

14. S. Elliott, R. Bendrey, J. Whitlam, K. R. Aziz, J. Evans, Preliminary ethnoarchaeological research on modern animal husbandry in Bestansur, Iraqi Kurdistan: Integrating animal, plant and environmental data. *Environmental archaeology* **20**, 283–303 (2015).

15. R. Mahmmud, O. Sracek, O. Mustafa, B. Čejková, I. Jačková, L. Vondrovicová, Groundwater geochemistry evolution and geogenic contaminants in the Sulaimani-Warmawa Sub-basin, Sulaimani, Kurdistan Region, Iraq. *Environmental Monitoring and Assessment* **194**, 352 (2022).

16. G. J. Bowen, The online isotopes in precipitation calculator. *Version XX Available online: http://www. waterisotopes. org (accessed on 4 April 2019)* (2015).

17. G. J. Bowen, J. Revenaugh, Interpolating the isotopic composition of modern meteoric precipitation. *Water resources research* **39** (2003).

18. S. Hillson, *Teeth* (Cambridge university press, 2005).

19. E. Lightfoot, T. C. O'Connell, On the use of biomineral oxygen isotope data to identify human migrants in the archaeological record: intra-sample variation, statistical methods and geographical considerations. *PloS one* **11**, e0153850 (2016).

20. R. D. C. Team, R: A language and environment for statistical computing. *(No Title)* (2010).

21. H. Wickham, ggplot2: elegant graphics for data analysis Springer-Verlag New York; 2009. *Preprint at* **2**, 15545–15550 (2016).

22. R. Fournier, A. P. Fulton, D. Reich. 2025. "A SNP Panel for Co-Analysis of Capture and Shotgun Ancient DNA Data." *bioRxivorg*. bioRxiv. https://doi.org/10.1101/2025.07.30.667733.
